# Enhancing the efficacy of near-infrared photoimmunotherapy through intratumoural delivery of CD44–targeting antibody–photoabsorber conjugates

**DOI:** 10.1016/j.ebiom.2025.105566

**Published:** 2025-01-22

**Authors:** Yuichi Adachi, Kotaro Miyake, Kika Ohira, Shingo Satoh, Kentaro Masuhiro, Ryuya Edahiro, Yuya Shirai, Maiko Naito, Yujiro Naito, Takayuki Shiroyama, Shohei Koyama, Haruhiko Hirata, Kota Iwahori, Izumi Nagatomo, Yoshito Takeda, Atsushi Kumanogoh

**Affiliations:** a Department of Respiratory Medicine and Clinical Immunology, Graduate School of Medicine, Osaka University, Osaka, Japan; b Department of Immunopathology, World Premier International Research Center, Initiative, Immunology, Frontier Research Center, Osaka University, Osaka, Japan; c Department of Thoracic Oncology, Osaka Habikino Medical Center, Osaka, Japan; d Department of Statistical Genetics, Graduate School of Medicine, Osaka University, Osaka, Japan; e Division of Cancer Immunology, Research Institute/Exploratory Oncology Research and Clinical Trial Center, National Cancer Center, Tokyo, Japan; f Integrated Frontier Research for Medical Science Division, Institute for Open and Transdisciplinary Research Initiatives, Osaka University, Osaka, Japan; g Center for Infectious Diseases for Education and Research, Osaka University, Osaka, Japan; h Japan Agency for Medical Research and Development – Core Research for Evolutional Science and Technology, Osaka University, Osaka, Japan; i Center for Advanced Modalities and DDS, Osaka University, Osaka, Japan

**Keywords:** Photoimmunotherapy, Intratumoural administration, Lung cancer, Antibody–photoabsorber conjugate

## Abstract

**Background:**

Photoimmunotherapy (PIT) is a potent modality for cancer treatment. The conventional PIT regimen involves the systemic delivery of an antibody–photoabsorber conjugate, followed by a 24-h waiting period to ensure adequate localisation on the target cells. Subsequent exposure to near-infrared (NIR) light selectively damages the target cells. We aimed to improve the efficacy of PIT *in vivo* by evaluating the effects of the different routes of conjugate administration on treatment outcomes.

**Methods:**

Subcutaneous Lewis lung carcinoma tumours were established in mice, targeting cluster of differentiation (CD)44 with an anti-CD44 antibody conjugated to IRDye700DX (IR700). The conjugate was administered via the intravenous or intratumoural route followed by the assessment of antibody binding and therapeutic effects of PIT.

**Findings:**

Compared to intravenous administration, intratumoural delivery of the CD44-IR700 conjugate significantly increased the number of cells binding to the conjugate by >five-fold. This method, combined with NIR light irradiation, halved tumour growth when compared to intravenous delivery. Reducing the interval between intratumoural injection and NIR light exposure to 30 min did not diminish efficacy, thereby demonstrating the feasibility of a 1-h procedure.

**Interpretation:**

Intratumoural administration of the antibody–photoabsorber conjugate enhanced the efficacy of PIT *in vivo*. A simplified, 1-h procedure involving conjugate tumour injection followed by irradiation emerged as a potent cancer treatment strategy.

**Funding:**

This study was supported by the 10.13039/501100001691Japan Society for the Promotion of Science, the 10.13039/100009619Japan Agency for Medical Research and Development, 10.13039/501100002241Japan Science and Technology Agency, and the 10.13039/501100008665Osaka Medical Research Foundation for Intractable Diseases.

**Research in context**

**Evidence before this study**

Near-infrared (NIR)-photoimmunotherapy (PIT) is a targeted cancer therapy that utilises an antibody–photoabsorber conjugate, activated by NIR light exposure. Following its systematic administration, this conjugate selectively accumulates in target cells over a period of approximately 24 h, with an advantage of minimal off-target effects owing to its selective cytotoxicity. Several studies have reported that targeted cells rapidly swell and burst within minutes upon light exposure, particularly demonstrating high cytotoxic efficiency *in vitro*. However, translating this efficacy from *in vitro* to *in vivo* models is challenging. Hence, strategies to enhance the therapeutic effects of NIR-PIT *in vivo* are essential and are actively being investigated.

**Added value of this study**

The results of this study establish that direct intratumoural injection of antibody–photoabsorber conjugates significantly enhances their binding to target cells, thereby considerably improving the efficacy of PIT for cancer treatment when compared with the conventional intravenous administration. Additionally, the therapeutic effect of PIT remained robust when exposed to NIR light for 30 min post intratumoural injection, suggesting a streamlined treatment protocol that can be completed in a single 1-h session.

**Implications of all the available evidence**

The findings of this study have demonstrated that antibody delivery is a key factor affecting the efficacy of NIR-PIT *in vivo*. Most antibodies that are commonly used in various animal studies and global clinical trials have a high molecular weight, which limits their tissue penetration efficiency. As NIR-PIT is a localised treatment that targets cells only at the site exposed to light, the traditional procedure of systemic administration of the conjugate cannot be considered as the most efficient antibody delivery method for PIT. It is crucial that the conjugate is delivered to the site of illumination, with other locations being irrelevant.

NIR-PIT can be performed through external or internal illumination. However, post-market surveillance in Japan revealed that in most cases, intratumoural illumination is employed, wherein a fibre optic is inserted directly into the tumour. The findings of this study suggest that combining intratumoural illumination with direct intratumoural conjugate delivery could enhance the therapeutic effects of NIR-PIT in clinical settings. This study opens new avenues for optimising cancer treatment strategies and promotes PIT as a highly effective modality for treating a broader spectrum of tumours worldwide.

## Introduction

Near-infrared (NIR)-photoimmunotherapy (PIT) has been recognised as a cutting-edge and effective approach for cancer treatment.^1^, ^2^, ^3^ This innovative treatment modality utilises monoclonal antibodies specific to cancer cell antigens, which are conjugated to a photoabsorptive agent, IRDye700DX (IR700). These conjugates selectively bind to antigenic sites on cancer cell surfaces. When illuminated with NIR light at approximately 690 nm, the bound conjugates elicit rapid and localised cellular destruction.^4^ The unique properties of the photoabsorber, which induce cytotoxicity only upon light exposure, combined with the targeted binding capacity of the antibodies, which attach only to target cells, allow NIR-PIT to exhibit exceptional selective cytotoxicity.

NIR-PIT has several advantages including its versatility, as it can be tailored to target various cancers—such as lung cancer,^5^ glioblastoma,^6^ melanoma,^7^ prostate cancer,^8^ pancreatic cancer,^9^ and triple-negative breast cancer^10^—by merely changing the type of antibody utilised. Moreover, the adaptability of NIR-PIT extends to the treatment of the tumour microenvironment, including regulatory T cells^11^^,^^12^ and cancer-associated fibroblasts,^13^ along with applications in managing conditions such as rheumatoid arthritis,^14^ candidiasis,^15^ and human immunodeficiency virus,^16^ as validated by animal studies. NIR-PIT exhibits remarkable efficacy *in vitro*, demonstrating the ability to impair almost all target cells. However, translating these promising results into *in vivo* applications has proven challenging, with some cases falling short of achieving sufficient efficacy. For instance, using NIR-PIT with cluster of differentiation (CD)44-IR700 alone has not yielded satisfactory outcomes *in vivo*, prompting investigations into combining it with programmed cell death ligand 1 (PD-L1) or cytotoxic T-lymphocyte associated antigen 4 (CTLA-4) antibodies to enhance its efficacy.^17^^,^^18^ Moreover, some animal studies have reported that NIR-PIT often fails to completely eliminate cancer, with frequent instances of tumour regrowth observed over time.^19^^,^^20^ In clinical trials, although there are reports of complete remission, instances of disease progression remain prevalent, highlighting the imperative need for developing strategies to enhance the efficacy of PIT *in vivo*.^3^^,^^21^

In this study, we aimed to address the limitations encountered in previous studies by using PIT against CD44—a recognised cancer stem cell surface antigen.^17^^,^^18^^,^^22^ We evaluated the proportion of tumour cells bound by IR700-conjugated anti-CD44 monoclonal antibody (CD44-IR700) following conventional intravenous administration and subsequently investigated whether direct intratumoural administration of CD44-IR700 enhances both the binding rate to tumour cells and the cytotoxic effect of PIT *in vivo*.

## Methods

### Reagents

The water-soluble silica-phthalocyanine derivative IR700 was provided by Rakuten Medical. Anti-mouse/human CD44-specific monoclonal antibody (clone IM7) was purchased from Bio X Cell (BE0039; Bio X Cell, RRID: AB_1107649). For flow cytometry, the following primary antibodies were used: PE/Cyanine7 anti-mouse CD3ε Antibody (100320, BioLegend, RRID: AB_312685), APC/Cyanine7 anti-mouse CD45 Antibody (103116, BioLegend, RRID: AB_312981), and PE anti-mouse CD8a antibody (12-0081-82, eBioscience, RRID: AB_465530). The secondary antibody used for flow cytometry was fluorescein isothiocyanate-labelled anti-rat immunoglobulin G2b monoclonal antibody (408205, BioLegend, AB_493001). For immunohistochemistry, the primary antibody was anti-mouse CD8a antibody (98941S, Cell Signalling Technology, RRID: AB_2756376). The secondary antibodies used for immunohistochemistry were peroxidase-labelled polymer-conjugated anti-rat IgG polyclonal antibody (Simple Stain MAX-PO [Rat], 414311, Nichirei Bioscience) and peroxidase-labelled polymer-conjugated anti-rabbit IgG polyclonal antibody (Simple Stain MAX-PO [R], 414341, Nichirei Bioscience, RRID: AB_2868561). Antibody validation is provided in the Supplementary File.

### Synthesis of IR700-conjugated anti-CD44 antibody

The IR700-conjugated anti-CD44 antibody was synthesised according to previously established protocols.^5^ Briefly, 1 mg of CD44 monoclonal antibody was initially dissolved in 1 mL of 1 × phosphate-buffered saline (PBS), followed by the addition of 100 μL of K_2_HPO_4_ buffer (pH = 9.0) and 52.2 μg of IR700. The mixture was incubated at room temperature for 2 h, after which it was subjected to ultra-centrifugal filter units (89892, Thermo Fisher Scientific) to eliminate any unbound IR700 molecules. The resulting IR700-conjugated anti-CD44 monoclonal antibody was denoted as CD44-IR700. The concentration of the antibody and the dye/protein ratio were determined using spectroscopy by measuring the absorbance of the conjugate at 280 and 689 nm, respectively. The extinction coefficients used were 216,000 M^−1^ cm^−1^ for CD44 monoclonal antibody at 280 nm and 165,000 M^−1^ cm^−1^ for IR700 at 689 nm. A correction factor of 0.095 was applied for IR700 at 280 nm. The purity of CD44-IR700 was assessed using sodium dodecyl sulphate polyacrylamide gel electrophoresis (SDS-PAGE) performed on a 4–12% gradient gel (NP0322BOX, Invitrogen). Fluorescence imaging of the gel was performed using a ChemiDoc touch imaging system (Bio-Rad), and the protein bands on the gel were stained with InstantBlue® Coomassie Protein Stain (ab119211, Abcam).

### Cell lines and cell culture

Lewis lung carcinoma (LLC) (JCRB1348, RRID: CVCL_5653),^23^^,^^24^ LLC-luc (JCRB1716, RRID: CVCL_JG92),^25^ which stably express luciferase, and MKN74 cells (JCRB 0255, RRID: CVCL_2791) were procured from the Japanese Cancer Research Resources Bank. LLC, LLC-luc cells were cultured in Dulbecco's modified Eagle medium (DMEM), supplemented with 10% foetal bovine serum (FBS) and 1% penicillin/streptomycin. MKN74 cells were cultured in RPMI 1640 medium, supplemented with 10% foetal bovine serum (FBS) and 1% penicillin/streptomycin. Cultures were maintained at 37 °C in a humidified atmosphere containing 5% CO_2_. All human cell lines were validated by STR profiling. Details regarding cell line validation are provided in the Supplementary File. Mycoplasma testing confirmed that all cell lines tested negative.

### Ethics

The protocols for animal experiments were approved by the Institutional Animal Care and Use Committee of Osaka University (Approval no.: 30-092-010), ensuring compliance with relevant ethical regulations, including the ARRIVE guidelines. Six-week-old C57/BL6JJcl mice or BALB/cAjcl-*nu/nu* mice were obtained from the Central Laboratory for Experimental Animals, Japan and housed in pathogen-free conditions, with up to five mice per cage. This study used female mice, consistent with previous reports that highlight the limited therapeutic efficacy of CD44-targeted PIT.^17^^,^^18^ In experiments using LLC cells, C57/BL6JJcl mice were used, and in experiments using MKN74 cells, BALB/cAjcl-*nu/nu* mice were used. Prior to the administration of CD44-IR700 or PBS, C57/BL6JJcl mice were shaved at the sites of subcutaneous tumour transplantation. For the procedures performed on mice, anaesthesia was induced via intraperitoneal injection of a triple-combination anaesthetic consisting of 0.75 mg/kg medetomidine, 4 mg/kg midazolam, and 5 mg/kg butorphanol.^26^ Tumour volume was calculated using the formula: (major axis × minor axis^2^) × 0.5. The tumour volume ratio was shown as a comparison to the tumour size on day 0, which was set as 1. Mice were sacrificed if they exhibited difficulty in feeding or drinking, impaired mobility, or when the tumour volume exceeded 2500 mm³.^27^^,^^28^ Euthanasia was conducted using carbon dioxide.

### *In vitro* CD44 expression analysis

To evaluate CD44 expression on LLC and MKN74 cells, 4 × 10^5^ cells were harvested in PBS and then incubated with CD44-IR700 (10 μg/mL) for 20 min. After washing with PBS, the cells were analysed using a BD FACSymphony A3 flow cytometer (BD Biosciences) and the FlowJo software (BD Biosciences). In the blocking assay, cells were pre-incubated with a solution of 1 mg/mL unconjugated anti-CD44 antibody.

### *In vitro* NIR-PIT and phototoxicity assay

LLC, LLC-luc, or MKN74 cells (1 × 10^5^) were seeded into 96-well plates 12 h prior to the experiments. The culture medium was replaced with fresh medium containing 10 μg/mL of CD44-IR700 and incubated at 37 °C for 3 h. After washing with PBS, phenol red-free medium was added. Subsequently, the cells were irradiated with an NIR light-emitting diode (L690-66-60-550, Ushio), emitting light at a wavelength of 670–710 nm. To assess the viability of LLC cells, 1 h after NIR irradiation, the cell counting kit-8 reagent (CK04, Dojindo) was added to each well and incubated at 37 °C for 1 h. The absorbance of the cell medium was measured at 450 nm using a Spectramax i3X microplate reader (Molecular Devices). For LLC-luc cells, luciferin solution (ONE-Glo™ EX Luciferase Assay System, E8110, Promega) was added 1 h after NIR irradiation, as per the manufacturer's instructions, and luciferase activity was measured on a Spectramax i3X. The actual power density (mW/cm^2^) of the NIR light-emitting diode was measured using an optical power metre (PMKIT-15-01, Newport).

### Assessment of CD44-IR700 binding to target cells

To quantify CD44-IR700 binding to target cells *in vitro*, 4 × 10^5^ LLC-luc cells were incubated with CD44-IR700 (10 μg/mL) for 20 min. After washing, the cells were stained with anti-rat IgG2b monoclonal antibody and analysed using a BD FACSCanto II Cytometer (BD Biosciences) and the FlowJo software.

To quantify CD44-IR700 binding to target cells in allograft tumours, 1 × 10^7^ LLC-luc cells were injected subcutaneously into the right dorsum of the mice. Three weeks after injection, mice were randomised into three groups: (1) no treatment (Ctrl); (2) intravenous injection (IV) of 100 μg of CD44-IR700; and (3) intratumoural injection (IT) of 100 μg of CD44-IR700. The following day, tumours were harvested, shredded, and incubated in the collagenase-containing buffer: collagenase type IV (100 U/mL; 17104019, Invitrogen), deoxyribonuclease I (50 μg/mL; 10104-159001, Roche), and 10% FBS in DMEM for 45 min. After incubation, cells were treated with ACK lysing buffer (A1049201, Thermo Fisher Scientific) and passed through a 100-μm cell strainer to remove debris. Following centrifugation, the cell pellet was dissolved in 2% FBS in PBS and analysed by flow cytometry. Isolated cells were stained with the LIVE/DEAD Fixable Dead Cell Stain Kit (L34966, Invitrogen) to exclude dead cells, followed by incubation with Fc-blocking antibody (BioLegend) and staining with anti-rat IgG2b monoclonal antibody. The results were analysed using a BD FACSCanto II Cytometer (BD Biosciences) and the FlowJo software.

### *Ex vivo* fluorescence imaging

LLC cells (1 × 10^7^) were injected subcutaneously into the right dorsum of the mice. Eleven days post-injection, when the tumour size reached approximately 100 mm³, the mice were randomly divided into three groups: (1) no injection (Ctrl); (2) intravenous injection of 50 μg/50 μL CD44-IR700 (IV); and (3) intratumoural injection of 50 μg/50 μL CD44-IR700 (IT). The next day, the mice were sacrificed, and the tumour, liver, spleen, kidney, heart, and lungs were excised and measured using the IVIS Lumina II (Revvity). Near-infrared fluorescence images were acquired with a 1-s exposure time, using 640 nm excitation and autofluorescence correction at 535 nm, both measured using the Cy5.5 filter. The fluorescence intensities of the IV and IT groups were expressed relative to the average fluorescence intensity of the Ctrl group (radiant efficiency ratio).

### *In vivo* NIR-PIT experiments

For the comparison between intravenous and intratumoural administration, 1 × 10^7^ LLC cells or 1 × 10^7^ MKN74 cells were injected subcutaneously into the right dorsum of the mice. Eleven days post-injection, when tumour size reached approximately 100 mm^3^, mice were randomised into three groups: (1) no injection (Ctrl); (2) IV of 50 μg/50 μL CD44-IR700 (CD44-IR700_IV); and (3) IT of 50 μg/50 μL CD44-IR700 (IT group). Tumours were irradiated with a NIR light laser (BrixX690 PDT, Omicron) at 50 J/cm^2^ on the following day, followed by 100 J/cm^2^ on the subsequent day.

To ensure that the effects of intratumoural administration were not due to nonspecific mechanisms, additional experiments were conducted. First, to confirm that the observed effects were not caused by CD44-IR700 alone without NIR light exposure, an experiment was conducted by omitting the NIR light irradiation from the above-mentioned protocol. Next, since intratumoural administration might bypass the need for antibody binding, we conducted an experiment wherein unconjugated free-IR700 was administered instead of CD44-IR700, following the same protocol. The free-IR700 dose was adjusted to match the molar equivalent of IR700 in the CD44-IR700 treatments, with 1.95 μg of free-IR700 administered per treatment. Finally, we conducted an experiment to exclude the potential impact of liquid injection into the tumour by comparing the effects of intravenous and intratumoural administration. For this experiment, 1 × 10^7^ LLC cells were injected subcutaneously into the right dorsum of the mice. Eleven days post-injection, when tumour size reached almost 100 mm^3^, mice were randomised into three groups: (1) IV administration of 50 μg/50 μL CD44-IR700 and intratumoural administration of 50 μL of PBS (CD44-IR700_IV); (2) IV administration of 50 μL of PBS and IT of 50 μg of CD44-IR700 (CD44-IR700_IT). Tumours were irradiated with a NIR light laser at 50 J/cm^2^ on the following day, followed by 100 J/cm^2^ on the subsequent day.

To compare NIR irradiation 24 h after intravenous administration with NIR irradiation 30 min after intratumoural administration, 1 × 10^7^ LLC cells were subcutaneously injected into the right dorsum of the mice. Eleven days post-injection, when tumour size reached approximately 100 mm^2^, mice were randomised into three groups: (1) IV of 50 μg/50 μL CD44-IR700 and intratumoural administration of 50 μL of PBS followed by NIR light irradiation after 24 h (CD44-IR700_IV_24h); (2) intravenous administration of 50 μL of PBS and IT of 50 μg of CD44-IR700 followed by NIR light irradiation after 24 h (CD44-IR700_IT_24h); and (3) intravenous administration of 50 μL of PBS and IT of 50 μg of CD44-IR700 followed by NIR light irradiation after 30 min (CD44-IR700_IT_30m).

### Flow cytometry analysis of tumour-infiltrating CD8-positive T cells

Seven days after photoimmunotherapy, tumours were harvested, shredded, and incubated in a collagenase-containing buffer: collagenase type IV (100 U/mL; 17104019, Invitrogen), deoxyribonuclease I (50 μg/mL; 10104-159001, Roche), and 10% FBS in DMEM for 45 min. After incubation, the cells were treated with ACK lysing buffer (A1049201, Thermo Fisher Scientific) and passed through a 100-μm cell strainer to remove debris. Following centrifugation, the cell pellet was resuspended in 2% FBS in PBS and analysed by flow cytometry. The isolated cells were stained with the LIVE/DEAD Fixable Dead Cell Stain Kit (L34966, Invitrogen) to exclude dead cells, followed by incubation with Fc-blocking antibody (BioLegend) and stained with anti-CD45, -CD3, and -CD8 antibodies. The results were analysed using a BD FACSCanto II Cytometer (BD Biosciences) and the FlowJo software. The data represents the percentage of CD8-positive T cells among the live cells within the tumour.

### Quantitative real-time PCR

Tumours were harvested, and total RNA was extracted using the FastGene RNA Premium Kit (FG-81250, NIPPON Genetics). cDNA was synthesised using the PrimeScript™ RT reagent Kit with gDNA Eraser (RR047A, Takara Bio). Quantitative PCR reactions were performed using the TaqMan Fast Advanced Master Mix (4444965, Applied Biosystems) and run on a QuantStudio 7 system (Applied Biosystems). The following primers were used: interferon-γ (*Ifng*; Mm01168134_m1, Thermo Fisher Scientific), TNF-α (*Tnf*; Mm00443258_m1, Thermo Fisher Scientific), and the endogenous control gene β-actin (*Actb*; Mm02619580_g1, Thermo Fisher Scientific). Gene expression data were normalised to the expression of *Actb*.

### Immunohistochemistry and haematoxylin-eosin staining

Evaluation of CD44-IR700 binding to tumours: LLC cells (1 × 10^7^) were subcutaneously injected into the right dorsum of the mice. Eleven days post-injection, CD44-IR700 was either not administered, administered intravenously, or intratumourally. After 24 h, perfusion fixation with 4% paraformaldehyde was conducted. Tumours were harvested and embedded in Tissue-Tek O.C.T. Compound (Sakura) by flash freezing. Frozen tissue blocks were sectioned using a cryostat and mounted onto glass slides. Endogenous peroxidase activity was blocked using 0.3% hydrogen peroxide in methyl alcohol for 30 min. After rinsing in Tris-buffered saline with Tween 20, sections were treated with peroxidase-labelled polymer-conjugated anti-rat IgG polyclonal antibody. Development was carried out using the DAB Substrate kit, and sections were counterstained with haematoxylin before observation under a microscope.

Evaluation of CD8-positive T cells after photoimmunotherapy: Seven days after photoimmunotherapy, tumours were harvested and fixed in formalin. Paraffin sections were prepared, and endogenous peroxidase activity was blocked with 0.3% hydrogen peroxide in methanol for 30 min. After rinsing in Tris-buffered saline with Tween 20, sections were stained with CD8 antibody at 4 °C overnight and treated with peroxidase-labelled polymer-conjugated anti-rat IgG polyclonal antibody. DAB staining and haematoxylin counterstaining were performed before observing under the microscope. The number of infiltrating CD8^+^ cells per a view in tumour tissue was counted using the Hybrid Cell Count (KEYENCE), as previously reported.^29^

### Blood tests

Blood samples were collected from mice 7 days after photoimmunotherapy. The analyses were conducted by Oriental Yeast Co., and haemoglobin, AST, and BUN levels were measured.

### Statistics

Data are expressed as mean + standard error of the mean of a minimum of three experiments unless otherwise indicated. Statistical analyses were performed using the Prism software (GraphPad). For two-group comparisons, the Mann–Whitney U test was used. For comparisons among three groups, the Kruskal–Wallis test was applied, followed by Dunn's test adjusted with Bonferroni correction to determine which groups significantly differed from the others. Statistical significance was set at *p* < 0.05.

### Role of funders

The funders of this study had no role at all in the study design, data collection, data analyses, interpretation, or writing of this report.

## Results

### Synthesis of CD44-IR700 and *in vitro* therapeutic effects

**Fig. 1:**
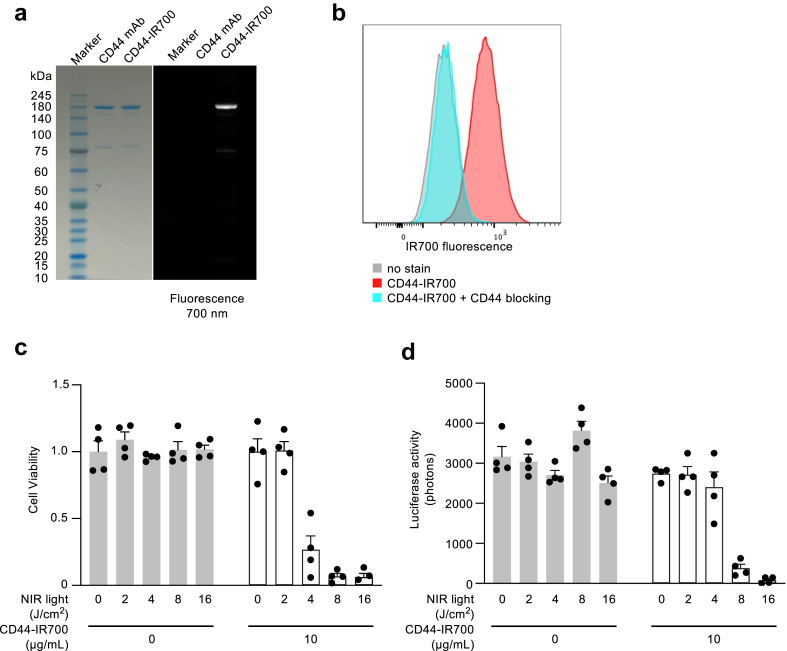
**CD44-IR700 conjugate generation and *in vitro* effects of NIR light photoimmunotherapy using CD44-IR700**. (a) Generation of the CD44-IR700 conjugate as confirmed by sodium dodecyl sulphate polyacrylamide gel electrophoresis. The left panel indicates Coomassie blue staining for protein detection and the right panel displays fluorescence at 700 nm. (b) Flow cytometry analysis demonstrating CD44-IR700 binding to Lewis lung carcinoma (LLC) cells. The panel shows LLC cells after incubation with CD44-IR700. In the blocking experiment, cells were pre-incubated with an excess amount of unconjugated anti-CD44 antibody. (c) Assessment of cell viability using a colourimetric assay following photoimmunotherapy with CD44-IR700 in LLC cells. Cells were cultured in a medium with or without CD44-IR700 and subsequently exposed to NIR light. Viability was measured and normalised against cells grown in a medium without CD44-IR700 and not exposed to NIR light (n = 4 for each bar). (d) Evaluation of luciferase activity post-photoimmunotherapy in LLC-luc cells treated with CD44-IR700. LLC-luc cells were cultured with or without CD44-IR700 and subjected to NIR light exposure, after which luciferase activity was quantified (n = 4 for each bar). CD44-IR700, IR700-conjugated anti-CD44 monoclonal antibody; CD, cluster of differentiation; and NIR, near-infrared.

The CD44-IR700 conjugate was successfully synthesised using the anti-CD44 monoclonal antibody, and its integrity was confirmed by observing a strong binding affinity between the antibody and IR700. This binding affinity was absent when the anti-CD44 antibody was used alone, as indicated by the lack of fluorescence signals on SDS-PAGE (Fig. 1a). Validation of the specific interaction of CD44-IR700 with LLC cells was achieved through flow cytometry analysis (Fig. 1b). Furthermore, specificity was demonstrated through blocking assays with an excess of CD44 monoclonal antibodies, highlighting the antibody-dependent nature of CD44-IR700 binding.

The efficacy of *in vitro* PIT was assessed using the synthesised CD44-IR700 conjugate (Fig. 1c and d). Notably, NIR light irradiation of LLC cells in the absence of the CD44-IR700 conjugate did not induce significant cytotoxicity. In contrast, upon binding of CD44-IR700 to the cells followed by NIR light exposure, a light dose-dependent cytotoxic effect was observed. Remarkably, PIT resulted in the impairment of over 90% of the cells *in vitro*.

### Intratumoural administration enhanced the delivery of CD44-IR700 to tumours

In an attempt to translate the clear *in vitro* effects into an *in vivo* context, we recognised the simplicity of the PIT mechanism: an IR700-conjugated antibody binds to target cells and induces cell damage upon light exposure. Thus, we hypothesised that efficient antibody delivery is critical for *in vivo* efficacy.

**Fig. 2:**
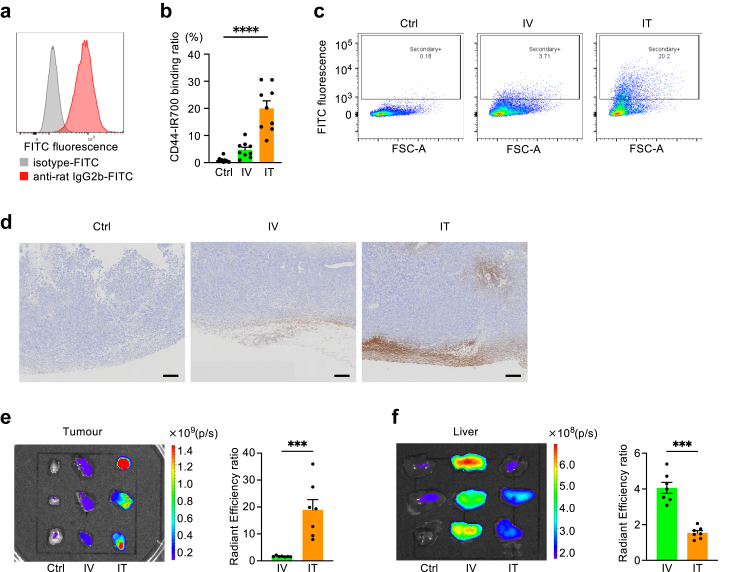
**Intratumoural administration enhances the delivery of CD44-IR700 to tumours**. (a) Flow cytometry analysis demonstrating CD44-IR700 binding to Lewis lung carcinoma cells *in vitro*. The cells were visualised using a secondary antibody against CD44-IR700 (anti-rat IgG2b-FITC). (b) Quantification of CD44-IR700 binding to allograft tumour cells for each treatment group: Ctrl, IV (100 μg), and IT (100 μg) CD44-IR700 administration (n = 9 per group). CD44-IR700 binding to isolated tumour cells was detected using the secondary antibody. (c) Representative flow cytometry images from figure (b) illustrating the binding of CD44-IR700 to cells in the allograft tumour. Treatments were administered as described in (b). (d) Representative immunohistochemical staining of CD44-IR700 at 40 × magnification. Subcutaneous tumours were induced in mice, followed by treatment in each group: Ctrl, IV (50 μg), and IT (50 μg) CD44-IR700 administration. CD44-IR700 was visualised using a secondary antibody. Scale bar, 200 μm. (e and f) Biodistribution of CD44-IR700 in tumour (e) and liver (f), visualised using IVIS imaging system the day after administration of CD44-IR700. Treatments were administered as described in (d). The bar graphs on the right shows the fluorescence values of the intravenous and intratumoural administration groups compared to the Ctrl group (n = 7 per group). Data are presented as mean + standard error of the mean. Statistical difference was evaluated using Mann–Whitney U tests for comparisons between two groups and the Kruskal–Wallis test for comparisons between three groups, with asterisks denoting the levels of significance (∗∗∗*p* < 0.001 and ∗∗∗∗*p* < 0.0001). CD44-IR700, IR700-conjugated anti-CD44 monoclonal antibody; FITC, fluorescein isothiocyanate; IgG, immunoglobulin G; FSC-A, forward scatter area; Ctrl, control; IV, intravenous; and IT, intratumoural.

By utilising a secondary antibody against anti-CD44, we detected the binding of CD44-IR700 to the target cells. Initially, we confirmed the detection of the LLC-luc cell line bound with CD44-IR700 using the secondary antibody *in vitro* (Fig. 2a). Subsequently, we established subcutaneous tumours in mice using the same cell line, followed by the conventional intravenous administration of CD44-IR700. The tumours were harvested and minced into single cells the following day, and the binding rate of CD44-IR700 to tumour cells was assessed. We observed that intravenously administered CD44-IR700 bound to only 3.7% of the cells on average in the allograft tumour (Fig. 2b and c).

To enhance binding efficiency, we directly injected CD44-IR700 into the tumour mass. The following day, the binding to CD44-positive cells had increased to 20% on average (Fig. 2b), representing a 5.4-fold increase when compared to intravenous administration. Immunostaining also revealed a greater distribution of CD44-IR700 within tumours after intratumoural administration when compared with that of intravenous administration (Fig. 2d).

Then, we evaluated the biodistribution of CD44-IR700 in both tumour and normal tissues by directly observing IR700 fluorescence. As a result, intratumoural administration significantly increased IR700 accumulation in the tumour while decreasing its presence in the liver and other normal organs when compared to intravenous administration, as evaluated using Mann–Whitney U test (Fig. 2e and f and Supplementary Figure S1). In summary, intratumoural administration significantly enhanced the delivery of CD44-IR700 to target tumours and reduced non-specific accumulation in distant organs.

### Improved *in vivo* PIT effect by intratumoural administration of CD44-IR700

**Fig. 3:**
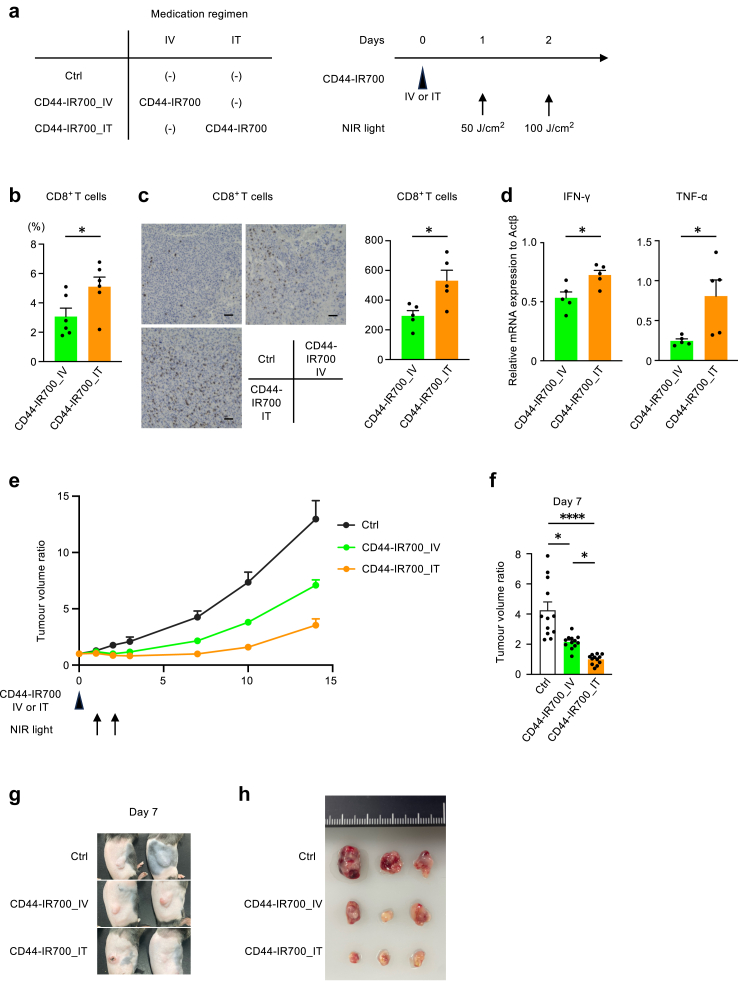
**Intratumoural delivery of CD44-IR700 enhanced efficacy of photoimmunotherapy *in vivo***. (a) Schematic of experimental schedule. Mice bearing subcutaneous Lewis lung carcinoma tumours were treated with either no CD44-IR700 (Ctrl), CD44-IR700 via IV injection (CD44-IR700_IV), or CD44-IR700 via IT injection (CD44-IR700_IT), followed by NIR light irradiation on subsequent days. (b) The ratio of tumour-infiltrating CD8-positive T cells in live cells, as determined by flow cytometry on day 7 post-treatment, from the CD44-IR700_IV and CD44-IR700_IT groups (n = 6 per group). (c) Immunohistochemical staining of tumours 7 days after photoimmunotherapy. CD8-positive T cells were stained. The scale bar represents 50 μm. The numbers of CD8-positive T cells per view field were counted (n = 5 mice for each group). (d) Cytokine expression in tumours, as determined by quantitative PCR analysis on day 3 post-treatment with photoimmunotherapy, from the CD44-IR700_IV and CD44-IR700_IT groups (n = 5 per group). (e) Tumour volume monitoring. Tumour size was measured starting from the day of CD44-IR700 administration (day 0), and the change in size was expressed as a ratio relative to the initial volume on day 0 (n = 12 per group). (f) Tumour volume ratio on day 7. The graph shows the fold increase in tumour volume on day 7 compared with that on day 0 (n = 12 per group). (g) Representative images displaying tumours from each treatment group on day 7 post-administration. (h) Representative images of tumours excised on day 7 post-treatment from each therapeutic group. Data are presented as mean + standard error of the mean. Statistical difference was assessed using Mann–Whitney U tests, with significance denoted by asterisks (∗*p* < 0.05 and ∗∗∗∗*p* < 0.0001). CD44-IR700, IR700-conjugated anti-CD44 monoclonal antibody; CD, cluster of differentiation; NIR, near-infrared; Ctrl, control; IV, intravenous; IT, intratumoural; Ifnγ, interferon-γ; and Tnf, tumour necrosis factor-alpha.

We subsequently evaluated the therapeutic impact of intratumoural delivery *in vivo*. Mice with subcutaneous tumours established by LLC cell inoculation were randomised into three groups on day 0: a Ctrl group that did not receive CD44-IR700, an intravenous CD44-IR700 administration group (CD44-IR700_IV), and an intratumoural CD44-IR700 administration group (CD44-IR700_IT) (Fig. 3a). All mice underwent percutaneous exposure to NIR light on days 1 and 2.

First, we compared tumour microenvironment between the CD44-IR700_IV and CD44-IR700_IT groups. Flow cytometry revealed a higher infiltration of cytotoxic CD8-positive T cells in the CD44-IR700_IT group when compared to the CD44-IR700_IV group (Fig. 3b). Immunostaining showed consistent results (Fig. 3c). Additionally, qPCR analysis demonstrated increased expression of interferon-γ and tumour necrosis factor-alpha (TNFα) in the tumours of the CD44-IR700_IT group when compared to the CD44-IR700_IV group (Fig. 3d). These findings indicate that switching the administration route of CD44-IR700 from intravenous to intratumoural elicited a stronger cellular immune response in the tumour.

Next, we investigated the tumour suppressing effects. The data showed a significant reduction in tumour volume in the CD44-IR700_IV group when compared to the Ctrl group (Fig. 3e–h). Remarkably, the CD44-IR700_IT group exhibited a smaller tumour volume than the CD44-IR700_IV group. Seven days following the administration of CD44-IR700, the tumour volume ratio relative to day 0 was recorded as 4.3 for the Ctrl group, 2.2 for the CD44-IR700_IV group, and 1.0 for the CD44-IR700_IT group, with statistically significant differences as determined using the Kruskal–Wallis test, followed by Dunn's test adjusted with Bonferroni correction (Fig. 3f).

To confirm that this outcome was not due to nonspecific effects, we conducted additional experiments. First, we ruled out the possibility that intratumoural fluid administration caused physical damage to the tumour (Supplementary Figure S2a). In this experiment, the CD44-IR700_IV group received an intravenous dose of CD44-IR700 and an intratumoural dose of PBS, while the CD44-IR700_IT group received an intratumoural injection of CD44-IR700 and intravenous dose of PBS. The results, analysed using the Mann–Whitney U test, demonstrated a significant reduction in tumour size in the CD44-IR700_IT group when compared to the CD44-IR700_IV group (Supplementary Figure S2b and c), indicating that the improved efficiency of intratumoural administration was not due to physical damage to the tumour.

Next, we confirmed that administering free-IR700, without the antibody, had no tumour-suppressing effects, whether it was delivered via the intravenous or intratumoural route (Supplementary Figure S3). Additionally, no tumour suppression was observed when CD44-IR700 was administered via the intravenous or intratumoural route without subsequent light exposure (Supplementary Figure S4). Finally, we conducted experiments using the CD44-negative MKN74 cell line.^30^^,^^31^ As shown in Supplementary Figure S5a, CD44-IR700 did not bind to MKN74 cells. CD44-IR700 did not exhibit any tumour-suppressing effects against MKN74 cells *in vitro* (Supplementary Figure S5b). Additionally, we established a subcutaneous tumour model using MKN74 cells and observed that their tumour growth rate was relatively slow, as previously reported.^30^^,^^32^ Importantly, even after light exposure following intravenous or intratumoural administration of CD44-IR700, no tumour-suppressing effects were observed against MKN74 cells *in vivo* (Supplementary Figure S5c–e). We also attempted CD44 knockdown in LLC cells; however, as previously reported,^33^^,^^34^ these cells did not form tumours effectively and could not be used in the experiment. These results demonstrate that the tumour-suppressing effects observed in Fig. 3 for CD44-IR700_IT are due to the cytotoxic effects of CD44-IR700 bound to CD44, activated by NIR light exposure.

Next, we evaluated the safety profile of CD44-IR700. Histological analysis of the liver, heart, kidneys, and lungs showed no differences between the control group (untreated), CD44-IR700 IV, and CD44-IR700 IT administration groups (Supplementary Figure S6a). Furthermore, no abnormalities in haemoglobin levels, AST (liver function), or BUN (kidney function) were detected during blood chemical analysis (Supplementary Figure S6b). Therefore, within the scope of this screening, no side effects were detected following either intravenous or intratumoural administration of CD44-IR700.

### Efficacy of intravenous administration followed by NIR light irradiation within 1 h

In our previous experiments, we adhered to the established PIT protocol, which typically involves a waiting period of over 24 h between the administration of CD44-IR700 and subsequent NIR light exposure. This waiting period is primarily intended to facilitate the systemic circulation of the injected antibody, allowing it to accumulate on the target cells throughout the body. However, we hypothesised that this waiting period might be unnecessary for intratumoural administration, wherein the systemic circulation of the antibody is not a prerequisite. Consequently, we sought to investigate the feasibility of conducting NIR light irradiation immediately after intratumoural delivery of the antibody, thereby potentially simplifying the process into a single continuous treatment.

**Fig. 4:**
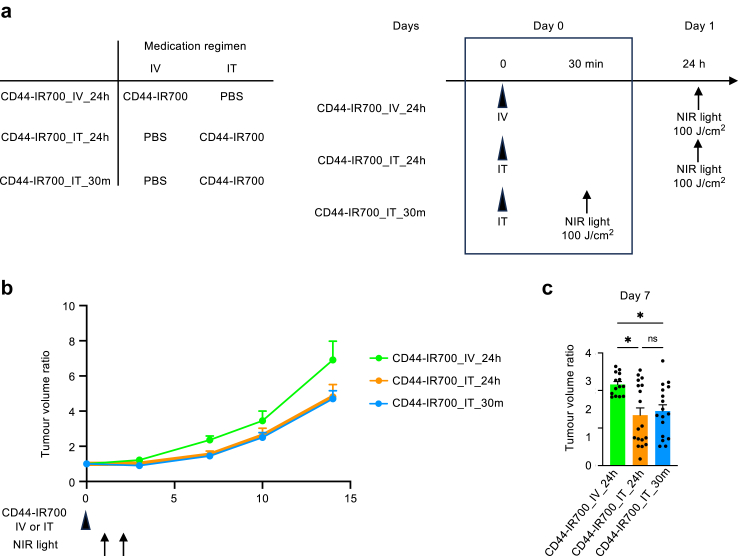
**The interval between intratumoural administration of CD44-IR700 and NIR irradiation, whether 30 min or 24 h, yields the same effect**. (a) Schematic of experimental schedule. Lewis Lung carcinoma cell-induced subcutaneous tumours were established in mice. The mice were divided into three groups for the following treatments: CD44-IR700_IV_24h: IV administration of CD44-IR700 and IT of PBS, with NIR light irradiation 24 h after administration; CD44-IR700_IT_24h: IT administration of CD44-IR700 and IV injection of PBS, followed by NIR light irradiation 24 h later; and CD44-IR700_IT_30m: IT administration of CD44-IR700 and IV injection of PBS, with NIR light irradiation conducted 30 min post-administration. (b) Tumour volume measurement. The tumour volume was monitored from the day of CD44-IR700 administration (designated as day 0), and the change in tumour size was presented as a ratio to the volume on the day of administration (n = 14–18 per group). (c) Tumour volume ratio on day 7 relative to day 0 (n = 14–18 per group). Data are expressed as mean + standard error of the mean. Statistical difference was assessed using Kruskal–Wallis test, with asterisks denoting the levels of significance (∗*p* < 0.05). ‘ns’ indicates a lack of statistical significance. CD44-IR700, IR700-conjugated anti-CD44 monoclonal antibody; PBS, phosphate-buffered saline; CD, cluster of differentiation; NIR, near-infrared; Ctrl, control; IV, intravenous; and IT, intratumoural.

Using LLC cells, we established subcutaneous tumours in mice, which were then divided into three groups on day 0: the CD44-IR700_IV_24h group received an intravenous injection of CD44-IR700 and intratumoural PBS, with NIR light irradiation conducted after 24 h; in the CD44-IR700_IT_24h group, CD44-IR700 was injected via the intratumoural route, PBS intravenously, and NIR light irradiation was performed after 24 h; finally, the CD44-IR700_IT_30m group received an intratumoural injection of CD44-IR700, intravenous PBS, and NIR light irradiation after 30 min (Fig. 4a). Consistently across all experiments, intratumoural administration of CD44-IR700 proved to be more effective in suppressing tumour growth when compared to intravenous delivery. Notably, the timing of NIR light exposure, whether at 30 min or 24 h post-administration, did not influence the therapeutic outcome (Fig. 4b and c). These results suggest that intratumoural administration followed by NIR light exposure can be implemented as a sequential therapy, offering the potential to complete the entire treatment within one hour.

## Discussion

In this study, we demonstrated the efficacy of direct intratumoural injection of antibody–photoabsorber conjugates in enhancing their association with target cells, thereby increasing the efficacy of PIT against tumours. Importantly, this intratumoural delivery approach bypasses the 24-h systemic circulation period required for the conjugates to reach target cells, without compromising therapeutic efficacy.

Conventional cancer drug therapies are typically administered systemically, which is essential for treating metastases or circulating tumour cells. However, systemic administration often results in low drug concentrations at tumour sites and can lead to systemic side effects.^35^^,^^36^ In contrast, PIT is a localised treatment focused on controlling cancer cells within a specific area illuminated by NIR light. Thus, it is sufficient for the conjugate to be localised within the area exposed to light. Although most conventional PIT experiments and clinical trials have utilised full antibodies,^1^^,^^3^^,^^21^^,^^37^ their large molecular size can result in significant variations in tissue and plasma concentrations.^38^, ^39^, ^40^ Our study has shown that the local administration of CD44-IR700 increases the proportion of tumour cells bound by the conjugate and inhibits tumour growth upon NIR light irradiation. Previous reports indicate that intratumoural administration can improve the therapeutic effects of immunocytokines and antibodies when compared to systemic administration.^41^, ^42^, ^43^, ^44^ The present study suggested that a similar approach could be applied to PIT. In summary, to enhance the efficacy of PIT *in vivo*, increasing the delivery of conjugates to target cells is advantageous, with intratumoural administration being a feasible method to achieve this.

We propose that intratumoural administration of conjugates is feasible in clinical settings, although its applicability may be lesser than that of intravenous administration. The most suitable cases for intratumoural administration are those currently receiving intratumoural irradiation as part of PIT.^45^, ^46^, ^47^, ^48^ For instance, in a post-marketing surveillance study of NIR-PIT using cetuximab-IR700 (Alluminox™) in Japan, intratumoural NIR irradiation with a cylindrical diffuser was performed in 54 out of 61 cases following systemic administration of the conjugate. Specifically, the procedure involves placing a needle catheter within the tumour instead of the direct insertion of the cylindrical diffuser. The fibre optic is subsequently inserted into the catheter for illumination. If the conjugate is injected concurrently with catheter placement and the fibre is retained after the inner needle is removed, a high concentration of the conjugate can be ensured at the illuminated site. Even in cases where a frontal diffuser is employed for NIR light irradiation from outside the tumour, intratumoural administration remains feasible when the tumour is visually or endoscopically accessible. If a 24-h interval between intratumoural administration and NIR light irradiation is necessary, patients would be required to undergo invasive procedures for 2 consecutive days. However, our experimental evidence suggests that a 30-min interval may be sufficient. It is conceivable that completing both intratumoural administration and NIR light irradiation within an hour would be ideal for humans.

This study has certain limitations that warrant acknowledgement. Firstly, the quantification of the binding affinity of CD44-IR700 to cells was conducted using minced tumour samples, as illustrated in Fig. 2b and c. During this process, there is a possibility that some CD44-IR700 bound to tumour cells *in vivo* may have been dislodged, potentially leading to an underestimation of the antibody binding rate. However, as shown in Fig. 2b–d, consistent results were observed with higher antibody binding rates in intratumoural administration via immunostaining and IR700 fluorescence. Secondly, although this study confirmed that targeting CD44 in photoimmunotherapy results in increased tumour distribution and enhanced antitumour effects with intratumoural administration, it is possible that if a more tumour-specific target had been used, the difference between intravenous and intratumoural administration might have been smaller. Thirdly, although we did not combine intratumoural and intravenous administration in this study, it is possible that combining both methods could have led to a more even distribution of the antibody and an additive therapeutic effect. Lastly, this study primarily utilised LLC cells for experiments involving subcutaneous tumours in mice. Since LLC tumours may be relatively soft, further investigation is necessary to determine whether the improvement in drug distribution with intratumoural administration can be applied similarly to more solid tumours.

In conclusion, our study highlights the potential of intratumoural administration of antibody–photoabsorber conjugates *in vivo* to increase their binding rate to target cells when compared to conventional therapy. This underscores the significance of intratumoural administration in the context of PIT, offering promising avenues for enhancing its efficacy.

## Contributors

**Yuichi Adachi:** Writing – original draft, Visualisation, Validation, Resources, Project administration, Methodology, Investigation, Funding acquisition, Formal analysis, Accessing and verifying the underlying data, Data curation, and Conceptualisation. **Kotaro Miyake:** Writing – review & editing, Writing – original draft, Visualisation, Supervision, Software, Resources, Project administration, Methodology, Investigation, Funding acquisition, Formal analysis, Accessing and verifying the underlying data, Data curation, and Conceptualisation. **Kika Ohira:** Resources, Methodology, and Investigation. **Shingo Satoh:** Resources, Methodology, Funding acquisition, and Investigation. **Kentaro Masuhiro:** Resources, Methodology, and Investigation. **Ryuya Edahiro:** Resources and Methodology. **Yuya Shirai:** Resources and Methodology. **Maiko Naito:** Resources and Methodology. **Yujiro Naito:** Resources and Methodology. **Takayuki Shiroyama:** Methodology. **Shohei Koyama:** Supervision, Resources, Project administration, and Methodology. **Haruhiko Hirata:** Methodology. **Kota Iwahori:** Methodology. **Izumi Nagatomo:** Writing – review & editing, Writing – original draft, Visualisation, Supervision, Resources, Project administration, Methodology, Funding acquisition, and Conceptualisation. **Yoshito Takeda:** Writing – review & editing, Supervision, Resources, Project administration, and Methodology. **Atsushi Kumanogoh:** Writing – review & editing, Writing – original draft, Visualisation, Supervision, Resources, Project administration, Methodology, Funding acquisition, and Conceptualisation. All authors read and approved the final version of the manuscript.

## Data sharing statement

Detailed data for each experiment can be provided upon request by contacting the corresponding authors: kotaromiyake@imed3.med.osaka-u.ac.jp; kumanogo@imed3.med.osaka-u.ac.jp.

## Declaration of interests

The authors declare that they have no known competing financial interests or personal relationships that could have appeared to influence the work reported in this paper.

## Appendix ASupplementary data

Supplementary Figures

Supplementary Figure Caption
