# Supplementary material for: Enhancing the efficacy of near-infrared photoimmunotherapy through intratumoural delivery of CD44–targeting antibody–photoabsorber conjugates
Source: eBioMedicine. 2025 Jan 22;112:105566. doi: 10.1016/j.ebiom.2025.105566 (PMC11795636; doi:10.1016/j.ebiom.2025.105566)

Supplementary Figure S1

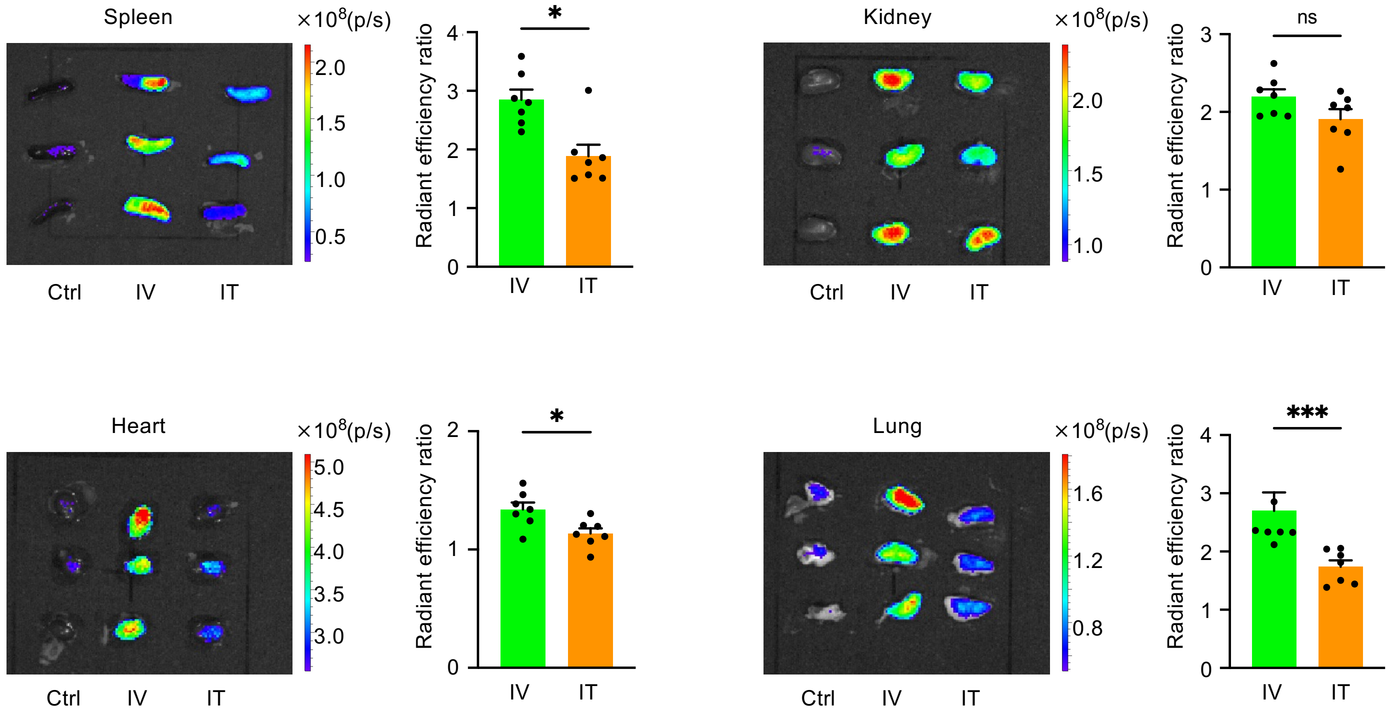

Supplementary Figure S2

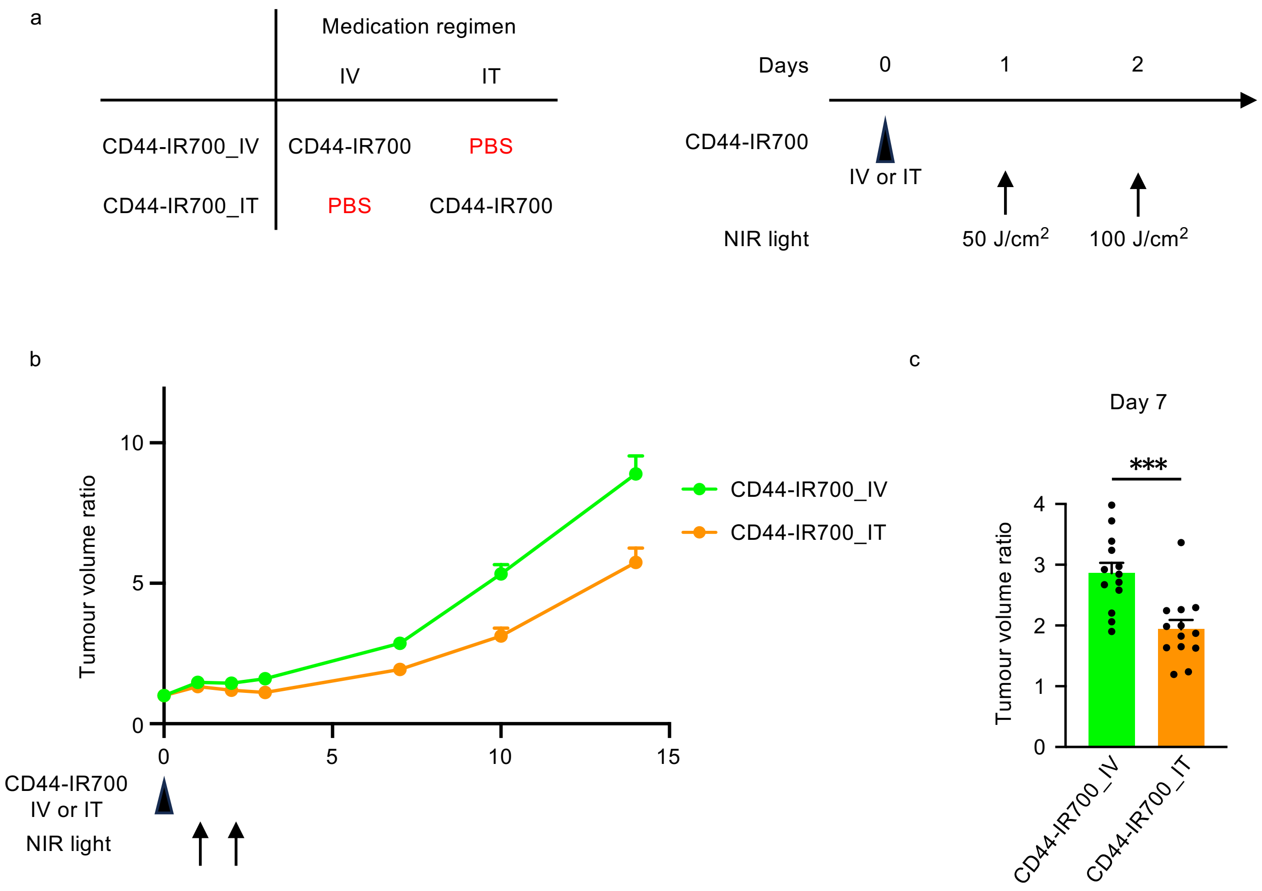

Supplementary Figure S3

a

	Medication regimen	
	IV	IT
Ctrl	(-)	(-)
free-IR700_IV	free-IR700	(-)
free-IR700_IT	(-)	free-IR700

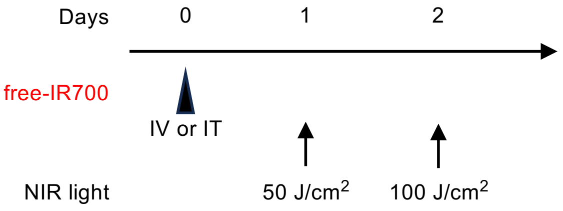

b

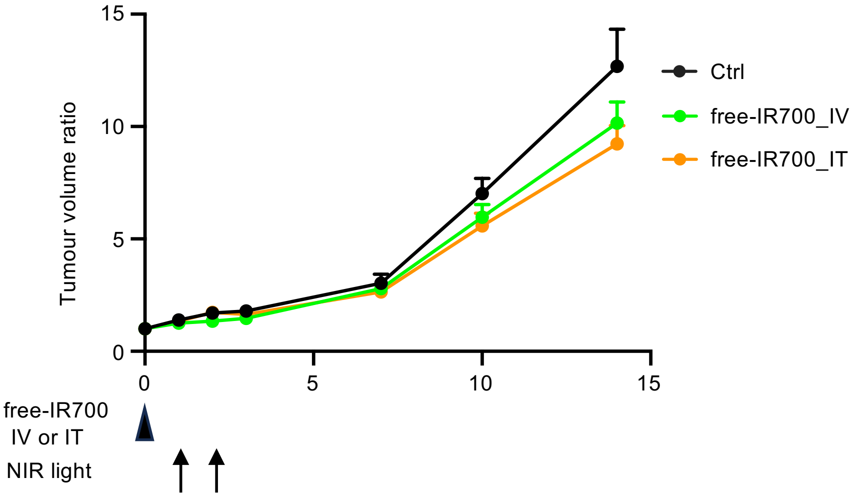

c

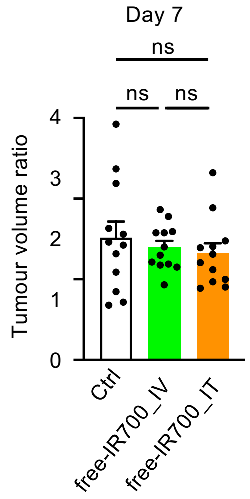

Supplementary Figure S4

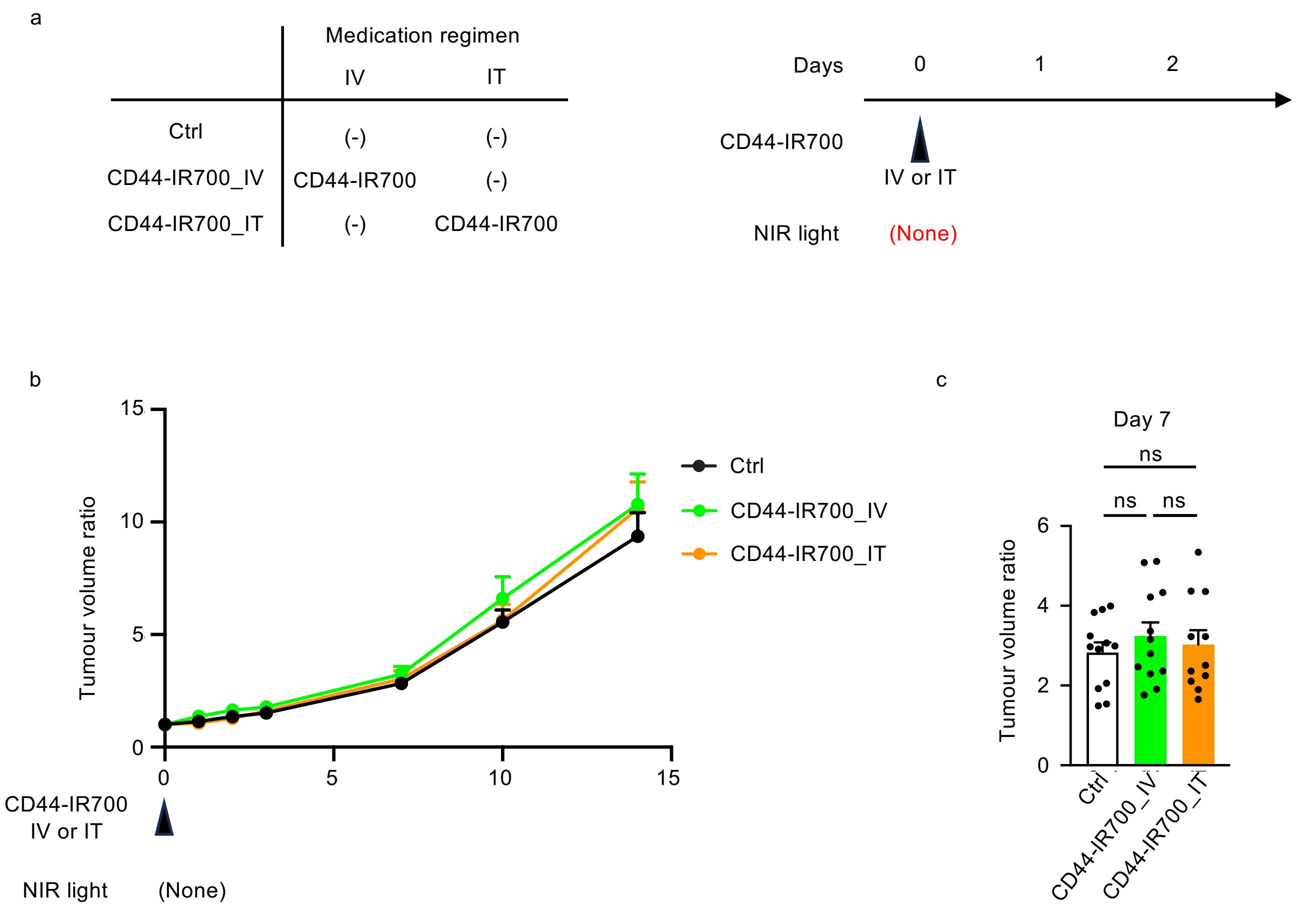

Supplementary Figure S5

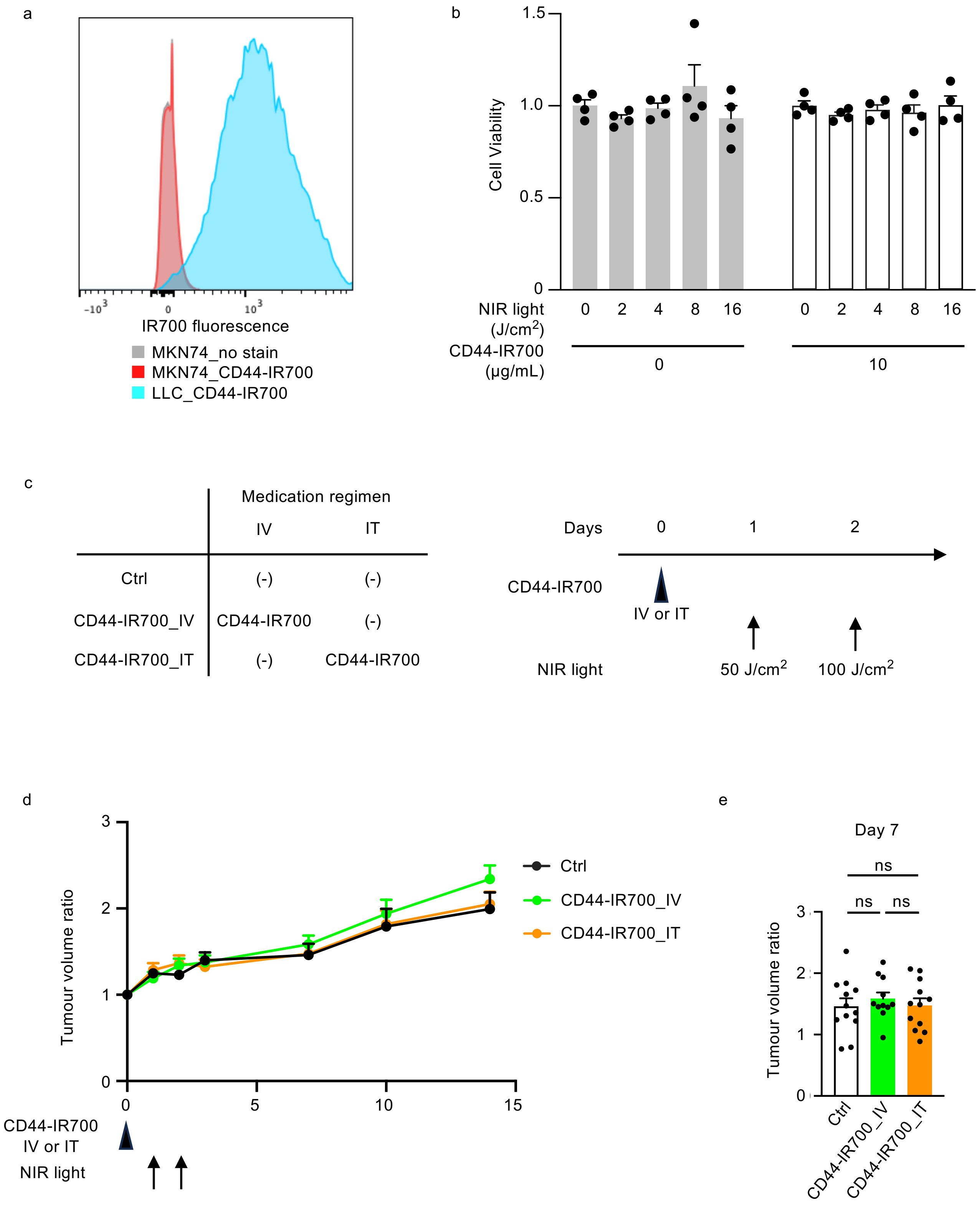

Supplementary Figure S6

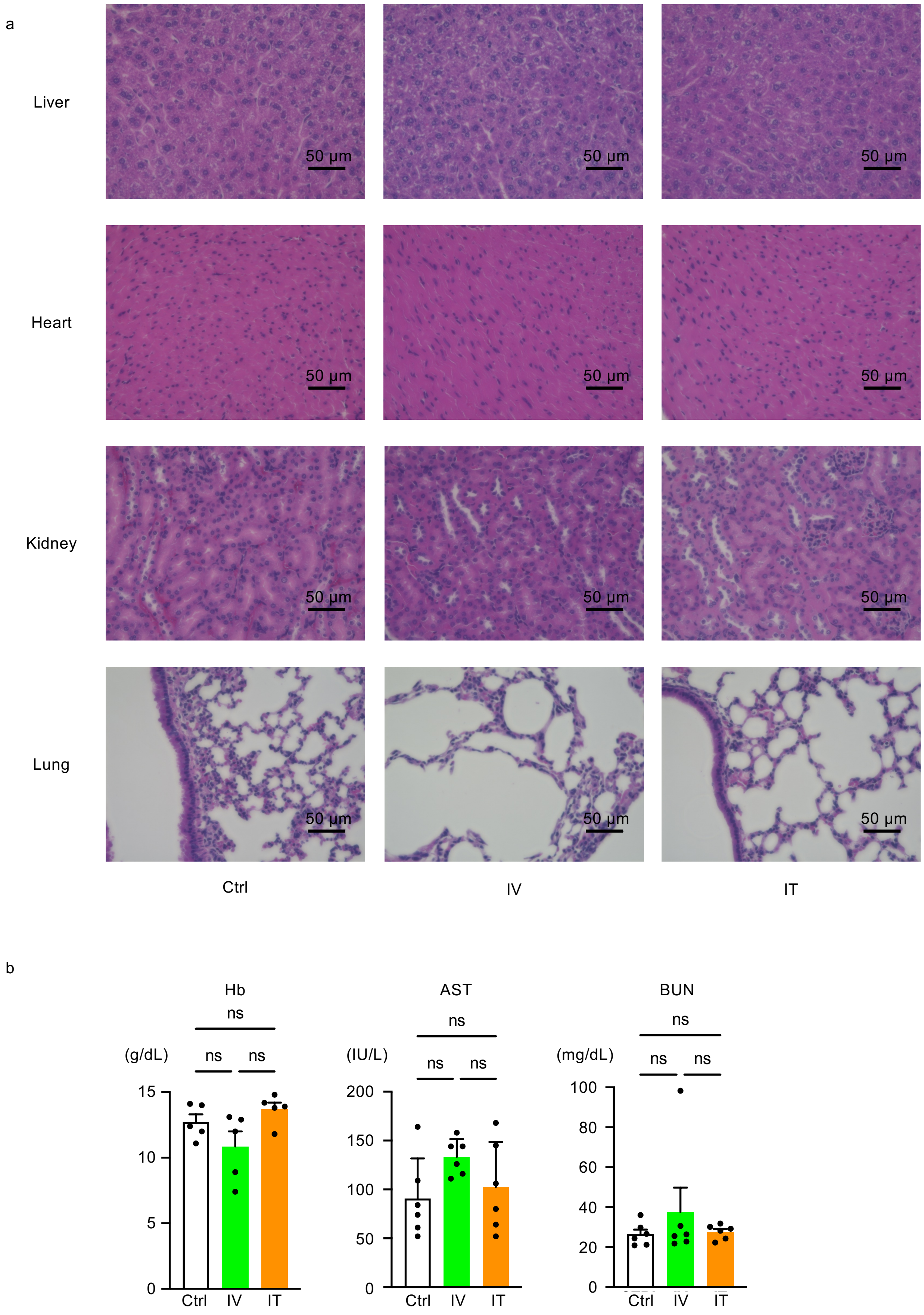

Supplement: Supplementary Figures [file mmc1.pdf]
