# Supplementary material for: Enhancing the efficacy of near-infrared photoimmunotherapy through intratumoural delivery of CD44–targeting antibody–photoabsorber conjugates
Source: eBioMedicine. 2025 Jan 22;112:105566. doi: 10.1016/j.ebiom.2025.105566 (PMC11795636; doi:10.1016/j.ebiom.2025.105566)
Supplement: Supplementary Figure Caption [file mmc2.docx]

**Supplementary Figure 1. Biodistribution of CD44-IR700 in normal tissues**

Biodistribution of CD44-IR700 was visualised in normal tissues. Treatments were administered in each group: Ctrl, IV (50 μg), and IT (50 μg) CD44-IR700 administration. The bar graph on the right shows the fluorescence values of the intravenous and intratumoural administration groups compared to the Ctrl group (n = 7 per group).

Data are presented as mean + standard error of the mean. Statistical difference was evaluated using Mann–Whitney U tests, with asterisks denoting levels of significance (**p* < 0.05, ****p* < 0.001). ‘ns’ indicates a lack of statistical significance.

CD44-IR700, IR700-conjugated anti-CD44 monoclonal antibody; Ctrl, control; IV, intravenous; and IT, intratumoural.

**Supplementary Figure 2. Intratumoural delivery of CD44-IR700 enhanced the efficacy of photoimmunotherapy *in vivo*, independent of direct physical damage to the tumour**

(a) Schematic of the experiment. Subcutaneous tumours were established in mice using Lewis Lung carcinoma cells. The mice were divided into two groups based on the treatment received: the CD44-IR700_IV group received intravenous the CD44-IR700 conjugate administration and intratumoural PBS, and the CD44-IR700_IT group received intratumoural CD44-IR700 conjugate administration and intravenous PBS. Tumours were exposed to NIR light at the indicated time points.

(b) Tumour volumes were measured on the day of CD44-IR700 administration, designated as day 0, and plotted as the ratio to the initial tumour size on the day of treatment initiation (n = 13 per group).

(c) Tumour volume ratio on day 7 relative to day 0 (n = 13 per group).

Values are expressed as mean + standard error of the mean. Statistical difference was assessed using Mann–Whitney U tests, with asterisks denoting significance levels (****p* < 0·001).

CD44-IR700, IR700-conjugated anti-CD44 monoclonal antibody; PBS, phosphate-buffered saline; CD, cluster of differentiation; NIR, near-infrared; IV, intravenous; and IT, intratumoural.

**Supplementary Figure 3. Ineffectiveness of photoimmunotherapy using antibody-unconjugated free IR700**

(a) Schematic of the experimental schedule. Mice bearing subcutaneous Lewis Lung carcinoma tumours were treated with either no free IR700 (Ctrl), free IR700 via IV injection (free-IR700_IV), or free IR700 via IT injection (free-IR700_IT), followed by NIR light irradiation on subsequent days.

(b) Tumour volume monitoring. Tumour size was measured starting from the day of free IR700 administration (day 0), and the change in size was expressed as a ratio relative to the initial volume on day 0 (n = 12 per group).

(c) Tumour volume ratio on day 7. The graph shows the fold increase in tumour volume on day 7 compared with that on day 0 (n = 12 per group).

Values are expressed as mean + standard error of the mean. Statistical difference was assessed using Kruskal-Wallis test. ‘ns’ indicates a lack of statistical significance.

CD44-IR700, IR700-conjugated anti-CD44 monoclonal antibody; CD, cluster of differentiation; NIR, near-infrared; Ctrl, control; IV, intravenous; and IT, intratumoural.

**Supplementary Figure 4. Ineffectiveness of photoimmunotherapy without near-infrared light irradiation.**

(a) Schematic of the experimental schedule. Mice bearing subcutaneous Lewis Lung carcinoma tumours were treated with either no CD44-IR700 (Ctrl), CD44-IR700 via IV injection (CD44-IR700_IV), or CD44-IR700 via IT injection (CD44-IR700_IT), without NIR light irradiation on subsequent days.

(b) Tumour volume monitoring. Tumour size was measured starting from the day of CD44-IR700 administration (day 0), and the change in size was expressed as a ratio relative to the initial volume on day 0 (n = 11-12 per group).

(c) Tumour volume ratio on day 7. The graph shows the fold increase in tumour volume on day 7 compared with day 0 (n = 11-12 per group).

Values are expressed as mean + standard error of the mean. Statistical difference was assessed using Kruskal-Wallis test. ‘ns’ indicates a lack of statistical significance.

CD44-IR700, IR700-conjugated anti-CD44 monoclonal antibody; CD, cluster of differentiation; NIR, near-infrared; Ctrl, control; IV, intravenous; and IT, intratumoural.

**Supplementary Figure 5. Lack of CD44-IR700-based photoimmunotherapy effect on CD44-negative tumour cells**

(a) Flow cytometry analysis of the interaction between MKN74 cells and Lewis Lung carcinoma (LLC) cells with CD44-IR700.

(b) Assessment of cell viability by a colorimetric assay following photoimmunotherapy with CD44-IR700 in MKN74 cells. Cells were cultured in a medium with or without CD44-IR700 and subsequently exposed to NIR light. Viability was measured and normalised against cells grown in a medium without CD44-IR700 and not exposed to NIR light (n = 4 for each bar).

(c) Schematic of the experimental schedule. Mice bearing subcutaneous MKN74 tumours were treated with either no CD44-IR700 (Ctrl), CD44-IR700 via IV injection (CD44-IR700_IV), or CD44-IR700 via IT injection (CD44-IR700_IT), followed by NIR light irradiation on subsequent days.

(d) Tumour volume monitoring. Tumour size was measured starting from the day of CD44-IR700 administration (day 0), and the change in size was expressed as a ratio relative to the initial volume on day 0 (n = 11-12 per group).

(e) Tumour volume ratio on day 7. The graph shows the fold increase in tumour volume on day 7 compared with day 0 (n = 11-12 per group).

Values are expressed as mean + standard error of the mean. Statistical difference was assessed using Kruskal-Wallis test. ‘ns’ indicates a lack of statistical significance.

CD44-IR700, IR700-conjugated anti-CD44 monoclonal antibody; CD, cluster of differentiation; NIR, near-infrared; Ctrl, control; IV, intravenous; and IT, intratumoural.

**Supplementary Figure 6.** **Safety evaluation screening of CD44-IR700**

(a) Representative images of HE staining of the liver, heart, kidney, and lung on day 7 post-treatment from each therapeutic group. The scale bar represents 50 μm.

(b) Blood test results on day 7 post-treatment from each therapeutic group (n = 5-6 per group).

Data are presented as mean + standard error of the mean. Statistical difference was evaluated using Kruskal-Wallis test. ‘ns’ indicates a lack of statistical significance.

CD44-IR700, IR700-conjugated anti-CD44 monoclonal antibody; HE, haematoxylin eosin; Ctrl, control; IV, intravenous; IT, intratumoural ; Hb, haemoglobin; AST, aspartate aminotransferase; and BUN, blood urea nitrogen.
