# Supplementary material for: Enhancing the efficacy of near-infrared photoimmunotherapy through intratumoural delivery of CD44–targeting antibody–photoabsorber conjugates
Source: eBioMedicine. 2025 Jan 22;112:105566. doi: 10.1016/j.ebiom.2025.105566 (PMC11795636; doi:10.1016/j.ebiom.2025.105566)
Supplement: Supplementary Material [file mmc3.pdf]

October 17, 2024

BEX

BEX CO.,LTD.
2-61-14 Itabashi Itabashi-ku, Tokyo, Japan
TEL : +81-3-5375-1071
FAX : +81-3-5375-5636

Cell Line Authentication

Client : Adachi Yuichi
Department of Respiratory Medicine and Clinical Immunology, Graduate School of Medicine, Osaka University

Analysis conducted by Katsunori Imai

Sample：MKN74

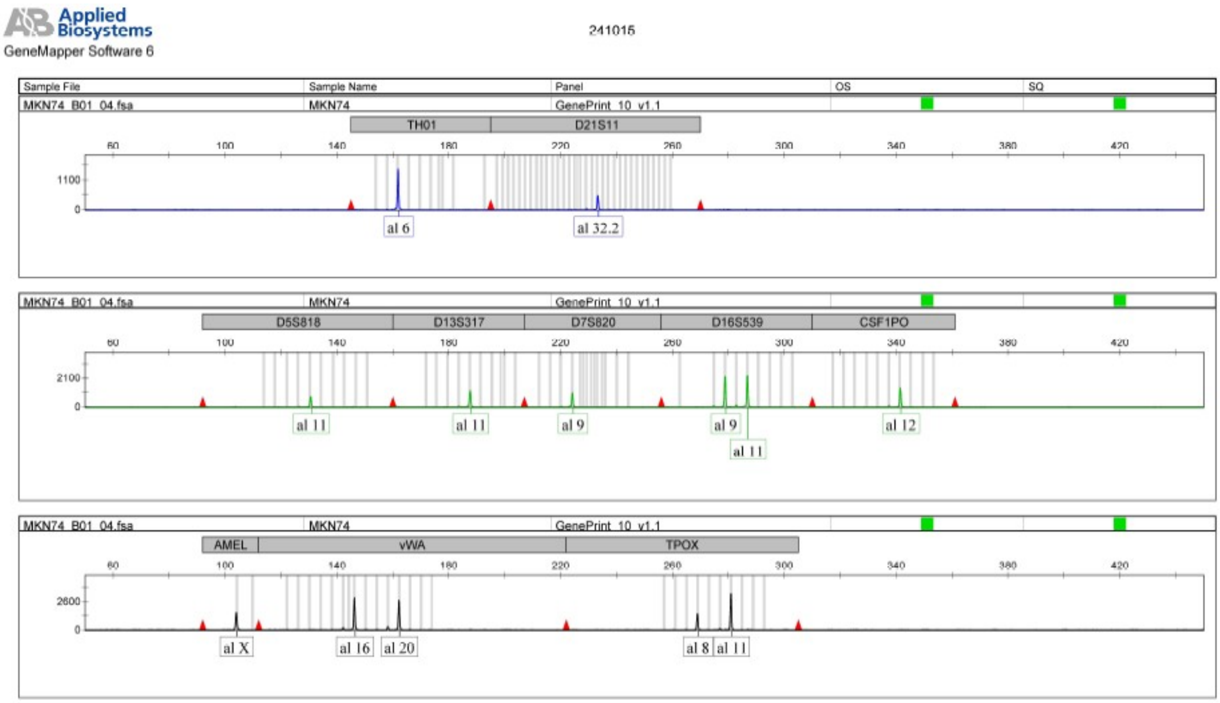

STR Profile

Locus	MKN74		MKN74 (CVCL_2791)	
TH01	6		6	
D21S11	32.2		32.2	
D5S818	11		11	
D13S317	11		11	
D7S820	9		9	
D16S539	9	11	9	11
CSF1PO	12		12	
AMEL	X		X	
vWA	16	20	16	20
TPOX	8	11	8	11

STR profiles of MKN74 and MKN74 (CVCL_2791) were completely matched. It was verified that the cells analyzed were considered to be the same as the cells registered in ExPASy by comparison with the database of ExPASy.

Reference:

Cell line individualization by STR multiplex system in the cell bank found cross-contamination between ECV304, and EJ-1/T24. Tissue Culture Research Communications,18:329-338(1999)
Tanabe,H., Takada,Y., Minegishi,D., Kurematsu,M. Masui,T., and Mizusawa,H.

Check your cultures! A list of cross-contaminated or misidentified cell lines. Int J Cancer. 2010 Jul 1;127(1):1-8.
Capes-Davis A1, Theodosopoulos G, Atkin I, Drexler HG, Kohara A, MacLeod RA, Masters JR, Nakamura Y, Reid YA, Reddel RR, Freshney RI.

Technical Data Sheet

InVivoMAb anti-mouse/human CD44

Attention: Use of this product constitutes an agreement to Bio X Cell's Terms and Conditions which are included with this product in print and can also be found at https://bioxcell.com/terms-and-conditions.

Lot Specific Information

**Lot Number:**	**Lot Specific***
**Volume:**	**Lot Specific***
**Concentration:**	**Lot Specific* (generally 4 to 11 mg/ml) ***
**Total Protein:**	**Lot Specific***

*This information will be noted on the certificate of analysis that ships with this product.

Product Information

**Catalog Number:**	**BE0039**
**Clone:**	**IM7**
**Isotype:**	Rat IgG2b, κ
**Recommended Isotype Control(s):**	InVivoMAb rat IgG2b isotype control, anti-keyhole limpet hemocyanin
**Recommended Dilution Buffer:**	InVivoPure pH 7.0 Dilution Buffer
**Immunogen:**	Dexamethasone-induced myeloid leukemia M1 cells
**Reported Applications:**	*in vivo* CD44 neutralization<br>*in vitro* CD44 neutralization
**Formulation:**	PBS, pH 7.0<br>Contains no stabilizers or preservatives
**Endotoxin:**	<2EU/mg (<0.002EU/μg)<br>Determined by LAL gel clotting assay
**Purity:**	>95%<br>Determined by SDS-PAGE
**Sterility:**	0.2 μm filtered
**Production:**	Purified from cell culture supernatant in an animal-free facility
**Purification:**	Protein G
**RRID:**	AB_1107649
**Molecular Weight:**	150 kDa

Description

The IM7 monoclonal antibody reacts with human and mouse CD44 also known as Hermes, HCAM, and Pgp-1. CD44 is an 80-95 kDa glycoprotein that is expressed on all leukocytes, endothelial cells, hepatocytes, and mesenchymal cells. As an adhesion molecule, CD44 participates in a wide variety of cellular functions including lymphocyte activation, recirculation and homing, and hematopoiesis. CD44 is a receptor for hyaluronic acid and can also interact with other ligands, such as osteopontin, collagens, and matrix metalloproteinases (MMPs). Additionally, CD44 is involved in tumor metastasis and targeting of CD44 by antibodies has been shown to reduce the malignant activities of various neoplasms. Interestingly, high levels of the adhesion molecule CD44 on leukemic cells are essential to generate leukemia. The IM7 antibody has been shown to neutralize CD44 in vivo

Storage

Store at the stock concentration at 4°C. **Do not freeze.**
It is not uncommon for a floccule or precipitate to appear during storage. The floccule is typically buffer salts precipitating out of solution or a small bit of protein aggregation. For information on how to remove floccules or precipitates see our FAQ's at https://bioxcell.com/faqs.

Protocol Information

Since applications vary, each investigator should use the application references as a guide to help estimate the appropriate dose or concentration. The dose or concentration can be further optimized experimentally in a dose response or titration experiment.

Application References

For a complete list of references, visit https://bioxcell.com/catalogsearch/result/?q=BE0039#tab_references or scan the QR code below.

Bio X Cell, LLC
https://bioxcell.com
+1-866-787-3444
customerservice@bioxcell.com

Conditions: For research use only. Not for use in diagnostic or therapeutic procedures.
Not for resale.
Bio X Cell, Bio X Cell logo, and all other trademarks are the property of Bio X Cell, LLC © 2024 Bio X Cell, LLC

PE/Cyanine7 anti-mouse CD3ε Antibody

Catalog# / Size 100319 / 25 µg
100320 / 100 µg

Clone 145-2C11

Regulatory Status RUO

Other Names CD3ε, T3, CD3

Isotype Armenian Hamster IgG

Description CD3ε is a 20 kD transmembrane protein, also known as CD3 or T3. It is a member of the Ig superfamily and primarily expressed on T cells, NK-T cells, and at different levels on thymocytes during T cell differentiation. CD3ε forms a TCR complex by associating with the CD3δ, γ and ζ chains, as well as the TCR α/β or γ/δ chains. CD3 plays a critical role in TCR signal transduction, T cell activation, and antigen recognition by binding the peptide/MHC antigen complex.

Product Details

Verified Reactivity Mouse

Antibody Type Monoclonal

Host Species Armenian Hamster

Immunogen H-2K^{b}-specific mouse cytotoxic T lymphocyte clone BM10-37

Formulation Phosphate-buffered solution, pH 7.2, containing 0.09% sodium azide.

Preparation The antibody was purified by affinity chromatography, and conjugated with PE/Cyanine7 under optimal conditions.

Concentration 0.2 mg/ml

Storage & Handling The antibody solution should be stored undiluted between 2°C and 8°C, and protected from prolonged exposure to light. **Do not freeze.**

Application FC - Quality tested

Recommended Usage Each lot of this antibody is quality control tested by immunofluorescent staining with flow cytometric analysis. For flow cytometric staining, the suggested use of this reagent is ≤0.5 µg per million cells in 100 µl volume. It is recommended that the reagent be titrated for optimal performance for each application.

Excitation Laser Blue Laser (488 nm)
Green Laser (532 nm)/Yellow-Green Laser (561 nm)

Application Notes Clone 145-2C11 is useful for *in vitro* blocking of target-specific CTL-mediated cell lysis[1], as well as T cell activation assays, inducing proliferation and cytokine production[1,2,7,12,16]. It also induces apoptosis in immature thymocytes[32], and *in vivo* T cell depletion[8-10]. Additional reported applications (for relevant formats of this clone) include: immunoprecipitation[1], immunohistochemical staining[14,15] of acetone-fixed frozen sections and zinc-fixed paraffin-embedded sections, Western blotting[4], complement-mediated cytotoxicity[6], *in vitro* and *in vivo* stimulation of T cells[1,2,7,12,16], immunofluorescent staining[5], and *in vivo* T cell depletion8-10. The 145-2C11 antibody has been reported to block the binding of 17A2 antibody to CD3 epsilon-specific T cells[11]. Clone 145-2C11 is not recommended for formalin-fixed paraffin embedded sections. The LEAF™ purified antibody (Endotoxin <0.1 EU/µg, Azide-Free, 0.2 µm filtered) is recommended for functional assays (Cat. No. 100314). For *in vivo* studies or highly sensitive assays, we recommend Ultra-LEAF™ purified antibody (Cat. No. 100340) with a lower endotoxin limit than standard LEAF™ purified antibodies (Endotoxin <0.01 EU/µg).

Application References

(PubMed link indicates BioLegend citation)

1. Leo O, *et al.* 1987. *P. Natl. Acad. Sci. USA* 84:1374. (IP, Activ, Block)
2. Kruisbeek AM, *et al.* 1991. *In Current Protocols in Immunology.* 3.12.1. (Activ)
3. Duke RC, *et al.* 1995. *Current Protocols in Immunology.* 3.17.1.
4. Salvadori S, *et al.* 1994. *J. Immunol.* 153:5176. (WB)
5. Payer E, *et al.* 1991. *J. Immunol.* 146:2536. (IF)
6. Jacobs H, *et al.* 1994. *Eur. J. Immunol.* 24:934. (CMCD)
7. Vossen ACTM, *et al.* 1995. *Eur. J. Immunol.* 25:1492. (Activ)
8. Henrickson M, *et al.* 1995. *Transplantation* 60:828. (Deplete)
9. Kinnaert P, *et al.* 1996. *Transpl. Int.* 9:386. (Deplete)
10. Han WR, *et al.* 1999. *Transpl. Immunol.* 7:207. (Deplete)
11. Miescher GC, *et al.* 1989. *Immunol. Lett.* 23:113. (Block)
12. Terrazas LI, *et al.* 2005. *Intl. J. Parasitology.* 35:1349. (Activ)

See More

Product Citations

1. Tran NT, *et al.* 2019. Cell Rep. 28:3510. PubMed
2. Strickley JD, *et al.* 2019. Nature. 575:519. PubMed
3. Dong MB, *et al.* 2020. Cell. 178(5):1189-1204.e23.. PubMed
4. Deák P, *et al.* 2022. Cell Rep. 41:111563. PubMed
5. Latour YL, *et al.* 2023. Gut Microbes. 15:2192623. PubMed
6. Palakurthi B, *et al.* 2023. Nat Commun. 14:2109. PubMed
7. Wang Z, *et al.* 2023. J Cancer. 14:1049. PubMed
8. Aguilar EG, *et al.* 2021. Blood Adv. 5:4219. PubMed
9. Meng J, *et al.* 2022. Cell Rep. 38:110492. PubMed
10. Ocaña-Guzman R, *et al.* 2022. Cells. 11: . PubMed
11. Zhang Y, *et al.* 2022. Vaccines (Basel). 10: . PubMed
12. Dölz M, *et al.* 2022. iScience. 25:105372. PubMed

RRID

AB_312684 (BioLegend Cat. No. 100319)
AB_312685 (BioLegend Cat. No. 100320)

Antigen Details

Structure

Ig superfamily, forms CD3/TCR complex with CD3δ, γ and ζ subunits and TCR (α/β and γ/δ), 20 kD

Distribution

Thymocytes (differentiation dependent), mature T cells, NK-T cells

Function

TCR signal transduction, T cell activation, antigen recognition

Ligand/Receptor

Peptide antigen/MHC-complex

Cell Type

NKT cells, T cells, Thymocytes, Tregs

Biology Area

Immunology

Molecular Family

CD Molecules, TCRs

Antigen References

1. Barclay A, *et al.* 1997. The Leukocyte Antigen FactsBook Academic Press.
2. Davis MM. 1990. *Annu. Rev. Biochem.* 59:475.
3. Weiss A, *et al.* 1994. *Cell* 76:263.

Gene ID

12501

Related Protocols

- Cell Surface Flow Cytometry Staining Protocol

Other Formats

APC anti-mouse CD3ε, Biotin anti-mouse CD3ε, FITC anti-mouse CD3ε, PE anti-mouse CD3ε, PE/Cyanine5 anti-mouse CD3ε, Purified anti-mouse CD3ε, PE/Cyanine7 anti-mouse CD3ε, Alexa Fluor® 488 anti-mouse CD3ε, Alexa Fluor® 647 anti-mouse CD3ε, PerCP anti-mouse CD3ε, PerCP/Cyanine5.5 anti-mouse CD3ε, Purified anti-mouse CD3ε (Maxpar® Ready), APC/Cyanine7 anti-mouse CD3ε, Pacific Blue™ anti-mouse CD3ε, Brilliant Violet 421™ anti-mouse CD3ε, Ultra-LEAF™ Purified anti-mouse CD3ε, PE/Dazzle™ 594 anti-mouse CD3ε, Brilliant Violet 510™ anti-mouse CD3ε, Brilliant Violet 605™ anti-mouse CD3ε, Brilliant

Violet 711™ anti-mouse CD3ε, Brilliant Violet 785™ anti-mouse CD3ε, APC/Fire™ 750 anti-mouse CD3ε, GoInVivo™ Purified anti-mouse CD3ε, Spark YG™ 593 anti-mouse CD3, PerCP/Fire™ 780 anti-mouse CD3ε, Brilliant Violet 650™ anti-mouse CD3ε

Product Data

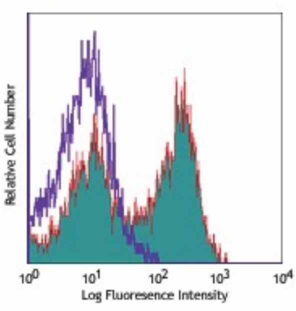

C57BL/6 mouse splenocytes were stained with CD3e (clone 145-2C11) PE/Cyanine7 (filled histogram) or Armenian hamster IgG PE/Cyanine7 isotype control (open histogram).

For Research Use Only. Not for diagnostic or therapeutic use.

This product is supplied subject to the terms and conditions, including the limited license, located at www.biolegend.com/terms) ("Terms") and may be used only as provided in the Terms. Without limiting the foregoing, BioLegend products may not be used for any Commercial Purpose as defined in the Terms, resold in any form, used in manufacturing, or reverse engineered, sequenced, or otherwise studied or used to learn its design or composition without express written approval of BioLegend. Regardless of the information given in this document, user is solely responsible for determining any license requirements necessary for user's intended use and assumes all risk and liability arising from use of the product. BioLegend is not responsible for patent infringement or any other risks or liabilities whatsoever resulting from the use of its products.

BioLegend, the BioLegend logo, and all other trademarks are property of BioLegend, Inc. or their respective owners, and all rights are reserved.

8999 BioLegend Way, San Diego, CA 92121 www.biolegend.com
Toll-Free Phone: 1-877-Bio-Legend (246-5343) Phone: (858) 768-5800 Fax: (877) 455-9587

APC/Cyanine7 anti-mouse CD45 Antibody

**Catalog# / Size**	103115 / 25 µg<br>103116 / 100 µg
**Clone**	30-F11
**Regulatory Status**	RUO
**Other Names**	T200, Ly-5, LCA
**Isotype**	Rat IgG2b, κ
**Description**	CD45 is a 180-240 kD glycoprotein also known as the leukocyte common antigen (LCA), T200, or Ly-5. It is a member of the protein tyrosine phosphatase (PTP) family, expressed on all hematopoietic cells except mature erythrocytes and platelets. There are different isoforms of CD45 that arise from variable splicing of exons 4, 5, and 6, which encode A, B, and C determinants, respectively. CD45 plays a key role in TCR and BCR signal transduction. These isoforms are very specific to the activation and maturation state of the cell as well as cell type. The primary ligands for CD45 are galectin-1, CD2, CD3, CD4, TCR, CD22, and Thy-1.

Product Details

**Verified Reactivity**	Mouse
**Antibody Type**	Monoclonal
**Host Species**	Rat
**Immunogen**	Mouse thymus or spleen
**Formulation**	Phosphate-buffered solution, pH 7.2, containing 0.09% sodium azide.
**Preparation**	The antibody was purified by affinity chromatography, and conjugated with APC/Cyanine7 under optimal conditions.
**Concentration**	0.2 mg/ml
**Storage & Handling**	The antibody solution should be stored undiluted between 2°C and 8°C, and protected from prolonged exposure to light. **Do not freeze.**
**Application**	*FC - Quality tested*
**Recommended Usage**	Each lot of this antibody is quality control tested by immunofluorescent staining with flow cytometric analysis. For flow cytometric staining, the suggested use of this reagent is = 0.25 µg per 10^6 cells in 100 µl volume. It is recommended that the reagent be titrated for optimal performance for each application.
**Excitation Laser**	Red Laser (633 nm)
**Application Notes**	Clone 30-F11 reacts with all isoforms and both CD45.1 and CD45.2 alloantigens of CD45.<br><br>Additional reported applications (for relevant formats) include: immunoprecipitation[3], complement-dependent cytotoxicity[1,5], immunohistochemistry (acetone-fixed frozen sections, zinc-fixed paraffin-embedded sections and formalin-fixed paraffin-embedded sections)[4,6], Western blotting[7], and spatial biology (IBEX)[10,11]. The Ultra-LEAF™ purified antibody (Endotoxin < 0.01 EU/µg, Azide-Free, 0.2 µm filtered) is recommended for functional assays (Cat. No. 103163 and 103164).

Application References

(PubMed link indicates BioLegend citation)

1. Podd BS, *et al.* 2006. *J. Immunol.* 176:6532. (FC, CMCD) PubMed
2. Haynes NM, *et al.* 2007. *J. Immunol.* 179:5099. (FC)
3. Ledbetter JA, *et al.* 1979. *Immunol. Rev.* 47:63. (IP)
4. Simon DI, *et al.* 2000. *J. Clin. Invest.* 105:293. (IHC)
5. Seaman WE. 1983. *J. Immunol.* 130:1713. (CMCD)
6. Cornet A, *et al.* 2001. *P. Natl. Acad. Sci. USA* 98:13306. (IHC)
7. Tsuboi S and Fukuda M. 1998. *J. Biol. Chem.* 273:30680. (WB) PubMed
8. Liu F, *et al.* 2012. *Blood.* 119:3295. PubMed
9. Pelletier AN, *et al.* 2012. *J. Immunol.* 188:5561. PubMed
10. Radtke AJ, *et al.* 2020. *Proc Natl Acad Sci U S A.* 117:33455-65. (SB) PubMed
11. Radtke AJ, *et al.* 2022. *Nat Protoc.* 17:378-401. (SB) PubMed

Product Citations

1. Cignarella F *et al.* 2018. Cell metabolism. 27(6):1222-1235 . PubMed
2. Contijoch EJ *et al.* 2019. eLife. 8 pii: e40553. PubMed
3. Wu J *et al.* 2017. Immunity. 47(6):1114-1128 . PubMed
4. Komuczki J, *et al.* 2019. Immunity. 50:1289. PubMed
5. Wang X, *et al.* 2019. Cell Res. 29:787. PubMed
6. Yu X, *et al.* 2020. Nat Commun. 11:1110. PubMed
7. Korsunsky I, *et al.* 2022. Med. 3:481. PubMed
8. Cheng C, *et al.* 2022. Cell Mol Gastroenterol Hepatol. 15:261. PubMed
9. Pattwell SS, *et al.* 2022. Sci Adv. 8:eabo6789. PubMed
10. Schroeter CB, *et al.* 2022. J Neuroinflammation. 19:270. PubMed
11. Ho HM, *et al.* 2022. Pharmaceutics. 14:. PubMed
12. Cai Z, *et al.* 2023. Adv Sci (Weinh). 10:e2207155. PubMed

RRID AB_312980 (BioLegend Cat. No. 103115)
AB_312981 (BioLegend Cat. No. 103116)

Antigen Details

Structure Protein tyrosine phosphatase (PTP) family, 180-240 kD

Distribution All hematopoietic cells except mature erythrocytes and platelets

Function Phosphatase, T and B cell activation

Ligand/Receptor Galectin-1, CD2, CD3, CD4, TCR, CD22, Thy-1

Cell Type B cells, Dendritic cells, Mesenchymal Stem Cells, Tregs

Biology Area Cell Biology, Immunology, Inhibitory Molecules, Innate Immunity, Neuroscience, Neuroscience Cell Markers, Stem Cells

Molecular Family CD Molecules

Antigen References

1. Barclay A, *et al.* 1997. The Leukocyte Antigen FactsBook Academic Press.
2. Trowbridge IS, *et al.* 1993. *Annu. Rev. Immunol.* 12:85.
3. Kishihara K, *et al.* 1993. *Cell* 74:143.
4. Pulido R, *et al.* 1988. *J. Immunol.* 140:3851.

Gene ID 19264

Related Protocols

- Cell Surface Flow Cytometry Staining Protocol

Other Formats

APC anti-mouse CD45, Biotin anti-mouse CD45, FITC anti-mouse CD45, PE anti-mouse CD45, PE/Cyanine5 anti-mouse CD45, Purified anti-mouse CD45, PE/Cyanine7 anti-mouse CD45, APC/Cyanine7 anti-mouse CD45, Alexa Fluor® 488 anti-mouse CD45, Alexa Fluor® 647 anti-mouse CD45, Pacific Blue™ anti-mouse CD45, Alexa Fluor® 700 anti-mouse CD45, PerCP/Cyanine5.5 anti-mouse CD45, PerCP anti-mouse CD45, Alexa Fluor® 594 anti-mouse CD45, Brilliant Violet 421™ anti-mouse CD45, Brilliant Violet 570™ anti-mouse CD45, Brilliant Violet 510™ anti-mouse CD45, Brilliant Violet 605™ anti-mouse CD45, Purified anti-mouse CD45 (Maxpar® Ready), PE/Dazzle™ 594 anti-mouse CD45, Brilliant Violet 711™ anti-mouse CD45, Brilliant Violet 785™ anti-mouse

CD45, Brilliant Violet 650™ anti-mouse CD45, APC/Fire™ 750 anti-mouse CD45, Brilliant Violet 750™ anti-mouse CD45, TotalSeq™-A0096 anti-mouse CD45, TotalSeq™-B0096 anti-mouse CD45, Ultra-LEAF™ Purified anti-mouse CD45, Spark Blue™ 550 anti-mouse CD45, Spark NIR™ 685 anti-mouse CD45, TotalSeq™-C0096 anti-mouse CD45, Spark YG™ 570 anti-mouse CD45, PE/Fire™ 640 anti-mouse CD45, APC/Fire™ 810 anti-mouse CD45, PE/Fire™ 700 anti-mouse CD45, Spark Violet™ 538 anti-mouse CD45, Spark YG™ 593 anti-mouse CD45, Spark Blue™ 574 anti-mouse CD45 Antibody, Spark Blue™ 515 anti-mouse CD45, Spark UV™ 387 anti-mouse CD45, PE/Fire™ 810 anti-mouse CD45, Spark Red™ 718 anti-mouse CD45 (Flexi-Fluor™), Spark PLUS UV395™ anti-mouse CD45

Product Data

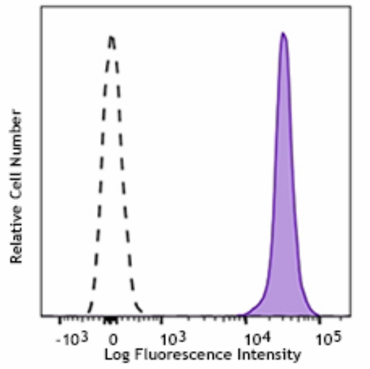

C57BL/6 splenocytes were stained with CD45 (clone 30-F11) APC/Cyanine7 (filled histogram) or Rat IgG2b, κ APC/Cyanine7 isotype control (open histogram).

For Research Use Only. Not for diagnostic or therapeutic use.

This product is supplied subject to the terms and conditions, including the limited license, located at www.biolegend.com/terms) ("Terms") and may be used only as provided in the Terms. Without limiting the foregoing, BioLegend products may not be used for any Commercial Purpose as defined in the Terms, resold in any form, used in manufacturing, or reverse engineered, sequenced, or otherwise studied or used to learn its design or composition without express written approval of BioLegend. Regardless of the information given in this document, user is solely responsible for determining any license requirements necessary for user's intended use and assumes all risk and liability arising from use of the product. BioLegend is not responsible for patent infringement or any other risks or liabilities whatsoever resulting from the use of its products.

BioLegend, the BioLegend logo, and all other trademarks are property of BioLegend, Inc. or their respective owners, and all rights are reserved.

8999 BioLegend Way, San Diego, CA 92121 www.biolegend.com
Toll-Free Phone: 1-877-Bio-Legend (246-5343) Phone: (858) 768-5800 Fax: (877) 455-9587

CD8a Monoclonal Antibody (53-6.7), PE, eBioscience™

Product Details	
Size	100 µg
Species Reactivity	Mouse
Published Species	Hamster, Mouse, Human
Host/Isotype	Rat / IgG2a, kappa
Recommended Isotype Control	Rat IgG2a kappa Isotype Control (eBR2a), PE, eBioscience™
Class	Monoclonal
Type	Antibody
Clone	53-6.7
Conjugate	PE
Excitation/Emission Max	565/576 nm
Form	Liquid
Concentration	0.2 mg/mL
Purification	Affinity chromatography
Storage buffer	PBS, pH 7.2
Contains	0.09% sodium azide
Storage conditions	4° C, store in dark, DO NOT FREEZE!
RRID	AB_465530

Applications	Tested Dilution	Publications
Western Blot (WB)	-	1 Publication
Immunohistochemistry (IHC)	-	3 Publications
Immunohistochemistry (Paraffin) (IHC (P))	-	1 Publication
Immunohistochemistry (Frozen) (IHC (F))	-	1 Publication
Immunocytochemistry (ICC/IF)	-	2 Publications
Flow Cytometry (Flow)	0.25 µg/test	294 Publications
Functional Assay (FN)	-	3 Publications
Miscellaneous PubMed (Misc)	-	1 Publication

Product Specific Information

Description: The 53-6.7 monoclonal antibody reacts with the mouse CD8a molecule. CD8a is an approximately 32-34 kDa cell surface receptor expressed either as a heterodimer with the CD8 beta chain (CD8 alpha beta) or as a homodimer (CD8 alpha alpha). A majority of thymocytes and a subpopulation of mature alpha beta TCR T cells express CD8 alpha beta while gamma delta TCR T cells, a subpopulation of intestinal intraepithelial lymphocytes (IELs) and dendritic cells express CD8 alpha alpha. CD8 binds to MHC class I and through its association with protein tyrosine kinase p56lck plays a role in T cell development and activation of mature T cells.

Applications Reported: The 53-6.7 antibody has been reported for use in flow cytometric analysis.

Applications Tested: The 53-6.7 antibody has been tested by flow cytometric analysis of mouse thymocytes and splenocytes.

This can be used at less than or equal to 0.25 µg per test. A test is defined as the amount (µg) of antibody that will stain a cell sample in a final volume of 100 µL. Cell number should be determined empirically but can range from 10^5 to 10^8 cells/test. It is recommended that the antibody be carefully titrated for optimal performance in the assay of interest.

Excitation: 488-561 nm; Emission: 578 nm; Laser: Blue Laser, Green Laser, Yellow-Green Laser.

Filtration: 0.2 µm post-manufacturing filtered.

Product Images For CD8a Monoclonal Antibody (53-6.7), PE, eBioscience™

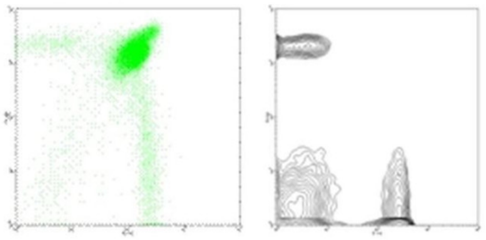

CD8a Antibody (12-0081-82) in Flow
Staining of mouse thymocytes (left) or splenocytes (right) with Anti-Mouse CD4 FITC (Product # 11-0041-82) and 0.125 µg of Anti-Mouse CD8a PE. Total viable cells were used for analysis.

View more figures on thermofisher.com

306 References

Western Blot (1)

EMBO molecular medicine

Unexpected PD-L1 immune evasion mechanism in TNBC, ovarian, and other solid tumors by DR5 agonist antibodies.

"12-0081-82 was used in Flow Cytometry, Western Blot to identify a potential clinically viable combinatorial strategy to revive solid cancer immunotherapy using death receptor agonism."

Authors: Mondal T,Shivange GN,Tihagam RG,Lyerly E,Battista M,Talwar D,Mosavian R,Urbanek K,Rashid NS,Harrell JC,Bos PD,Stelow EB,Stack MS,Bhatnagar S,Tushir-Singh J

Year
2021

Species
Mouse

Immunohistochemistry (3)

Immunity

E3 Ligase VHL Promotes Group 2 Innate Lymphoid Cell Maturation and Function via Glycolysis Inhibition and Induction of Interleukin-33 Receptor.

"12-0081 was used in Immunohistochemistry to indicate that the VHL-HIF-glycolysis axis is essential for the late-stage maturation and function of ILC2s via targeting IL-33-ST2 pathway."

Authors: Li Q,Li D,Zhang X,Wan Q,Zhang W,Zheng M,Zou L,Elly C,Lee JH,Liu YC

Year
2018

Species
Mouse

PLoS pathogens

Experimental cerebral malaria pathogenesis--hemodynamics at the blood brain barrier.

"12-0081 was used in Flow cytometry/Cell sorting to show that significantly more CD8(+) T cells, neutrophils, and macrophages are recruited to postcapillary venules during ECM compared to hyperparasitemia."

Authors: Nacer A,Movila A,Sohet F,Girgis NM,Gundra UM,Loke P,Daneman R,Frevert U

Year
2014

Species
Mouse

View more IHC references on thermofisher.com

Immunohistochemistry (Paraffin) (1)

The American journal of pathology

IL-27 inhibits hyperglycemia and pancreatic islet inflammation induced by streptozotocin in mice.

"12-0081 was used in Immunofluorescence to evaluate inflammatory cell infiltration to pancreatic islets in wild-type, EBI3 (-/-), and WSX-1(-/-) mice treated with streptozotocin."

Authors: Fujimoto H,Hirase T,Miyazaki Y,Hara H,Ide-Iwata N,Nishimoto-Hazuku A,Saris CJ,Yoshida H,Node K

Year
2011

Species
Mouse

More applications with references on thermofisher.com

IHC (F) (1) ICC/IF (2) Flow (294) FN (3) Misc (1)

For Research Use Only. Not for use in diagnostic procedures. Not for resale without express authorization. Products are warranted to operate or perform substantially in conformance with published Product specifications in effect at the time of sale, as set forth in the Production documentation, specifications and/or accompanying package inserts ("Documentation"). No claim of suitability for use in applications regulated by FDA is made. The warranty provided herein is valid only when used by properly trained individuals. Unless otherwise stated in the Documentation, this warranty is limited to one year from date of shipment when the Product is subjected to normal, proper and intended usage. This warranty does not extend to anyone other than the Buyer. Any model or sample furnished to Buyer is merely illustrative of the general type and quality of goods and does not represent that any Product will conform to such model or sample. NO OTHER WARRANTIES, EXPRESS OR IMPLED, ARE GRANTED INCLUDING WITHOUT LIMITATION, IMPLIED WARRANTIES OF MERCHANTABILITY, FITNESS FOR ANY PARTICULAR PURPOSE, OR NON INFRINGEMENT. BUYER'S EXCLUSIVE REMEDY FOR NON-CONFORMING PRODUCTS DURING THE WARRANTY PERIOD IS LIMITED TO REPAIR, REPLACEMENT OF OR REFUND FOR THE NON-CONFORMING PRODUCT(S) AT SELLER'S SOLE OPTION. THERE IS NO OBLIGATION TO REPAIR, REPLACE OR REFUND FOR PRODUCTS AS THE RESULT OF (I) ACCIDENT, DISASTER OR EVENT OF FORCE MAJEURE, (II) MISUSE, FAULT OR NEGLIGENCE OF OR BY BUYER, (III) USE OF THE PRODUCTS IN A MANNER FOR WHICH THEY WERE NOT DESIGNED, OR (IV) IMPROPER STORAGE AND HANDLING OF THE PRODUCTS. Unless otherwise expressly stated on the Product or in the documentation accompanying the Product, the Product is intended for research only and is not to be used for any other purpose, including without limitation, unauthorized commercial uses, in vitro diagnostic uses, ex vivo or in vivo therapeutic uses, or any type of consumption by or application to human or animals.

FITC anti-rat IgG2b Antibody

Catalog# / Size 408205 / 50 µg
408206 / 500 µg

Clone MRG2b-85

Regulatory Status RUO

Other Names Immunoglobulin G2b

Isotype Mouse IgG1, κ

Description The MRG2b-85 monoclonal antibody reacts with rat immunoglobulin G2b (IgG2b) in all tested rat strains (Lou, Lou/Ws1/M, Lewis, Wistar, DA, Sprague-Dawley). The MRG2b-85 monoclonal antibody may be used as primary or secondary reagent for ELISA or immunofluorescent analysis.

Product Details

Verified Reactivity Rat

Antibody Type Monoclonal

Host Species Mouse

Immunogen mixed rat Igs

Formulation Phosphate-buffered solution, pH 7.2, containing 0.09% sodium azide.

Preparation The antibody was purified by affinity chromatography, and conjugated with FITC under optimal conditions.

Concentration 0.5 mg/ml

Storage & Handling The antibody solution should be stored undiluted between 2°C and 8°C, and protected from prolonged exposure to light. **Do not freeze.**

Application FC - Quality tested

Recommended Usage Each lot of this antibody is quality control tested by immunofluorescent staining with flow cytometric analysis. For flow cytometric staining, the suggested use of this reagent is ≤1.0 µg per million cells in 100 µl volume. It is recommended that the reagent be titrated for optimal performance for each application.

Excitation Laser Blue Laser (488 nm)

Product Citations

1. Sullivan D, Watson R, Muller W 2016. Am J Physiol Heart Circ Physiol. 311: H621 - H632. PubMed

RRID AB_493001 (BioLegend Cat. No. 408205)
AB_493002 (BioLegend Cat. No. 408206)

Antigen Details

Gene ID NA

Related Protocols

- Cell Surface Flow Cytometry Staining Protocol

Other Formats

Purified anti-rat IgG2b, Biotin anti-rat IgG2b, FITC anti-rat IgG2b, Alexa Fluor® 594 anti-rat IgG2b, Alexa Fluor® 488 anti-rat IgG2b, Alexa Fluor® 647 anti-rat IgG2b, PE anti-rat IgG2b

For Research Use Only. Not for diagnostic or therapeutic use.

This product is supplied subject to the terms and conditions, including the limited license, located at www.biolegend.com/terms) ("Terms") and may be used only as provided in the Terms. Without limiting the foregoing, BioLegend products may not be used for any Commercial Purpose as defined in the Terms, resold in any form, used in manufacturing, or reverse engineered, sequenced, or otherwise studied or used to learn its design or composition without express written approval of BioLegend. Regardless of the information given in this document, user is solely responsible for determining any license requirements necessary for user’s intended use and assumes all risk and liability arising from use of the product. BioLegend is not responsible for patent infringement or any other risks or liabilities whatsoever resulting from the use of its products.

BioLegend, the BioLegend logo, and all other trademarks are property of BioLegend, Inc. or their respective owners, and all rights are reserved.

8999 BioLegend Way, San Diego, CA 92121 www.biolegend.com
Toll-Free Phone: 1-877-Bio-Legend (246-5343) Phone: (858) 768-5800 Fax: (877) 455-9587

Cell Signaling TECHNOLOGY®

Certificate of Analysis

This document certifies that this product has met all of the quality control standards defined by Cell Signaling Technology, Inc.

Research Use Only Reagent (RUO): For research use only. Not for use in diagnostic procedures.

Product Number: **98941**
Product Name: **CD8α (D4W2Z) XP® Rabbit mAb**

Product Type: Monoclonal Antibody
Species of Origin: Rabbit
Lot Number: 6
Concentration: 639 µg/ml

Approved Applications:

- IHC Leica Bond
- Immunohistochemistry (Paraffin)
- Western Blotting

Approval:	
Production: Valerie Goss	Date: 21-July-2020

3 Trask Lane, Danvers, MA 01923
www.cellsignal.com
phone: 978-867-2300
fax: 978-867-2400

この使用説明書をよく読んでから使用してください。

**2012 年 2 月改訂(第 3 版)
*2008 年 5 月改訂(第 2 版)
2005 年 5 月作成

研究用試薬　　パラフィン包埋切片用

ヒストファイン シンプルステインマウス MAX-PO(Rat)

貯　　法：2－8℃保存
包　　装：170 テスト(17mL×1 本)
有効期間：製造後 1 年 6 ヶ月**　　Code：414311

【全般的な注意】*

1. 研究用としてのみ使用すること。
2. 検体は感染の危険があるものとして取り扱いに注意すること。

【内　容】

アミノ酸ポリマーに、ペルオキシダーゼとFab'にした抗ラットIgG(動物種：ヤギ)を結合させた標識ポリマー。液状。安定化タンパク質と抗菌剤を含むMOPS(3-Morpholinopropanesulfonic acid)緩衝液(pH6.5)にて即時使用可能な濃度に調製済み。

【製　法】

1. 免疫した動物血清より精製したIgGフラクションを消化し、$F(ab')_2$を作製する。
2. 抗原を用いたアフィニティークロマトグラフィーで抗原特異的な$F(ab')_2$を精製する。
3. 固相化したマウスIgGとマウス血清タンパク質による吸収操作を行う。
4. ペルオキシダーゼとアミノ酸ポリマーを結合させ､それに$F(ab')_2$を還元して得たFab'を結合させる。

【用途及び原理】*

マウス組織用免疫組織化学染色試薬。ラット第一抗体に用いる。
酵素抗体法により、組織中の抗原を検出する。マウス組織または細胞に、まずラット第一抗体を反応させ、次に本品を反応させると、抗原・抗体・ポリマー・酵素の複合体が形成される。この複合体の酵素活性を利用して基質を発色させ、抗原部位を染色する。

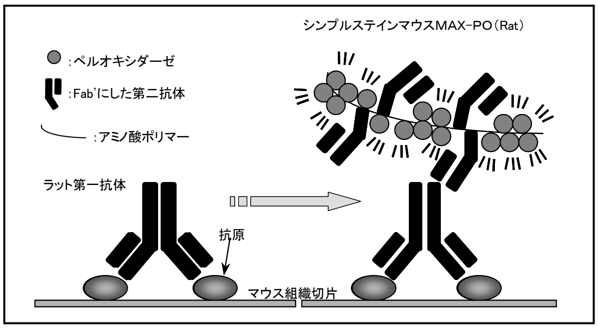

【用法・用量(操作方法)】*

○検体の準備

組織形態や抗原活性を維持した最適固定を得るため、できるだけ新鮮で小さな組織切片(約 1cm×1cm×0.5cm)の使用と、下表の固定液の使用を勧める。

固定液	固定時間
10%(緩衝)ホルマリン	24-48 時間
20%ホルマリン	12-24 時間

○切片および標本の準備

[パラフィン包埋切片]
切片を 3－6μmに薄切し、スライドに付着させる。もし、熱による抗原賦活化処理やタンパク分解酵素処理を行う場合は、0.02% poly-L-lysine あるいはシランなどの組織切片用接着剤を使用する。

[検体標本スライドの準備]
検体標本スライドとして１検体あたり、２枚準備する。
１枚は、試薬対照スライドとして、第一抗体のかわりにネガティブコントロール（ラット正常血清）を使用して染色操作を行う。

[検体対照スライドの準備]
・陽性コントロールスライド
検体標本スライドと同様の方法で作製され、あらかじめ目的抗原が存在することを確認している組織切片スライド
・陰性コントロールスライド
検体標本スライドと同様の方法で作製され、あらかじめ目的抗原が存在しないことを確認している組織切片スライド

以上の検体対照スライドを用意し、検体標本スライド及び試薬対照スライドと並行して、検体の準備から染色処理、検鏡までの全工程を行う。

○操作方法

[必要な試薬、器具]
・スライドガラス
・乾燥器　　・タイマー
・染色ドーゼ
・キシレン　　・95%エタノール　　・100%エタノール
・洗浄用容器
・PBS(「PBS Code:415223」を使用することを推奨する。または下記にて調製する。)
リン酸緩衝生理食塩水(PBS)(ｐH7.6±0.2)
NaCl　7.75g
K_2HPO_4　1.50g
KH_2PO_4　0.20g　／　精製水 1 L
・スライドスタンド　　・精製水
・湿潤箱
・3%過酸化水素加メタノール
(30%過酸化水素水をメタノールで 10 倍希釈)
・ラット第一抗体　　・ネガティブコントロール（ラット正常血清）
・基質溶液
(ヒストファイン DAB 基質キット、シンプルステイン DAB 溶液あるいはシンプルステイン AEC 溶液を使用することを推奨する。)
・対比染色試薬
・カバーガラス　　・封入剤
・ティッシュペーパー　　・光学顕微鏡
・組織切片用接着剤（0.02%poly-L-lysine、シランなど）
・抗原賦活化液(必要な場合)

[脱パラフィン]
1.キシレン処理
(1) スライドをキシレンに 3 分間浸す。
(2) 余分な液を振り払い、別のキシレンに 3 分間浸す。
(3) 余分な液を振り払い、さらに別のキシレンに 3 分間浸す。

2.エタノール処理
(1) 100%エタノールに 3 分間浸す。
(2) 余分な液を振り払い、別の 100%エタノールに 3 分間浸す。
(3) 余分な液を振り払い、95%エタノールに 3 分間浸す。
(4) 余分な液を振り払い、別の 95%エタノールに 3 分間浸す。

3.洗浄
余分な液を振り払い､PBS でよくすすぐ(3 分間ずつ容器を 2 度かえるか、または洗浄ビンを使用する)。

[染色手順]
1.ブロッキング試薬による処理（内因性ペルオキシダーゼの除去）
(1) 余分な水分を取り除くためスライド上の切片周辺を注意深く拭く。
(2) 切片が完全に覆われるようにブロッキング試薬（3%過酸化水素加メタノール）に浸し、常温(15－25℃)で 10－15 分間反応させる。
(3) PBS でよくすすぐ。(5 分間ずつ容器を 2 度かえるか、または洗浄びんを使用する。)

2.第一抗体の添加・反応
(1) スライド上の切片周辺を注意深く拭く。
(2) 切片が完全に覆われるようにラット第一抗体 2 滴を各標本スライド、陽性コントロールスライドおよび陰性コントロールスライドに滴下する。
(3) 試薬対照スライドには、ラット第一抗体のかわりにネガティブコントロール（ラット正常血清）2 滴を滴下する。
(4) 常温(15－25℃)あるいは 4℃で反応させる（各第一抗体についている添付書のインキュベーション時間を守る)。
(5) PBS でよくすすぐ。

3.シンプルステインマウス MAX-PO(Rat)の添加・反応
(1) スライド上の切片周辺を注意深く拭く。
(2) 切片が完全に覆われるようにシンプルステインマウス MAX-PO(Rat) 2 滴をすべてのスライドに滴下する。常温(15－25℃)で 30 分間反応させる。
(3) PBS でよくすすぐ。

4.基質溶液の添加・反応
(1) スライド上の切片周辺を注意深く拭く。
(2) 切片が完全に覆われるように基質溶液 2 滴を滴下する。常温(15－25℃)で 5－20 分間反応させる。
(3) 精製水でよくすすぐ。

[対比染色]
(1) 対比染色試薬にスライドを浸す。
(2) 流水洗する。

[封入]
基質溶液が AEC 発色の場合はこのまま水溶性封入剤で、DAB 発色の場合は、水洗、脱水、キシレンによる透徹後、非水溶性封入剤で封入する。

【測定結果の判定法】*

○判定方法
光学顕微鏡によって陽性反応を観察する。
染色結果の判定は、3 種類の対照スライドとの比較により行う。
- **陽性コントロールスライド**
  陽性所見が得られる。
- **陰性コントロールスライド**
  陽性を呈する細胞が認められない。
- **試薬対照スライド**
  陽性を呈する細胞が認められない。このスライドが染色されれば、非特異的なタンパク結合などによる非特異的反応が考えられる。

○判定上の留意事項
(1) 必ず各検体対照スライドの染色結果と比較して、染色結果を判定すること。
(2) 明瞭な染色を得るには、包埋剤を完全に除去することが大切である。パラフィンの残存物は、バックグラウンド染色を強める原因となる。
(3) 一般的にタンパク質や基質反応生成物の非免疫的結合により、偽陽性結果が観察される場合がある。偽陽性結果は赤血球による偽ペルオキシダーゼ反応やサイトクローム C による内因性ペルオキシダーゼ反応によっても起きることがある。
(4) 検体組織の壊死部分は、抗体が非特異的に結合しやすく、非特異染色の原因となりやすいので、陰性コントロールスライドと比較し、十分注意して判定すること。
(5) 間質系のコラーゲンは固定後疎水性となって抗体と結合しやすくなり、また、陰性に帯電しているため陽性に帯電している抗体と結合しやすく、非特異染色の原因となりやすいので、陰性コントロールスライドと比較し、十分注意して判定すること。
(6) 顆粒球の一部およびマクロファージなどは細胞膜表面に Fc レセプターを有するため、抗体の Fc 部分と結合する可能性がある。抗体本来の特異的反応部位以外に染色が現れることがあるため、必ず陰性コントロールスライドと比較し、十分注意して判定すること。

【使用上又は取り扱い上の注意】*

1.取り扱い上（危険防止）の注意
(1) 検体は、取り扱い者に感染を引き起こす危険性がある。従って、適切な取り扱いを必要とする。
(2) 皮膚などへの接触は避けること。

2.使用上の注意
(1) 試薬は 2－8℃で保存すること。
(2) 使用前に常温(15－25℃)に戻して使用すること。
(3) 有効期間の過ぎた試薬は使用しないこと。
(4) 染色過程のいかなる時点においても切片を乾燥させてはならない。試薬と反応させている間、切片を湿潤箱に入れておくと乾燥を防ぐことができる。
(5) 抗原は熱に弱いので、組織を包埋する際に、パラフィンの温度を 58℃以上に上げてはならない。
(6) 脱パラフィンに用いるキシレンおよびエタノールは、スライドを 40 枚処理するごとに取り替える。
(7) ステロイドやその他小さな分子は、有機溶媒に極めて溶けやすく、抗原の損失を防ぐには、固定剤の選択に注意する必要がある。

3.廃棄上の注意
(1) 検体組織に接触した器具・試薬および試薬容器等は感染の危険性があるので、オートクレーブで 120℃、20 分間滅菌処理するか、または 1.0V/V%次亜塩素酸などの消毒液に浸して一晩処理すること。

妨害物質と問題対策

問題点	考えられる原因	対策
○陽性コントロールスライド及び標本スライドの染色が認められない、あるいは弱い。	①切片が乾燥している。	①切片を湿潤させた後は、湿潤箱などを用いて乾燥させない。
	②包埋剤が不適当あるいはパラフィン包埋組織からのパラフィン除去が不完全である。	②適当な包埋剤を選択する。また、包埋組織から、パラフィンを完全に除去する。<br>②キシレン、エタノール溶液を取り替える。
	③緩衝液中の微量のアジ化ナトリウムがペルオキシダーゼを不活性化し、染色を不可能にする。	③アジ化ナトリウムを含有しない緩衝液を使用する。<br>③緩衝液を取り替える。
	④酵素や抗体反応が不十分。	④古い基質溶液を取り替える。<br>④各ステップでの水分の拭き取りを完全にする。<br>④抗体との反応時間を十分にする。特に、第一抗体では添付書のインキュベーション時間を守る。
○陽性コントロールスライドは染色されるが、標本スライドは染色されない。	①抗原が固定あるいは包埋過程で変性したり、マスクされている。	①抗原の中には、固定や包埋に敏感なものがあるので、穏やかな固定剤を使用し固定時間を短縮する。<br>①場合によっては、染色前に抗原を露出させるため、熱による抗原賦活化処理あるいはトリプシンなどのタンパク分解酵素処理を行う。
	②自己消化により抗原が破壊されている。	②採取した組織はすみやかに適切な方法で固定を行うこと。
	③組織に存在する抗原が少ない。	③インキュベーション時間を長く設定する。
○全ての染色スライドのバックグラウンドが強く染色される。	①内因性ペルオキシダーゼを不活性化するための処理が不十分。	①ブロッキング試薬（3%過酸化水素加メタノール）による処理を確実に行う。
	②非特異結合成分がある。	②第一抗体の添加前に 10%ヤギ正常血清で処理する。
	③自己消化の結果、組織液に遊離した抗原が過剰に存在する。	③可能ならば、新鮮な組織を包埋する。
	④不完全なパラフィン除去。	④キシレン、エタノール溶液を取り替える。
	⑤不十分な抗体の洗浄。	⑤抗体の洗浄を十分に行う。
	⑥室内温度が高すぎて、酵素反応が早すぎる。	⑥常温（15－25℃）にコントロールする。<br>⑥反応時間を短縮する。
	⑦切片が乾燥している。	⑦切片を湿潤させた後は、湿潤箱などを用いて乾燥させない。
○反応中に組織切片がスライドからはがれてしまう。	①抗原によってはその同定のために、熱による抗原賦活化処理あるいは第一抗体との長時間の反応を必要とする。このような場合には、はがれ易くなる。	①0.02%poly-L-lysine、シランなどの組織切片用接着剤を使用する。

【貯蔵方法・有効期間】**

貯蔵方法：2－8℃保存
有効期間：製造後 1 年 6 ヶ月**

【包装単位】*

製品名	コード	包装単位
ヒストファイン　シンプルステインマウス MAX-PO(Rat)	414311	170 テスト (17mL×1 本)

上記キットと組み合わせて使用することを推奨する。

製品名	コード	包装単位
DAB 基質キット	425011	500 テスト
発色基質（試薬 A）<br>基質緩衝液（試薬 B）<br>発色試薬（試薬 C）		3mL×1 本<br>3mL×1 本<br>3mL×1 本

【問合せ先、製造販売元、販売元】

株式会社ニチレイバイオサイエンス

〒104-8402　東京都中央区築地 6-19-20
TEL：03-3248-2208　　FAX：03-3248-2243

*■参考(凍結切片を用いて染色を行う場合)

本品は、凍結切片を用いた免疫組織化学染色法に適用する場合、以下の注意事項、操作方法を参考にすること。

通常の染色手順においてバックグラウンド染色が認められる場合、下記のステップにて調整することを推奨する。

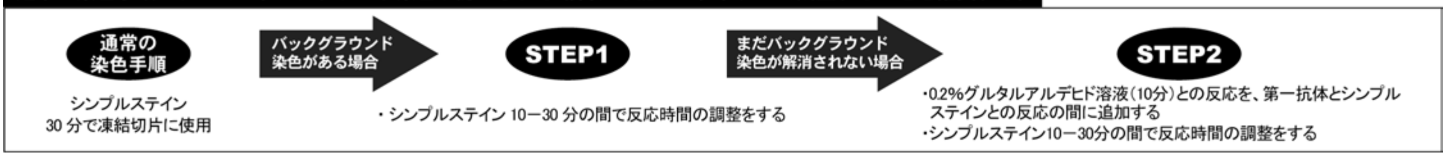

【凍結切片を用いて染色を行う場合の注意】

1. 凍結切片の作製方法について
   組織は、固定組織、未固定組織いずれも使用できる。
   注）固定組織：固定後、凍結・薄切された組織
   　　未固定組織：凍結・薄切後に組織を固定させた組織(新鮮凍結組織)
2. 固定液について
   第一抗体により適する固定液は異なるので、固定液の検討は十分に行うこと。
3. シンプルステインマウス MAX-PO(Rat)の反応時間について
   シンプルステインマウス MAX-PO(Rat)は、パラフィン包埋切片用に濃度、反応時間(30分)を設定している。凍結切片で、本製品を30分間反応させ、バックグラウンド染色(非特異染色)が認められた場合には、本製品の反応時間を10－30分の間で短くすること(STEP1)。マウスの系統・組織・固定方法により、至適時間が異なるので、十分検討を行うこと。
4. 0.2%グルタルアルデヒド溶液の使用について
   シンプルステインマウス MAX-PO(Rat)の反応時間を短縮しても、バックグラウンド染色(非特異染色)が認められる場合、0.2%グルタルアルデヒド溶液でのブロッキングにより、バックグラウンド染色(非特異染色)が低下する場合がある(STEP2)。0.2%グルタルアルデヒド溶液の作製方法・使用方法については、下記(○操作方法)を参照すること。なお、0.2%グルタルアルデヒド溶液を併用する場合、使用する第一抗体の反応を阻害しないかどうかの確認を行うこと。また、マウスの系統・組織・固定方法により、効果に差があるので、十分確認の上、使用すること。

【用法・用量(操作方法)】

○検体の準備

[凍結切片]

不安定な抗原の場合は、凍結切片標本を用いる。
4%パラホルムアルデヒドなどの固定液を用いて固定した組織または未固定(新鮮)組織は O.C.T. コンパウンドあるいは類似の包埋剤とともに、液体窒素あるいはドライアイス－アセトン、ドライアイス－エタノールなどで急速凍結する。

[固定組織(固定後、凍結・薄切した組織)を用いる場合]

凍結した切片を 4－6μmに薄切し、あらかじめ 0.02%poly-L-lysine などの組織切片用接着剤で被膜し空気乾燥したスライドに付着させ、十分に乾燥後、水溶性の凍結用包埋剤を取り除くため、PBS でよくすすぐ。

[未固定組織(凍結・薄切後に組織を固定する組織)を用いる場合]

凍結した切片を 4－6μmに薄切し、あらかじめ 0.02%poly-L-lysine などの組織切片用接着剤で被膜し空気乾燥したスライドに付着させ、十分に乾燥後、水溶性の凍結用包埋剤を取り除くため、PBS でよくすすぐ。
その後、4%パラホルムアルデヒド、アセトン、エタノールなどで 4℃10 分間、固定し、PBS でよくすすぐ。

○操作方法

[必要な試薬、器具]

・P1 記載内容参照のこと。
・0.2%グルタルアルデヒド溶液(必要な場合)
　50%グルタルアルデヒド(SIGMA 社：コード G7651)を PBS で 0.2%となるように 250 倍希釈。

[染色手順]

1.ブロッキング試薬による処理（内因性ペルオキシダーゼの除去）
　注)内因性ペルオキシダーゼあるいは赤血球や顆粒球の含量が多くない場合は
　　内因性ペルオキシダーゼの不活性化の処理ステップを省略できる。
(1) スライド上の切片周辺を注意深く拭く。
(2) 切片が完全に覆われるようにブロッキング試薬（3%過酸化水素加メタノール）に浸し、常温(15－25℃)で 10－15 分間反応させる。
(3) PBS でよくすすぐ。（5 分間ずつ容器を 2 度かえるか、または洗浄びんを使用する。）

2.第一抗体の添加・反応
(1) スライド上の切片周辺を注意深く拭く。
(2) 切片が完全に覆われるようにラット第一抗体 2 滴を各標本スライド、陽性コントロールスライドおよび陰性コントロールスライドに滴下する。
(3) 試薬対照スライドには、ラット第一抗体のかわりにネガティブコントロール（ラット正常血清）2 滴を滴下する。
(4) 常温(15－25℃)あるいは 4℃で反応させる(各第一抗体についている添付書のインキュベーション時間を守る)。
(5) PBS でよくすすぐ。

3. 0.2%グルタルアルデヒド溶液の添加・反応(STEP2)
(1) スライド上の切片周辺を注意深く拭く。
(2) 切片が完全に覆われるように 0.2%グルタルアルデヒド溶液 2 滴をすべてのスライドに滴下する。常温(15－25℃)で 10 分間反応させる。
(3) PBS でよくすすぐ。

4 .シンプルステインマウス MAX-PO(Rat)の添加・反応
(1) スライド上の切片周辺を注意深く拭く。
(2) 切片が完全に覆われるようにシンプルステインマウス MAX-PO(Rat) 2 滴をすべてのスライドに滴下する。常温(15－25℃)で 30 分間反応させる。バックグラウンド染色が認められる場合には 10－30 分の間で短くすること(STEP1)。
(3) PBS でよくすすぐ。

5.基質溶液の添加・反応
(1) スライド上の切片周辺を注意深く拭く。
(2) 切片が完全に覆われるように基質溶液 2 滴を滴下する。常温(15－25℃)で 5－20 分間反応させる。
(3) 精製水でよくすすぐ。

[対比染色]

(1) 対比染色試薬にスライドを浸す。
(2) 流水洗する。

[封入]

基質溶液が AEC 発色の場合はこのまま水溶性封入剤で、DAB 発色の場合は、水洗、脱水、キシレンによる透徹後、非水溶性封入剤で封入する。

検体標本スライドの準備、検体対照スライドの準備、測定結果の判定法、使用上又は取り扱い上の注意等は、P1、P2 記載内容を参照のこと。

操作方法

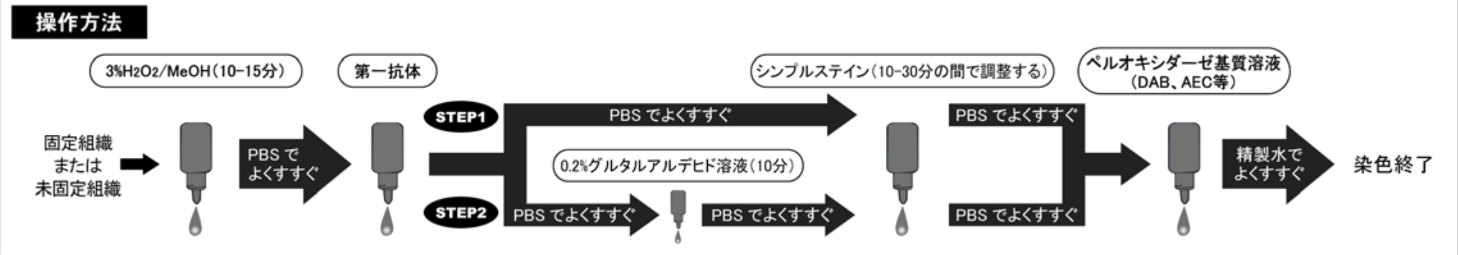

この使用説明書をよく読んでから使用してください。

** 2008 年　5 月改訂(第 3 版)
* 2006 年 12 月改訂(第 2 版)
2005 年　4 月作成

研究用試薬　　パラフィン包埋切片用

ヒストファイン　シンプルステインマウス MAX-PO(R)

貯　　法 ：2－8℃保存
包　　装 ：170 テスト(17mL×1 本)
有効期間 ：製造後 1 年 6 ヶ月*　　Code：414341

【全般的な注意】**

1. 研究用としてのみ使用すること。
2. 検体は感染の危険があるものとして取り扱いに注意すること。

【内　容】

アミノ酸ポリマーに、ペルオキシダーゼとFab'にした抗ウサギIgG(動物種：ヤギ)を結合させた標識ポリマー。液状。安定化タンパク質と抗菌剤を含むMOPS(3-Morpholinopropanesulfonic acid)緩衝液(pH6.5)にて即時使用可能な濃度に調製済み。

【製　法】

1. 免疫した動物血清より精製したIgGフラクションを消化し、$F(ab')_2$を作製する。
2. 抗原を用いたアフィニティークロマトグラフィーで抗原特異的な$F(ab')_2$を精製する。
3. 固相化したヒト血清タンパク質、マウスIgGとマウス血清タンパク質による吸収操作を行う。
4. ペルオキシダーゼとアミノ酸ポリマーを結合させ、それに$F(ab')_2$を還元して得たFab'を結合させる。

【用途及び原理】**

マウス組織用免疫組織化学染色試薬。ウサギ第一抗体に用いる。
酵素抗体法により、組織中の抗原を検出する。マウス組織または細胞に、まずウサギ第一抗体を反応させ、次に本品を反応させると、抗原・抗体・ポリマー・酵素の複合体が形成される。この複合体の酵素活性を利用して基質を発色させ、抗原部位を染色する。

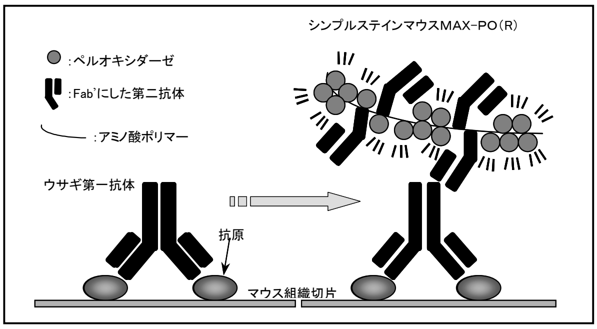

【用法・用量(操作方法)】**

○検体の準備

組織形態や抗原活性を維持した最適固定を得るため、できるだけ新鮮で小さな組織切片(約 1cm×1cm×0.5cm)の使用と、下表の固定液の使用を勧める。

固定液	固定時間
10%(緩衝)ホルマリン	24-48 時間
20%ホルマリン	12-24 時間

○切片および標本の準備

[パラフィン包埋切片]

切片を 3－6μmに薄切し、スライドに付着させる。もし、熱による抗原賦活化処理やタンパク分解酵素処理を行う場合は、0.02% poly-L-lysine あるいはシランなどの組織切片用接着剤を使用する。

[検体標本スライドの準備]

検体標本スライドとして 1 検体あたり、2 枚準備する。
1 枚は、試薬対照スライドとして、第一抗体のかわりにネガティブコントロール（ウサギ正常血清）を使用して染色操作を行う。

[検体対照スライドの準備]

・陽性コントロールスライド
検体標本スライドと同様の方法で作製され、あらかじめ目的抗原が存在することを確認している組織切片スライド
・陰性コントロールスライド
検体標本スライドと同様の方法で作製され、あらかじめ目的抗原が存在しないことを確認している組織切片スライド

以上の検体対照スライドを用意し、検体標本スライド及び試薬対照スライドと並行して、検体の準備から染色処理、検鏡までの全工程を行う。

○操作方法

[必要な試薬、器具]

・スライドガラス
・乾燥器　・タイマー
・染色ドーゼ
・キシレン　・95%エタノール　・100%エタノール
・洗浄用容器
・PBS(「PBS Code:415223」を使用することを推奨する。または下記にて調製する。)
リン酸緩衝生理食塩水(PBS)(ｐH7.6±0.2)
NaCl　7.75g
K_2HPO_4　1.50g
KH_2PO_4　0.20g　／　精製水 1 L
・スライドスタンド　・精製水　・湿潤箱
・3%過酸化水素加メタノール
(30%過酸化水素水をメタノールで 10 倍希釈)
・ウサギ第一抗体　・ネガティブコントロール（ウサギ正常血清）
・基質溶液
(ヒストファイン DAB 基質キット、シンプルステイン DAB 溶液あるいはシンプルステイン AEC 溶液を使用することを推奨する。)
・対比染色試薬
・カバーガラス　・封入剤
・ティッシュペーパー　・光学顕微鏡
・組織切片用接着剤（0.02%poly-L-lysine、シランなど）
・抗原賦活化液(必要な場合)

[脱パラフィン]

1.キシレン処理
(1) スライドをキシレンに 3 分間浸す。
(2) 余分な液を振り払い、別のキシレンに 3 分間浸す。
(3) 余分な液を振り払い、さらに別のキシレンに 3 分間浸す。

2.エタノール処理
(1) 100%エタノールに 3 分間浸す。
(2) 余分な液を振り払い、別の 100%エタノールに 3 分間浸す。
(3) 余分な液を振り払い、95%エタノールに 3 分間浸す。
(4) 余分な液を振り払い、別の 95%エタノールに 3 分間浸す。

3.洗浄
余分な液を振り払い、PBS でよくすすぐ(3 分間ずつ容器を 2 度かえるか、または洗浄ビンを使用する)。

[染色手順]
1.ブロッキング試薬による処理（内因性ペルオキシダーゼの除去）
(1) 余分な水分を取り除くためスライド上の切片周辺を注意深く拭く。
(2) 切片が完全に覆われるようにブロッキング試薬（3%過酸化水素加メタノール）に浸し、常温(15－25℃)で 10－15 分間反応させる。
(3) PBS でよくすすぐ。（5 分間ずつ容器を 2 度かえるか、または洗浄びんを使用する。）

2.第一抗体の添加・反応
(1) スライド上の切片周辺を注意深く拭く。
(2) 切片が完全に覆われるようにウサギ第一抗体 2 滴を各標本スライド、陽性コントロールスライドおよび陰性コントロールスライドに滴下する。
(3) 試薬対照スライドには、ウサギ第一抗体のかわりにネガティブコントロール（ウサギ正常血清）2 滴を滴下する。
(4) 常温(15－25℃)あるいは 4℃で反応させる（各第一抗体についている添付書のインキュベーション時間を守る）。
(5) PBS でよくすすぐ。

3.シンプルステインマウス MAX-PO(R)の添加・反応
(1) スライド上の切片周辺を注意深く拭く。
(2) 切片が完全に覆われるようにシンプルステインマウス MAX-PO(R) 2 滴をすべてのスライドに滴下する。常温(15－25℃)で 30 分間反応させる。
(3) PBS でよくすすぐ。

4.基質溶液の添加・反応
(1) スライド上の切片周辺を注意深く拭く。
(2) 切片が完全に覆われるように基質溶液 2 滴を滴下する。常温(15－25℃)で 5－20 分間反応させる。
(3) 精製水でよくすすぐ。

[対比染色]
(1) 対比染色試薬にスライドを浸す。
(2) 流水洗する。

[封入]
基質溶液が AEC 発色の場合はこのまま水溶性封入剤で、DAB 発色の場合は、水洗、脱水、キシレンによる透徹後、非水溶性封入剤で封入する。

【測定結果の判定法】**

○判定方法
光学顕微鏡によって陽性反応を観察する。
染色結果の判定は、3 種類の対照スライドとの比較により行う。
- **陽性コントロールスライド**
  陽性所見が得られる。
- **陰性コントロールスライド**
  陽性を呈する細胞が認められない。
- **試薬対照スライド**
  陽性を呈する細胞が認められない。このスライドが染色されれば、非特異的なタンパク結合などによる非特異的反応が考えられる。

○判定上の留意事項
(1) 必ず各検体対照スライドの染色結果と比較して、染色結果を判定すること。
(2) 明瞭な染色を得るには、包埋剤を完全に除去することが大切である。パラフィンの残存物は、バックグラウンド染色を強める原因となる。
(3) 一般的にタンパク質や基質反応生成物の非免疫的結合により、偽陽性結果が観察される場合がある。偽陽性結果は赤血球による偽ペルオキシダーゼ反応やサイトクローム C による内因性ペルオキシダーゼ反応によっても起きることがある。
(4) 検体組織の壊死部分は、抗体が非特異的に結合しやすく、非特異染色の原因となりやすいので、陰性コントロールスライドと比較し、十分注意して判定すること。
(5) 間質系のコラーゲンは固定後疎水性となって抗体と結合しやすくなり、また、陰性に帯電しているため陽性に帯電している抗体と結合しやすく、非特異染色の原因となりやすいので、陰性コントロールスライドと比較し、十分注意して判定すること。
(6) 顆粒球の一部およびマクロファージなどは細胞膜表面に Fc レセプターを有するため、抗体の Fc 部分と結合する可能性がある。抗体本来の特異的反応部位以外に染色が現れることがあるため、必ず陰性コントロールスライドと比較し、十分注意して判定すること。

【使用上又は取り扱い上の注意】**

1.取り扱い上（危険防止）の注意
(1) 検体は、取り扱い者に感染を引き起こす危険性がある。従って、適切な取り扱いを必要とする。
(2) 皮膚などへの接触は避けること。

2.使用上の注意
(1) 試薬は 2－8℃で保存すること。
(2) 使用前に常温(15－25℃)に戻して使用すること。
(3) 有効期間の過ぎた試薬は使用しないこと。
(4) 染色過程のいかなる時点においても切片を乾燥させてはならない。試薬と反応させている間、切片を湿潤箱に入れておくと乾燥を防ぐことができる。
(5) 抗原は熱に弱いので、組織を包埋する際に、パラフィンの温度を 58℃以上に上げてはならない。
(6) 脱パラフィンに用いるキシレンおよびエタノールは、スライドを 40 枚処理するごとに取り替える。
(7) ステロイドやその他小さな分子は、有機溶媒に極めて溶けやすく、抗原の損失を防ぐには、固定剤の選択に注意する必要がある。

3.廃棄上の注意
(1) 検体組織に接触した器具・試薬および試薬容器等は感染の危険性があるので、オートクレーブで 120℃、20 分間滅菌処理するか、または 1.0V/V%次亜塩素酸などの消毒液に浸して一晩処理すること。

妨害物質と問題対策

問題点	考えられる原因	対策
○陽性コントロールスライド及び標本スライドの染色が認められない、あるいは弱い。	①切片が乾燥している。	①切片を湿潤させた後は、湿潤箱などを用いて乾燥させない。
	②包埋剤が不適当あるいはパラフィン包埋組織からのパラフィン除去が不完全である。	②適当な包埋剤を選択する。また、包埋組織から、パラフィンを完全に除去する。<br>②キシレン、エタノール溶液を取り替える。
	③緩衝液中の微量のアジ化ナトリウムがペルオキシダーゼを不活性化し、染色を不可能にする。	③アジ化ナトリウムを含有しない緩衝液を使用する。<br>③緩衝液を取り替える。
	④酵素や抗体反応が不十分。	④古い基質溶液を取り替える。<br>④各ステップでの水分の拭き取りを完全にする。<br>④抗体との反応時間を十分にする。特に、第一抗体では添付書のインキュベーション時間を守る。
○陽性コントロールスライドは染色されるが、標本スライドは染色されない。	①抗原が固定あるいは包埋過程で変性したり、マスクされている。	①抗原の中には、固定や包埋に敏感なものがあるので、穏やかな固定剤を使用し固定時間を短縮する。<br>①場合によっては、染色前に抗原を露出させるため、熱による抗原賦活化処理あるいはトリプシンなどのタンパク分解酵素処理を行う。
	②自己消化により抗原が破壊されている。	②採取した組織はすみやかに適切な方法で固定を行うこと。
	③組織に存在する抗原が少ない。	③インキュベーション時間を長く設定する。
○全ての染色スライドのバックグラウンドが強く染色される。	①内因性ペルオキシダーゼを不活性化するための処理が不十分。	①ブロッキング試薬（3%過酸化水素加メタノール）による処理を確実に行う。
	②非特異結合成分がある。	②第一抗体の添加前に 10%ヤギ正常血清で処理する。
	③自己消化の結果、組織液に遊離した抗原が過剰に存在する。	③可能ならば、新鮮な組織を包埋する。
	④不完全なパラフィン除去。	④キシレン、エタノール溶液を取り替える。
	⑤不十分な抗体の洗浄。	⑤抗体の洗浄を十分に行う。
	⑥室内温度が高すぎて、酵素反応が早すぎる。	⑥常温（15－25℃）にコントロールする。<br>⑥反応時間を短縮する。
	⑦切片が乾燥している。	⑦切片を湿潤させた後は、湿潤箱などを用いて乾燥させない。
○反応中に組織切片がスライドからはがれてしまう。	①抗原によってはその固定のために、熱による抗原賦活化処理あるいは第一抗体との長時間の反応を必要とする。このような場合には、はがれ易くなる。	①0.02%poly-L-lysine、シランなどの組織切片用接着剤を使用する。

【貯蔵方法・有効期間】
貯蔵方法：2－8℃保存
有効期間：製造後 1 年 6 ヶ月*

【包装単位】**

製品名	コード	包装単位
ヒストファイン　シンプルステインマウス<br>MAX-PO(R)	414341	170 テスト<br>(17mL×1 本)

上記キットと組み合わせて使用することを推奨する。

製品名	コード	包装単位
DAB 基質キット	425011	500 テスト
発色基質（試薬 A）<br>基質緩衝液（試薬 B）<br>発色試薬（試薬 C）		3mL×1 本<br>3mL×1 本<br>3mL×1 本

【問合せ先、製造販売元、販売元】

株式会社ニチレイバイオサイエンス
〒104-8402　東京都中央区築地 6-19-20
TEL：03-3248-2208　　FAX：03-3248-2243

**■参考(凍結切片を用いて染色を行う場合)

本品は、凍結切片を用いた免疫組織化学染色法に適用する場合、以下の注意事項、操作方法を参考にすること。

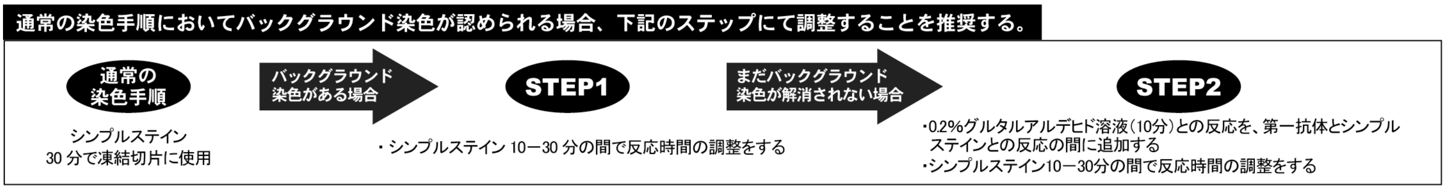

【凍結切片を用いて染色を行う場合の注意】

1. 凍結切片の作製方法について
   組織は、固定組織、未固定組織いずれも使用できる。
   注）固定組織：固定後、凍結・薄切された組織
   未固定組織：凍結・薄切後に組織を固定させた組織(新鮮凍結組織)
2. 固定液について
   第一抗体により適する固定液は異なるので、固定液の検討は十分に行うこと。
3. シンプルステインマウス MAX-PO(R)の反応時間について
   シンプルステインマウス MAX-PO(R)は、パラフィン包埋切片用に濃度、反応時間(30分)を設定している。凍結切片で、本製品を30分間反応させ、バックグラウンド染色(非特異染色)が認められた場合には、本製品の反応時間を10－30分の間で短くすること(STEP1)。マウスの系統・組織・固定方法により、至適時間が異なるので、十分検討を行うこと。
4. 0.2%グルタルアルデヒド溶液の使用について
   シンプルステインマウス MAX-PO(R)の反応時間を短縮しても、バックグラウンド染色(非特異染色)が認められる場合、0.2%グルタルアルデヒド溶液でのブロッキングにより、バックグラウンド染色(非特異染色)が低下する場合がある(STEP2)。0.2%グルタルアルデヒド溶液の作製方法・使用方法については、下記(○操作方法)を参照すること。なお、0.2%グルタルアルデヒド溶液を併用する場合、使用する第一抗体の反応を阻害しないかどうかの確認を行うこと。また、マウスの系統・組織・固定方法により、効果に差があるので、十分確認の上、使用すること。

【用法・用量(操作方法)】

○検体の準備

[凍結切片]

不安定な抗原の場合は、凍結切片標本を用いる。
4%パラホルムアルデヒドなどの固定液を用いて固定した組織または未固定(新鮮)組織は O.C.T. コンパウンドあるいは類似の包埋剤とともに、液体窒素あるいはドライアイス－アセトン、ドライアイス－エタノールなどで急速凍結する。

[固定組織(固定後、凍結・薄切した組織)を用いる場合]

凍結した切片を 4－6 μ m に薄切し、あらかじめ 0.02%poly-L-lysine などの組織切片用接着剤で被膜し空気乾燥したスライドに付着させ、十分に乾燥後、水溶性の凍結用包埋剤を取り除くため、PBS でよくすすぐ。

[未固定組織(凍結・薄切後に組織を固定する組織)を用いる場合]

凍結した切片を 4－6 μ m に薄切し、あらかじめ 0.02%poly-L-lysine などの組織切片用接着剤で被膜し空気乾燥したスライドに付着させ、十分に乾燥後、水溶性の凍結用包埋剤を取り除くため、PBS でよくすすぐ。
その後、4%パラホルムアルデヒド、アセトン、エタノールなどで 4℃10 分間、固定し、PBS でよくすすぐ。

○操作方法

[必要な試薬、器具]

・P1 記載内容参照のこと。
・0.2%グルタルアルデヒド溶液(必要な場合)
  50%グルタルアルデヒド(SIGMA 社：コード G7651)を PBS で 0.2%となるように 250 倍希釈。

[染色手順]

1.ブロッキング試薬による処理（内因性ペルオキシダーゼの除去）
注)内因性ペルオキシダーゼあるいは赤血球や顆粒球の含量が多くない場合は内因性ペルオキシダーゼの不活性化の処理ステップを省略できる。
(1) スライド上の切片周辺を注意深く拭く。
(2) 切片が完全に覆われるようにブロッキング試薬（3%過酸化水素加メタノール）に浸し、常温(15－25℃)で 10－15 分間反応させる。
(3) PBS でよくすすぐ。（5 分間ずつ容器を 2 度かえるか、または洗浄びんを使用する。）

2.第一抗体の添加・反応
(1) スライド上の切片周辺を注意深く拭く。
(2) 切片が完全に覆われるようにウサギ第一抗体 2 滴を各標本スライド、陽性コントロールスライドおよび陰性コントロールスライドに滴下する。
(3) 試薬対照スライドには、ウサギ第一抗体のかわりにネガティブコントロール（ウサギ正常血清）2 滴を滴下する。
(4) 常温(15－25℃)あるいは 4℃で反応させる（各第一抗体についている添付書のインキュベーション時間を守る）。
(5) PBS でよくすすぐ。

3. 0.2%グルタルアルデヒド溶液の添加・反応(STEP2)
(1) スライド上の切片周辺を注意深く拭く。
(2) 切片が完全に覆われるように 0.2%グルタルアルデヒド溶液 2 滴をすべてのスライドに滴下する。常温(15－25℃)で 10 分間反応させる。
(3) PBS でよくすすぐ。

4 .シンプルステインマウス MAX-PO(R)の添加・反応
(1) スライド上の切片周辺を注意深く拭く。
(2) 切片が完全に覆われるようにシンプルステインマウス MAX-PO(R) 2 滴をすべてのスライドに滴下する。常温(15－25℃)で 30 分間反応させる。バックグラウンド染色が認められる場合には 10－30 分の間で短くすること(STEP1)。
(3) PBS でよくすすぐ。

5.基質溶液の添加・反応
(1) スライド上の切片周辺を注意深く拭く。
(2) 切片が完全に覆われるように基質溶液 2 滴を滴下する。常温(15－25℃)で 5－20 分間反応させる。
(3) 精製水でよくすすぐ。

[対比染色]

(1) 対比染色試薬にスライドを浸す。
(2) 流水洗する。

[封入]

基質溶液が AEC 発色の場合はこのまま水溶性封入剤で、DAB 発色の場合は、水洗、脱水、キシレンによる透徹後、非水溶性封入剤で封入する。

検体標本スライドの準備、検体対照スライドの準備、測定結果の判定法、使用上又は取り扱い上の注意等は、P1、P2 記載内容を参照のこと。

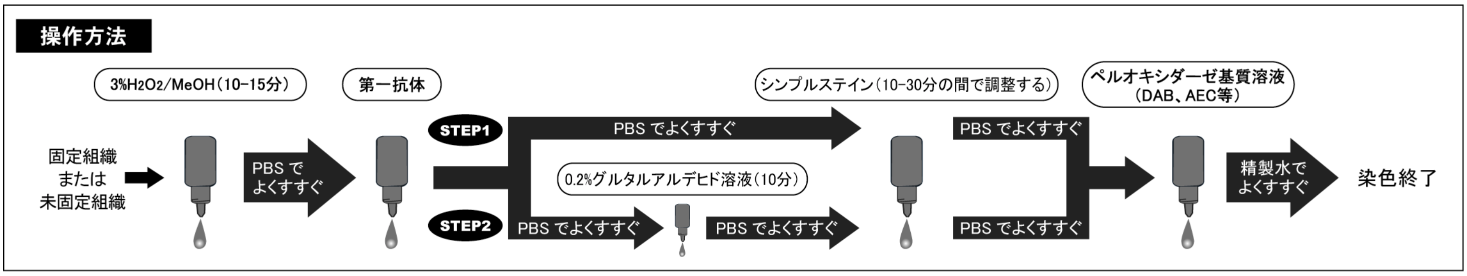

Thermo Fisher SCIENTIFIC

Certificate of Analysis

NuPAGE® Novex 4-12% Bis-Tris gel

Product No.	NP032
Lot No.	21072670
Date of Manufacture	26Jul2021
Expiration Date	24Nov2022

This certificate of analysis applies to the following catalog numbers:

Catalog No.	Description	Pack Size	Catalog No.	Description	Pack Size
NP0321BOX	1.0mm 10-well	10 gels/box	NP0324BOX	1.0mm 1-well	10 gels/box
NP0321PK2	1.0mm 10-well	2 gels/box	NP0326BOX	1.0mm 2D-well	10 gels/box
NP0322BOX	1.0mm 12-well	10 gels/box	NP0327BOX	1.0mm 9-well	10 gels/box
NP0322PK2	1.0mm 12-well	2 gels/box	NP0329BOX	1.0mm 17-well	10 gels/box
NP0323BOX	1.0mm 15-well	10 gels/box	NP0329PK2	1.0mm 17-well	2 gels/box
NP0323PK2	1.0mm 15-well	2 gels/box			

Testing Conditions
Unstained protein markers were loaded, then the gels were electrophoresed at 200V until the dye front reached the bottom of the gel. Gels were run using NuPAGE® MES SDS Running Buffer under reducing conditions.

Migration
With MES Running Buffer, reduced Myosin migrated to 0.140 ± 0.050 Rf, reduced LDH to 0.510 ± 0.050 Rf, and reduced Aprotinin to 0.820 ± 0.050 Rf.

Straightness
Across the gel, the migration of a given protein did not vary more than 3% of the length of the gel. For 17-well gels. lanes 2 and 16 are the outermost lanes tested.

Curvature
In each of the two outermost lanes, the migration of a given protein did not vary more than 2% of the length of the gel. For 17-well gels. lanes 2 and 16 are the outermost lanes tested.

Appearance

Gels run were free of swirls, bubbles, and debris. Bands were sharp and flat.

Overall Result
Meets Specification

For Research Use Only. Not for use in diagnostic procedures. If you have any further questions about this Certificate of Analysis, please contact Technical Services at 1-800-955-6288 (US and Canada) or 1-760-603-7200, x2 (all other countries).

Thermo Fisher Scientific
Life Sciences Solutions
5781 Van Allen Way
Carlsbad, CA, USA 92008
www.thermofisher.com
For inquiries, contact us at cofarequests@thermofisher.com

Shannon Orr
Sr. Manager, Quality
Issued on 27 Jul 2021

abcam

Product datasheet

InstantBlue® Coomassie Protein Stain (ISB1L) ab119211

★★★★★ 4 Abreviews 492 References 画像数 3

製品の概要

**製品名**	InstantBlue® Coomassie Protein Stain (ISB1L)
**特記事項**	InstantBlue® is a ready to use Coomassie protein stain for polyacrylamide gels. Its unique mechanism of action stains proteins in 15 minutes, while leaving a clear background eliminating the need to fix, wash or destain. Formulated for safe use and easy disposal, it's ready to use straight out of the bottle and comes in convenient premixed one liter bottles.

Why InstantBlue® Protein Stain?

There is no need to wash, fix, microwave or destain gels when using InstantBlue™. Homemade stains and premade solutions require several or all of these extra steps. InstantBlue uses a simple one step protocol.

The unique patented mechanism for rapid Coomassie blue staining of protein gels begins in moments, and results are achieved within 15 minutes. Some premade and traditional homemade Coomassie R-250 protein stains can take three hours or more to fully stain gels, and then require destaining typically overnight.

Unparalleled sensitivity allows protein levels as low as 5ng per band to be detected (BSA) when staining overnight. This is thanks to InstantBlue's protein specific staining, which gives a clear background, improving the signal / noise ratio of the stain. Despite the staining power, gels can be left in InstantBlue for weeks and still come out with a crystal clear background, all without the need for destaining or protein stain remover.

The nontoxic formula is sink disposable and thanks to its methanol free composition will not shrink gels. Other stains require solvent disposal procedures and can require microwaving, which produces dangerous fumes. The effective staining and safe composition of InstantBlue means that there's no need for microwaving, solvent disposal, or fume hoods.

InstantBlue will stain at least 40 mini gels per bottle, boasting economical staining with only 25ml per mini gel. As a result, this allows you to use less protein staining solution when compared to 50–100ml of stain per gel for homemade solutions and some premixed solutions.

Fully compatible with mass spectrometry, gels can be destained using standard procedures. The protein staining solution's acetic acid free formulation means it will not methylate or acetylate protein during the protein staining protocol.

Key Benefits

Ultra fast staining – Results in 15 minutes or less
One step procedure – No washing, fixing, microwaving or destaining
High sensitivity – 5ng bands detectable
Clear background – High signal / noise ratio

Flexible – No overstaining
Reliable – Stringent batch to batch consistency
Efficient – Only 25ml per gel
Safe composition – Nontoxic; no fume hood or solvent disposal required
Methanol free – No gel shrinkage or protein methylation
Acetic acid free – No protein acetylation
Mass spectrometry compatible – Destainable. No residual methylation or acetylation
This product is manufactured by Expedeon, an Abcam company. Expedeon product code ISB1L was previously called InstantBlue Protein Stain 1 L and is the same as the 1000 ml size of this product.

Manufactured under patent numbers : US 12/226,797 ; SG 201103010-3 ; SG 200807953-5 ; JP 2009-507182 ; IN 5722/CHENP/2008 ; GB 0608377.8 ; EP 10187154.9; EP 07733647.7 ; CN 200780021434.8 ; CA 2,649,835 ; AU 2007245443

INSTANTBLUE is a registered trademark of Abcam in the US and an unregistered trademark elsewhere

製品の特性

**製品の状態**	Liquid
**保存方法**	Shipped at Room Temperature. Store at +4°C.
**バッファー**	Constituents: 0.56% Ethanol, 15.19% Phosphoric acid

画像

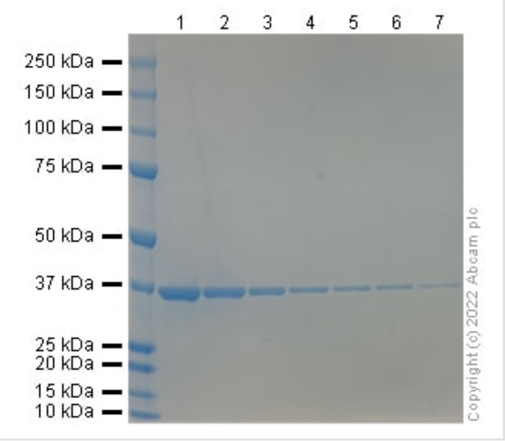

SDS-PAGE - InstantBlue® Coomassie Protein Stain (ISB1L) (ab119211)

Lane 1: Recombinant Human GAPDH protein (**ab77109**) at 3 ug + Instant Blue stain (ab119211)

Lane 2: Recombinant Human GAPDH protein (**ab77109**) at 2 ug + Instant Blue stain (ab119211)

Lane 3: Recombinant Human GAPDH protein (**ab77109**) at 1 ug + Instant Blue stain (ab119211)

Lane 4: Recombinant Human GAPDH protein (**ab77109**) at 0.5 ug + Instant Blue stain (ab119211)

Lane 5: Recombinant Human GAPDH protein (**ab77109**) at 0.3 ug + Instant Blue stain (ab119211)

Lane 6: Recombinant Human GAPDH protein (**ab77109**) at 0.2 ug + Instant Blue stain (ab119211)

Lane 7: Recombinant Human GAPDH protein (**ab77109**) at 0.1 ug + Instant Blue stain (ab119211)

Predicted band size: 36 kDa

Observed band size: 36 kDa

SDS-PAGE image showing molecular weight marker (MW) and Recombinant Human GAPDH protein in lanes 1-7 stained with

InstantBlue. The gel was stained for 30 minutes and then rinsed once with distilled water to remove the stain.

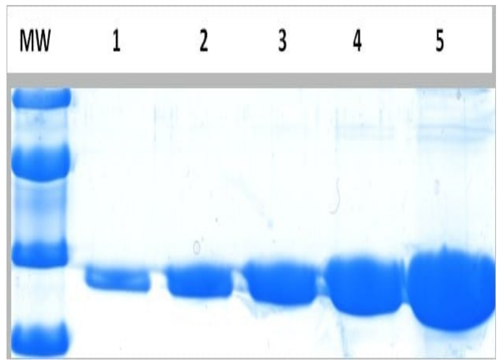

Western blot - InstantBlue® Coomassie Protein Stain (ISB1L) (ab119211)

This image is courtesy of an anonymous abreview.

SDS-PAGE image showing molecular weight marker (MW) and His-tag recombinant protein fractions purified in Ni-NTA resin in lanes 1-5 stained with InstantBlue. InstantBlue was easy to handle and use - just pour a small amount onto your gel and within 10 minutes you can see the protein bands! No methanol and glacial acetic acid - no smell!

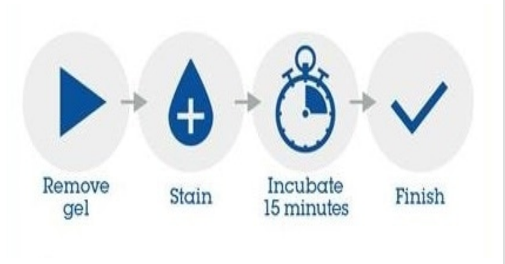

Western blot - InstantBlue® Coomassie Protein Stain (ISB1L) (ab119211)

Please note: All products are "FOR RESEARCH USE ONLY. NOT FOR USE IN DIAGNOSTIC PROCEDURES"

Our Abpromise to you: Quality guaranteed and expert technical support

- Replacement or refund for products not performing as stated on the datasheet
- Valid for 12 months from date of delivery
- Response to your inquiry within 24 hours

- We provide support in Chinese, English, French, German, Japanese and Spanish
- Extensive multi-media technical resources to help you
- We investigate all quality concerns to ensure our products perform to the highest standards

If the product does not perform as described on this datasheet, we will offer a refund or replacement. For full details of the Abpromise, please visit **https://www.abcam.co.jp/abpromise** or contact our technical team.

Terms and conditions

- Guarantee only valid for products bought direct from Abcam or one of our authorized distributors

TECHNICAL MANUAL

ONE-Glo™ EX Luciferase Assay System

Instructions for Use of Products
E8110, E8120, E8130 and E8150

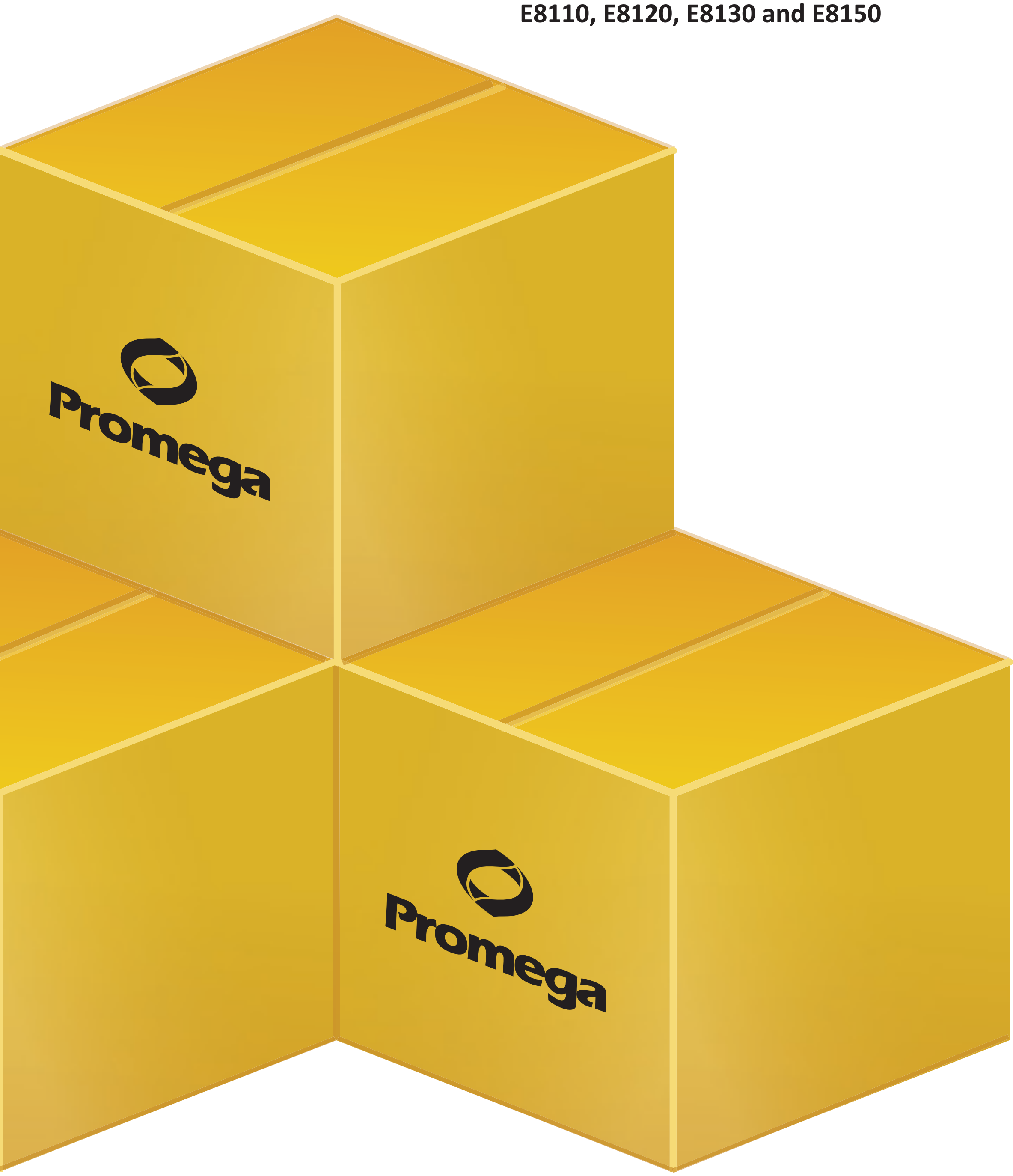

3/15
TM432

ONE-Glo™ EX Luciferase Assay System

All technical literature is available at: www.promega.com/protocols/
Visit the web site to verify that you are using the most current version of this Technical Manual.
E-mail Promega Technical Services if you have questions on use of this system: techserv@promega.com

1. Description 1
2. Product Components and Storage Conditions 2
3. Performing the ONE-Glo™ EX Luciferase Assay 3
   3.A. General Considerations 3
   3.B. Reagent Preparation 3
   3.C. Assay Procedure 4
4. Firefly Luciferase Vectors for Use with the ONE-Glo™ EX Luciferase Assay System 4
5. Appendix 4
   5.A. Overview of the ONE-Glo™ EX Luciferase Assay System 4
   5.B. Effects of Typical Reaction Conditions 6
   5.C. Reference 11
6. Related Products 12

1. Description

High- or ultrahigh-throughput quantitation of luciferase expression in mammalian cells is commonly performed by measuring luminescence from 96-, 384- or 1,536-well plates. The ONE-Glo™ EX Luciferase Assay System[a,b] provides both the high sensitivity and long-lived luminescence required to batch process multiple plates in these assay formats. The ONE-Glo™ EX Assay retains many of the beneficial aspects of the ONE-Glo™ Assay, using 5´-fluoroluciferin as substrate with an add-mix-read, or homogeneous, protocol. Extending the properties of ONE-Glo™ Reagent, ONE-Glo™ EX Reagent employs a new assay chemistry to increase both the stability of the luminescence signal and greatly increase the stability of the reconstituted reagent. The approximately 2 hour signal half-life provides greater flexibility in assay design. A reconstituted reagent that can be stored at room temperature for longer periods means less variability in reagent performance during long experiments or screens and less sample waste. ONE-Glo™ EX Reagent is the firefly luciferase detection reagent used in the Nano-Glo® Dual-Luciferase® Reporter (NanoDLR™) Assay System, allowing the same reagent to be used for single- or dual-luciferase assays.

2. Product Components and Storage Conditions

PRODUCT	SIZE	CAT.#
**ONE-Glo™ EX Luciferase Assay System**	**10ml**	**E8110**

Each system contains sufficient components to prepare 10ml of reagent. Includes:

- 10ml ONE-Glo™ EX Luciferase Assay Buffer
- 1 vial ONE-Glo™ EX Luciferase Assay Substrate (lyophilized)

PRODUCT	SIZE	CAT.#
**ONE-Glo™ EX Luciferase Assay System**	**100ml**	**E8120**

Each system contains sufficient components to prepare 100ml of reagent. Includes:

- 100ml ONE-Glo™ EX Luciferase Assay Buffer
- 1 vial ONE-Glo™ EX Luciferase Assay Substrate (lyophilized)

PRODUCT	SIZE	CAT.#
**ONE-Glo™ EX Luciferase Assay System**	**10 × 10ml**	**E8130**

Each system contains sufficient components to prepare 100ml of reagent. Includes:

- 10 × 10ml ONE-Glo™ EX Luciferase Assay Buffer
- 10 vials ONE-Glo™ EX Luciferase Assay Substrate (lyophilized)

PRODUCT	SIZE	CAT.#
**ONE-Glo™ EX Luciferase Assay System**	**10 × 100ml**	**E8150**

Each system contains sufficient components to prepare 1,000ml of reagent. Includes:

- 10 × 100ml ONE-Glo™ EX Luciferase Assay Buffer
- 10 vials ONE-Glo™ EX Luciferase Assay Substrate (lyophilized)

Storage Conditions: Store the ONE-Glo™ EX Luciferase Assay components at –10°C to –30°C. The ONE-Glo™ EX Luciferase Assay Buffer may be stored at 4°C for 1 year or at room temperature for 6 months.

Reconstituted ONE-Glo™ EX Reagent can be stored at 4°C or –20°C for later use, protected from light. Warm or thaw reagent at temperatures below 25°C to ensure optimal performance (e.g., place the reagent in a water bath at room temperature). Mix by inversion after thawing. ONE-Glo™ EX Reagent will lose 10% activity in approximately 18 hours and 50% activity in approximately 6 days at 22°C. At 4°C, the ONE-Glo™ EX Reagent will lose 10% activity in approximately 3.5 days and 50% activity in approximately 1 month.

3. Performing the ONE-Glo™ EX Luciferase Assay

3.A. General Considerations

The ONE-Glo™ EX Luciferase Assay System has been designed to be used with many media types and has been verified for use with the following culture media containing 0–10% serum: DMEM, RPMI 1640, McCoy's 5A and F-12. While the reagent should give a signal half-life of approximately 2 hours at 22°C in many media types, different combinations of media and serum may affect the luminescence or signal decay rate (see Section 5.B). The luminescence can also be affected by the presence of phenol red, organic solvents and changes in temperature (Section 5.B).

Because luminescent signals are affected by assay conditions, raw results should be compared only between samples measured at the same time and using the same medium and serum combination. For analysis of multiple plates, the greatest accuracy can be obtained by incorporating a common control sample in each plate. This corrects the small variations in luminescence that can occur over time or due to other variables such as temperature.

To achieve linear assay performance at low light levels, subtract the background luminescence from all readings. Background luminescence is a characteristic of luminometer performance as the ONE-Glo™ EX Reagent and mammalian cells lacking the luciferase gene produce no background. Some instruments also require verification of linear response at high light levels (consult the instrument manual).

3.B. Reagent Preparation

Transfer the contents of one bottle of ONE-Glo™ EX Buffer to one bottle of ONE-Glo™ EX Substrate. Replace the stopper and mix by inversion until the substrate is thoroughly dissolved. This should take less than 10 seconds.

Notes:

1. Due to the temperature dependency of luciferase activity, the temperature of the samples and the reagent should be kept constant while measuring luminescence. Ensure that the reagent is equilibrated to room temperature before use (e.g., placing the buffer at room temperature at least a day before experiments). Equilibrate cultured cells to room temperature before adding the reagent.
2. If the reconstituted ONE-Glo™ EX Reagent is stored at 4°C or frozen, warm or thaw the reagent at temperatures below 25°C to ensure optimal performance (e.g., place the reagent in a water bath at room temperature). Mix by inversion after thawing.
3. Once reconstituted, the ONE-Glo™ EX Reagent will lose 10% activity in approximately 18 hours and 50% activity in approximately 6 days at 22°C. Unused reconstituted reagent can be stored at 4°C or –20°C for later use, protected from light. At 4°C, the ONE-Glo™ EX Reagent will lose 10% activity in approximately 3.5 days and 50% activity in approximately 1 month.

3.C. Assay Procedure

1. Remove plates from the incubator and equilibrate to room temperature. Use an opaque white tissue-culture plate to minimize cross-talk between wells and absorption of emitted light.

   ! Ensure that the plates are compatible with the type of luminometer being used.

2. Add a volume of ONE-Glo™ EX Reagent that is equal to the volume of culture medium in each well, and incubate samples for at least 3 minutes to lyse cells and equilibrate samples. For optimal results, mix samples by placing the plate on an orbital shaker at 300–600rpm for 1–3 minutes. For 96-well plates, typically 80–100µl volumes are used. For 384-well plates, 20–30µl volumes are typically used.

3. Measure luminescence in a luminometer or instrument capable of measuring luminescence.

   Notes:

   1. Consult the instrument manual for instructions. We recommend an integration time of 0.5–1 second for 96-well plates when using GloMax® instruments.
   2. For optimal results, measure luminescence within 2 hours of adding ONE-Glo™ EX Reagent. The luminescence intensity has a signal half-life of approximately 2 hours.

4. Firefly Luciferase Vectors for Use with the ONE-Glo™ EX Luciferase Assay System

To select a firefly luciferase reporter vector suitable for use with the ONE-Glo™ EX Luciferase Assay System, visit: **www.promega.com/luciferase-vectors**

5. Appendix

5.A. Overview of the ONE-Glo™ EX Luciferase Assay System

Reporter genes are routinely used to study a wide range of physiological events. Examples include the study of regulated gene expression and signal transduction, where reporter protein expression is used as a surrogate to monitor changes in gene transcription. Luciferase is a popular choice as a reporter for these applications because functional enzyme is created immediately upon translation, and the assay is rapid and easy to perform. Furthermore, the sensitivity and linearity of luciferase detection are unmatched when compared to alternative reporter proteins. For these reasons, luciferase is widely used in the biotechnology and pharmaceutical industries, including the automated platforms for high-throughput screening used in drug discovery (1).

Firefly luciferase is a 61kDa monomer that catalyzes the mono-oxygenation of beetle luciferin. The enzyme uses ATP and molecular oxygen as cosubstrates. The ONE-Glo™ EX Reagent uses a new assay chemistry that extends the properties of ONE-Glo™ Reagent to generate a bright luminescent signal with improved signal and reagent stability. The use of the luciferin analog, 5´-fluoroluciferin (Figure 1), in the ONE-Glo™ and ONE-Glo™ EX Assay Systems allows the assays to be performed at a lower pH. The new ONE-Glo™ EX assay chemistry provides several advantages over similar luciferase reagents including: 1) increased reconstituted reagent stability; 2) improved ONE-Glo™ EX Reagent signal half-life, 1.5-fold longer than that of ONE-Glo™ Reagent; 3) reduced quenching of luminescence by phenol red in cell culture medium; 4) eliminated odor-causing thiol compounds, such as DTT, in the reagent.

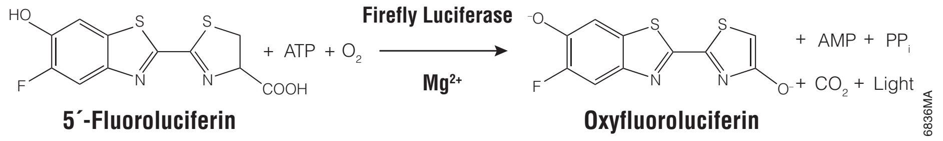

Figure 1. The bioluminescent reaction catalyzed by firefly luciferase in the ONE-Glo™ EX Assay.

To facilitate batch processing of plates and decrease variability within an experiment, the ONE-Glo™ EX reagent generates a bright, stable luminescent signal that decays at a steady rate with a half-life of approximately two hours in many media types (Figures 2 and 4). Compared to ONE-Glo™ Reagent, ONE-Glo™ EX Reagent produces a slightly dimmer signal (approximately 2/3 that of ONE-Glo™ Reagent), but with a compensating increase in the signal half-life (~115 versus ~75 minutes on average).

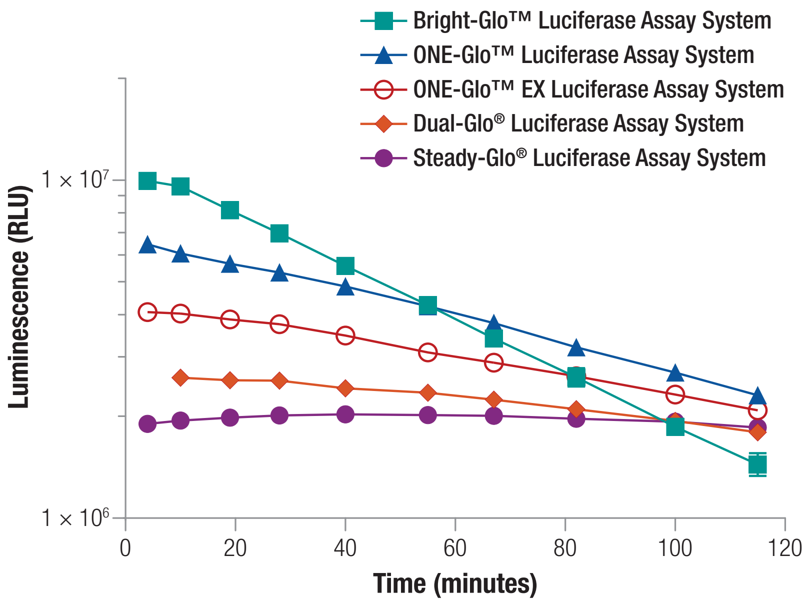

Figure 2. ONE-Glo™ EX Reagent provides bright and stable luminescence that can be measured for hours. One hundred microliters of purified firefly luciferase (13.8ng/ml in DMEM with 0.1% Prionex® as carrier) was combined in a 96-well plate with 100µl of Bright-Glo™, ONE-Glo™, ONE-Glo™ EX, Dual-Glo® Luciferase or Steady-Glo® Reagents. Luminescence was measured periodically over 2 hours, n = 8.

ONE-Glo™ EX Reagent was designed to provide extended stability once reconstituted compared to similar reagents, enabling more constant luminescence over long experiments. This reagent stability makes it more convenient to use, store and reuse than other luciferase reagents, reducing waste. Once reconstituted, ONE-Glo™ EX Reagent will lose 10% of its activity at 22°C after about 18 hours and lose 50% of its activity after about 6 days (Figure 3). Unused reagent can be stored at –20°C, but avoid multiple freeze-thaw cycles. The reagent can also be stored at 4°C, at which temperature it will lose 10% activity after about 3.5 days and 50% activity after about a month. After even prolonged incubation at room temperature (>3 weeks), the reagent may still retain about 25% of its original activity.

5.A. Overview of the ONE-Glo™ EX Luciferase Assay System (continued)

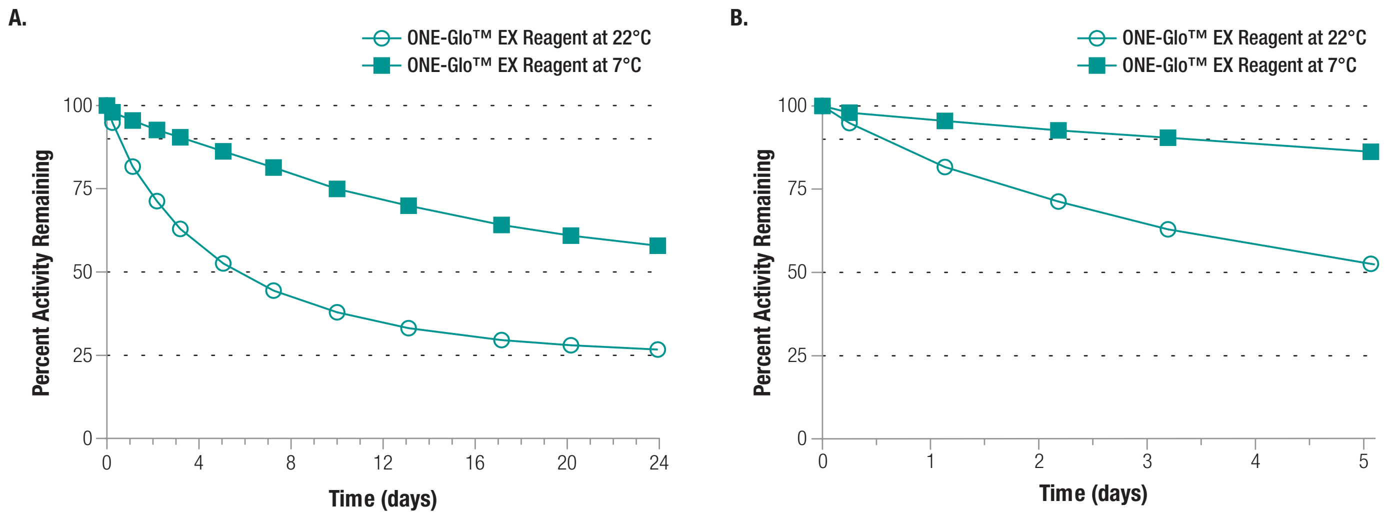

Figure 3. ONE-Glo™ EX Reagent displays extended stability once reconstituted. Reconstituted reagent was stored at 22°C or 7°C and frozen at –70°C at defined times. Upon thawing and equilibrating to room temperature, the ONE-Glo™ EX Reagent samples were combined with an equal volume of 13.8ng/ml purified firefly luciferase in DMEM supplemented with 10% fetal bovine serum (FBS). The relative functionality was calculated as the luminescence signal intensity for each sample, measured 3 minutes after enzyme addition, relative to the signal intensity of the sample that was placed at –70°C with no incubation at 22°C. The activity data is shown over 24 days (**Panel A**) and over just the first 5 days (**Panel B**); n=4.

5.B. Effects of Typical Reaction Conditions

Culture Medium

Like other add-mix-measure luciferase assays, half of the reaction volume for ONE-Glo™ EX reactions is mammalian tissue culture medium. The ONE-Glo™ EX Reagent is designed to work well with a variety of common media. However, differences between media can affect the intensity and duration of the luminescent signal (Figure 4). For instance, the phenol red in some media may decrease signal intensity. Differences in media or between different manufacturers or lots of the same medium make it important to incorporate controls in each batch of plates.

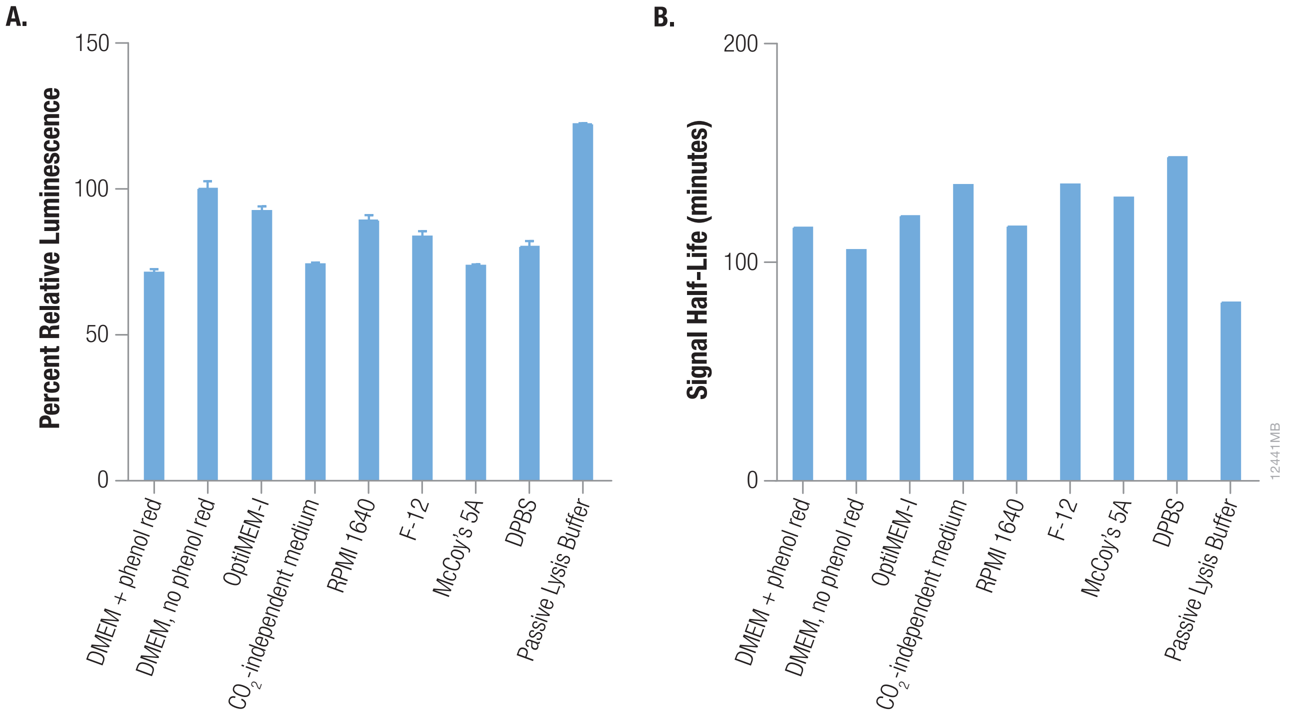

Figure 4. Relative luminescence intensity and signal stability of firefly luciferase in common media types. HEK293 cells were transiently transfected with firefly luciferase expression constructs. Cells were harvested, split equally among tubes, centrifuged and resuspended to 3.4×10^5 cells/ml in various media types supplemented with 10% fetal bovine serum (FBS) with the exception of Passive Lysis Buffer, which contained no serum and was allowed to incubate with the cells for 15 minutes. Eighty microliters of cells were added to the wells of a 96-well plate before dispensing 80µl of ONE-Glo™ EX Reagent. Luminescence was measured periodically over 2.5 hours. **Panel A.** The firefly luminescence at 3 minutes is shown relative to that measured from DMEM without phenol red; n = 4. **Panel B.** The signal stability in different media is expressed as the half-life of the signal decay over the 2.5 hours.

Promega

5.B. Effects of Typical Reaction Conditions (continued)

Serum

The ONE-Glo™ EX Reagent has been designed for use with 0–10% serum, and the luminescent signals generated are minimally affected by the presence of serum (Figure 5).

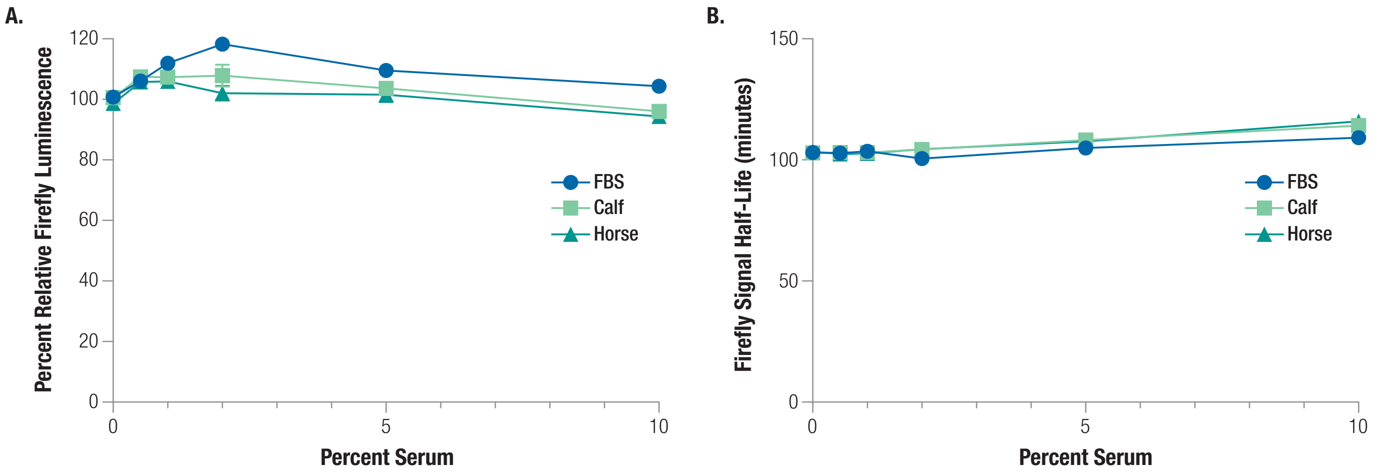

Figure 5. Effects of serum on luminescence intensity and signal stability. Purified firefly luciferase (6.88ng/ml) was diluted into DMEM plus 0.1% Prionex® with varying concentrations of fetal bovine serum (FBS), calf serum or horse serum. Enzyme solutions (80µl per well) were added to 96-well plates, then 80µl of ONE-Glo™ EX Reagent was dispensed. Luminescence was measured periodically over 2 hours to calculate the signal stability (half-life); n = 4. **Panel A.** Firefly luminescence relative to no serum. **Panel B.** Luminescent signal half-life.

Organic Solvents

Organic solvents may be present in reporter gene assays because they are used to solubilize screening compounds. DMSO, ethanol and methanol in concentrations up to 3% have little effect on luciferase luminescence or signal kinetics (Figure 6).

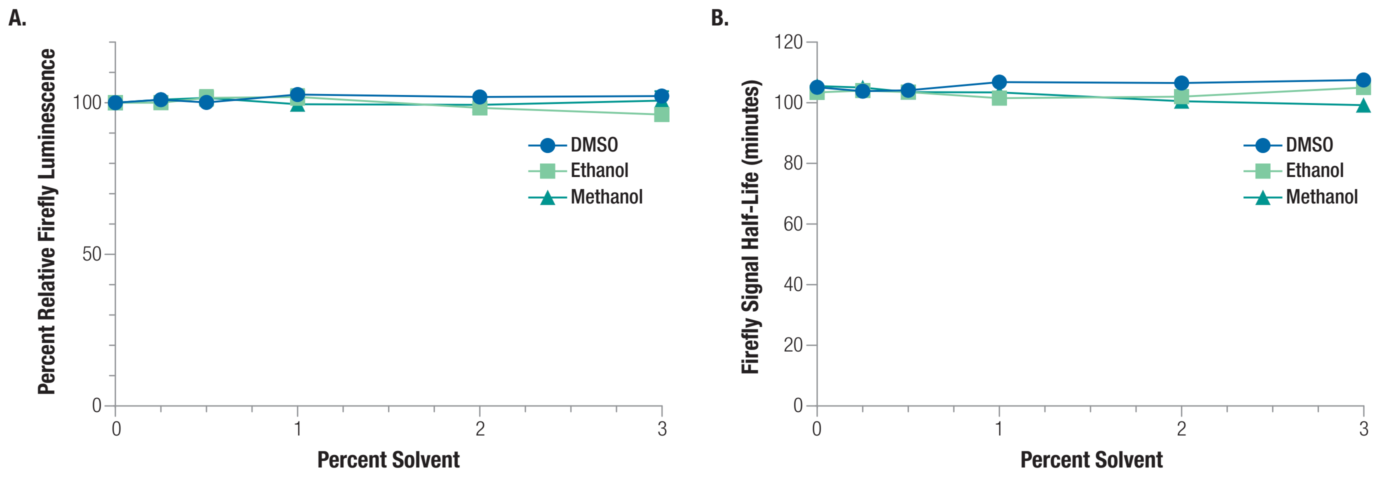

Figure 6. The effect of organic solvents on luminescence intensity and signal stability. Purified firefly luciferase (6.88ng/ml) was diluted into DMEM plus 0.1% Prionex® with varying concentrations of dimethyl sulfoxide (DMSO), ethanol or methanol. Enzyme solutions (80µl per well) were added to 96-well plates, and then 80µl of ONE-Glo™ EX Reagent was dispensed. Luminescence was measured periodically over 2 hours to calculate the signal stability (half-life); n = 4. **Panel A.** Firefly luminescence relative to no solvent. **Panel B.** The signal half-life.

5.B. Effects of Typical Reaction Conditions (continued)

Phenol Red

Phenol red is a pH indicator commonly used in cell culture media. Many commercial medium formulations contain 5–15mg/l phenol red, causing the characteristic red color. Because phenol red can absorb light, it may reduce assay sensitivity. However, the lower pH of the ONE-Glo™ EX Reagent makes it less sensitive to phenol red compared to other luciferase reagents (Figure 7). For most applications, the presence of phenol red will not significantly affect the the ONE-Glo™ EX Assay. However, to maximize the luminescent signal, use as little phenol red as possible in culture medium.

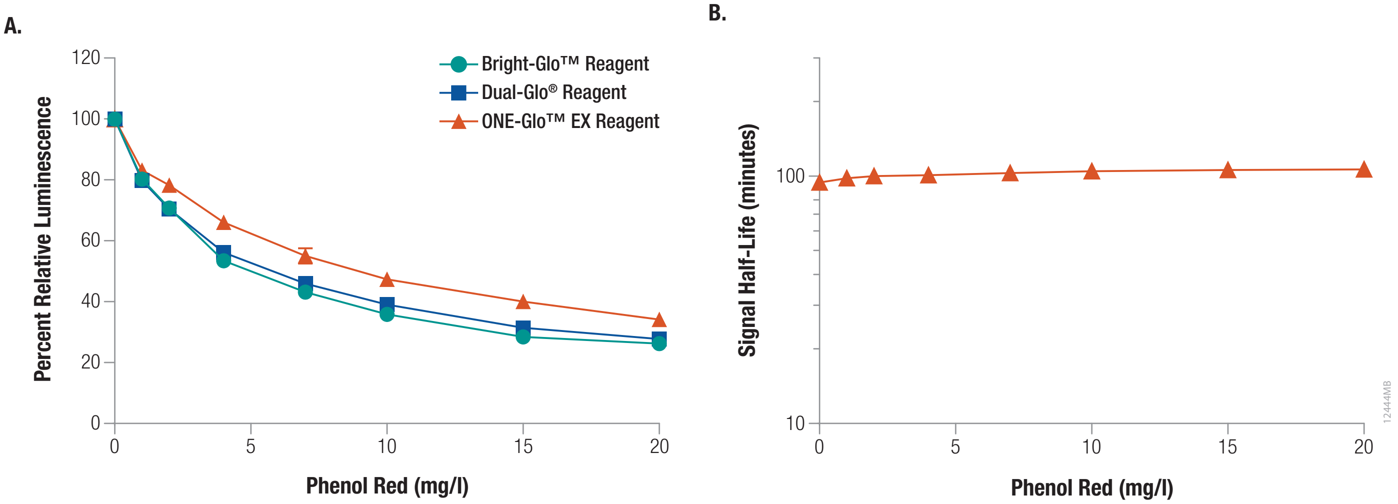

Figure 7. The effect of phenol red on luminescence intensity and stability. Purified firefly luciferase (6.88ng/ml) was diluted into DMEM plus 0.1% Prionex® with varying concentrations of phenol red. Enzyme solutions (80µl per well) were added to 96-well plates, then 80µl of Bright-Glo®, Dual-Glo® Luciferase or ONE-Glo™ EX Reagent was dispensed. Luminescence was measured periodically over 2 hours to calculate the signal stability (half-life); n = 3. **Panel A.** Firefly luminescence relative to no added phenol red. **Panel B.** Signal half-life for ONE-Glo™ EX Reagent.

Temperature

Because the activity of firefly luciferase is temperature sensitive, maintaining a consistent temperature is an important factor in experimental precision. Higher temperature causes higher signal intensity but lower signal stability (Figure 8). Precision can be most easily achieved by performing all experiments at room temperature. The assay reagents should be at room temperature before measuring luminescence, and the culture plate should be equilibrated to room temperature before adding reagents.

The ONE-Glo™ EX Buffer can be stored at room temperature prior to the experiment to eliminate the need for temperature equilibration before use. The heat capacity of the substrate is low; therefore, reconstitution of the substrate with room temperature buffer will produce reagents ready for use. If equilibration is needed to bring reagents to room temperature, incubate reagents in a water bath at room temperature (the water bath should not be set higher than 25°C).

Reaction temperatures can be affected by chilled reagent, culture plates that are too warm, excess heat within luminometers and other factors. If cold reagent is used, luminescence will slowly increase during the experiment as the reagent warms. Some luminometers run at a higher temperature than the ambient environment. To prevent signal gradients across a plate due to uneven warming of a plate during measurement, we recommed equilibrating plates and reagents to the internal temperature of the luminometer (e.g., in a water bath set to the higher-than-ambient temperature of the instrument).

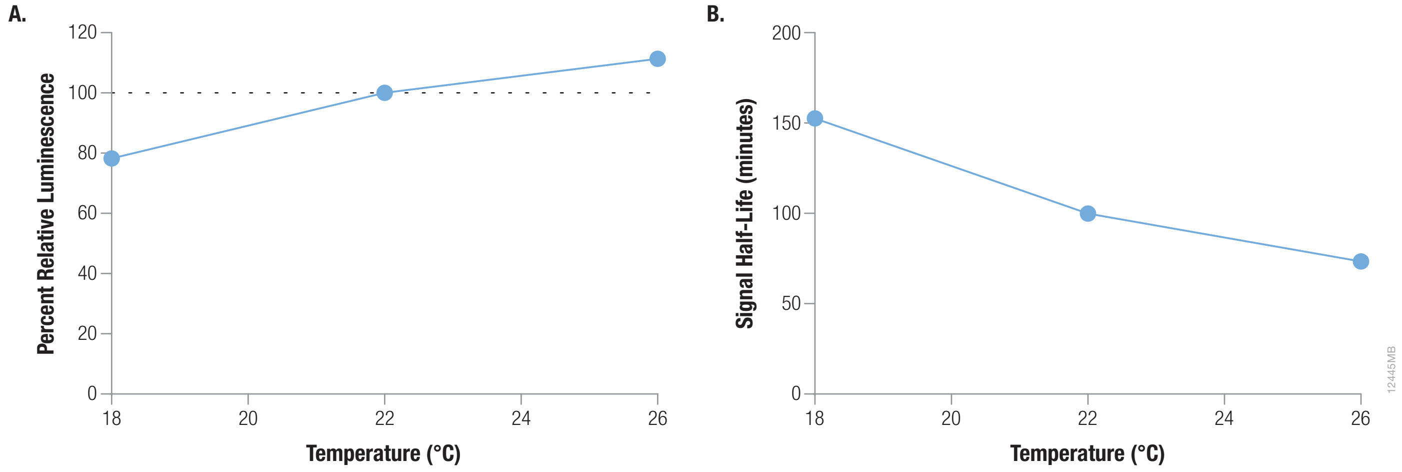

Figure 8. The effect of temperature on luciferase luminescence. Purified firefly luciferase (13.8ng/ml) was diluted into DMEM plus 0.1% Prionex®. Luciferase solution (200µl) was added to 200µl of ONE-Glo™ EX Reagent in a luminometer tube. Luminescence was measured periodically over 2 hours using a Turner Biosystems 20/20^n luminometer. The luciferase solutions and reagents were equilibrated at 18, 22 and 26°C prior to mixing and incubated at those same temperatures between measurements. **Panel A.** Luminescence after 3 minutes relative to the value at 22°C. **Panel B.** Signal half-life.

5.C. Reference

1. Fan, F. and Wood, K.V. (2007) Bioluminescent assays for high-throughput screening. *Assay Drug Dev. Technol.* **5**, 127–36.

6. Related Products

To find a firefly luciferase reporter vector for your experiment, visit: **www.promega.com/luciferase-vectors**

Luciferase Assay Systems

Product	Size	Cat.#
ONE-Glo™ Luciferase Assay System*	100ml	E6120
Steady-Glo® Luciferase Assay System*	100ml	E2520
Bright-Glo™ Luciferase Assay System*	100ml	E2620
Dual-Glo® Luciferase Assay System*	100ml	E2940
Dual-Luciferase® Reporter Assay System*	100 assays	E1910
Luciferase Assay System*	100 assays	E1500
Luciferase Assay Reagent	1,000 assays	E1483
Renilla-Glo® Luciferase Assay System*	100ml	E2720
Nano-Glo® Dual-Luciferase® Reporter (NanoDLR™) Assay System*	100ml	N1620
QuantiLum® Recombinant Luciferase*	1mg	E1701

*Additional Sizes Available.

Transfection Reagent

Product	Size	Cat.#
FuGENE® HD Transfection Reagent	1ml	E2311
	5 × 1ml	E2312
FuGENE® 6 Transfection Reagent	1ml	E2691
	0.5ml	E2693
	5 × 1ml	E2692
ViaFect™ Transfection Reagent	0.75ml	E4981
	2 × 0.75ml	E4982
Transfection Carrier DNA	5 × 20µg	E4881

Luminometers

Product	Cat.#
GloMax® Discover System	GM3000
GloMax®-Multi+ Detection System with Instinct™ Software Base Instrument with Shaking*	E8032
GloMax®-Multi+ Detection System with Instinct™ Software Base Instrument with Heating and Shaking*	E9032
GloMax®-Multi Base Instrument*	E7031
GloMax® 96 Microplate Luminometer	E6501
GloMax® 20/20 Luminometer	E5311

*Base instrument must be purchased with luminescence modules (e.g., E8032 and E9032 with E8041 or E7031 with E7041).

[a]Patent Pending.

[b]Certain applications of this product may require licenses from others.

©2015 Promega Corporation. All Rights Reserved.

Dual-Glo, Dual-Luciferase, GloMax, Nano-Glo, QuantiLum, *Renilla*-Glo and Steady-Glo are registered trademarks of Promega Corporation. Bright-Glo, NanoDLR, ONE-Glo and ViaFect are trademarks of Promega Corporation.

FuGENE is a registered trademark of Fugent, L.L.C., USA. Prionex is a registered trademark of Pentapharm Ltd.

Products may be covered by pending or issued patents or may have certain limitations. Please visit our Web site for more information.

All prices and specifications are subject to change without prior notice.

Product claims are subject to change. Please contact Promega Technical Services or access the Promega online catalog for the most up-to-date information on Promega products.

ThermoFisher SCIENTIFIC **Life Technologies** **CERTIFICATE OF ANALYSIS**

Catalog Number L34966

Product Name LIVE/DEAD™ Fixable Aqua Dead Cell Stain Kit *for 405 nm excitation**400 assays*

Lot Number 2971004

	LOT DATA	SPECIFICATION
**ASSAY**<br>Test	meets specification	This kit has been tested and found to conform to Life Technologies' quality standard.

Zach Luedtke, Quality Assurance Manager
31-Jul-2024

Life Technologies Corporation certifies on the date above that this is an accurate record of the analysis of the subject lot, and that the data conform to the specifications in effect for this product at the time of analysis.
Products are under warranty for one year from the date of shipment unless otherwise stated on this document or the product literature.

Thermo Fisher Scientific
29851 Willow Creek Road
Eugene, OR 97402-9132
Phone +1-541-465-8300 Fax +1-541-335-0504

invitrogen USER GUIDE

LIVE/DEAD™ Fixable Dead Cell Stain Kits

Pub. No. MAN0002416 (MP34955) **Rev.** A.0

Table 1. Contents and storage

Material	Amount	Storage	Stability
Individual Kits: Blue, violet, aqua, yellow-, green, red, far red, or near-IR fluorescent reactive dye (Component A)	2 vials, each (80 assays);<br>5 vials, each (200 assays);<br>10 vials, each (400 assays)	• ≤–20°C<br>• Desiccate<br>• Protect from light	When stored as directed, kit components are stable for at least 6 months.
Sampler Kit: Blue, violet, aqua, yellow, green, red, far red, and near-IR fluorescent reactive dye (Components A–H)	1 vial of each dye (8 total)		
Dimethylsulfoxide (DMSO), anhydrous (Component B in Individual Kits, Component I in Sampler Kit)	500 µL		
**Number of assays:** Each vial of the reactive dye provides sufficient material for approximately 40 flow cytometry assays, for a total of 80, 200, or 400 assays depending on the kit configuration (see Table 2). The Sampler Kit contains one vial of each fluorescent reactive dye with 40 assays per vial of the reactive dye, for a total of 320 assays per kit.			
**Approximate fluorescence excitation/emission maxima:** See Table 2.			

Introduction

The LIVE/DEAD™ Fixable Dead Cell Stain Kits use a novel method to evaluate the viability of mammalian cells by flow cytometry. These assays are based on the reaction of a fluorescent reactive dye with cellular amines. The reactive dye can permeate the compromised membranes of necrotic cells and react with free amines both in the interior and on the cell surface, resulting in intense fluorescent staining. In contrast, only the cell-surface amines of viable cells are available to react with the dye, resulting in relatively dim staining (Figure 1, page 3). The difference in intensity between the live and dead cell populations is typically greater than 50-fold (Figure 2, page 3). The discrimination is completely preserved following fixation of the sample by formaldehyde, under conditions that inactivate pathogens. Moreover, these single-color assays use only one channel of a flow cytometer, leaving the other channels available for multicolor experiments. The assays can also be used to detect dead cells by microscopy; however, the difference in fluorescence intensity of the live and dead cells can be appreciable, making it relatively difficult to simultaneously photograph the two populations.

The single-color LIVE/DEAD™ Fixable Dead Cell Stain Kits are identical except for the fluorescent color of the included dye—blue, violet, aqua, yellow, green, red, far red, or near-IR (infra red).

- Cells labeled by green fluorescent or red fluorescent reactive dye are excited by the 488 nm line of an argon-ion laser; green fluorescence is typically detected in the green channel of the flow cytometer (530/30 nm) and red fluorescence is detected in the red channel (630/30 nm). The blue fluorescent reactive dye requires UV (350–360 nm) excitation with fluorescence emission read at ~450 nm.
- The violet fluorescent reactive dye requires violet (~405 nm) excitation with fluorescence emission read at ~440 nm.
- The aqua fluorescent reactive dye is efficiently excited with ~405 nm light (or UV light) and has fluorescence emission monitored at ~525 nm.
- The yellow fluorescent reactive dye requires violet (~405 nm) excitation with fluorescence emission read at ~575 nm (appropriate channels for the violet-excitable reactive dyes may vary depending on the instrument).
- The far red and near-IR fluorescent reactive dyes are excited at 633/635 nm with fluorescence emission monitored at 665 nm and 775 nm, respectively.
- The LIVE/DEAD™ Fixable Dead Cell Stain Sampler Kit (Cat. no. L34960) contains one vial of each of the eight different fluorescent reactive dyes.

We also offer a LIVE/DEAD™ Reduced Biohazard Cell Viability Kit (Cat. no. L7013) for determining cell viability. It is based on a different staining principle using two dyes of different colors, and is described separately. For additional information, visit **www.thermofisher.com**.

Table 2. Approximate fluorescence excitation and emission maxima and kit sizes for the LIVE/DEAD™ Fixable Dead Cell Stain single-color dyes.

Reactive dye	Number of assays (Cat. no.)	Excitation source	Ex*	Em*
Blue fluorescent reactive dye	80 (L34961)<br>200 (L23105)<br>400 (L34962)	UV	350 nm	450 nm
Violet fluorescent reactive dye	80 (L34963)<br>200 (L34955)<br>400(L34964)	405 nm	416 nm	451 nm
Aqua fluorescent reactive dye	80 (L34965)<br>200 (L34957)<br>400 (L34966)	405 nm	367 nm	526 nm
Yellow fluorescent reactive dye	80 (L34967)<br>200 (L34959)<br>400 (L34968)	405 nm	400 nm	575 nm
Green fluorescent reactive dye	80 (L34969)<br>200 (L23101)<br>400 (L34970)	488 nm	495 nm	520 nm
Red fluorescent reactive dye	80 (L34971)<br>200 (L23102)<br>400 (L34972)	488 nm	595 nm	615 nm
Far red fluorescent reactive dye	80 (L34973)<br>200 (L10120)<br>400 (L34974)	633/635 nm	650 nm	665 nm
Near-IR fluorescent reactive dye	80 (L34975)<br>200 (L10119)<br>400 (L34976)	633/635 nm	750 nm	775 nm
*Approximate fluorescence excitation (Ex) and emission (Em) maxima, in nm.				

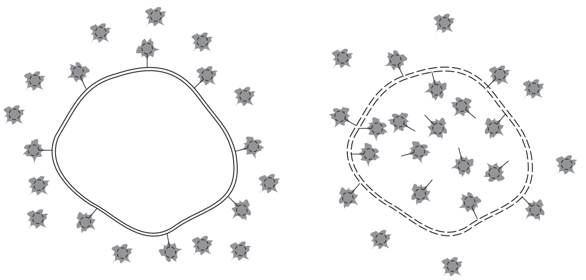

Figure 1. Principle of the LIVE/DEAD™ Fixable Dead Cell Stain Kits. Live cells (left) react with the kit's fluorescent reactive dye only on their surface to yield weakly fluorescent cells. Cells with compromised membranes (right) react with the dye throughout their volume, yielding brightly stained cells. In both cases, the excess reactive dye is subsequently washed away.

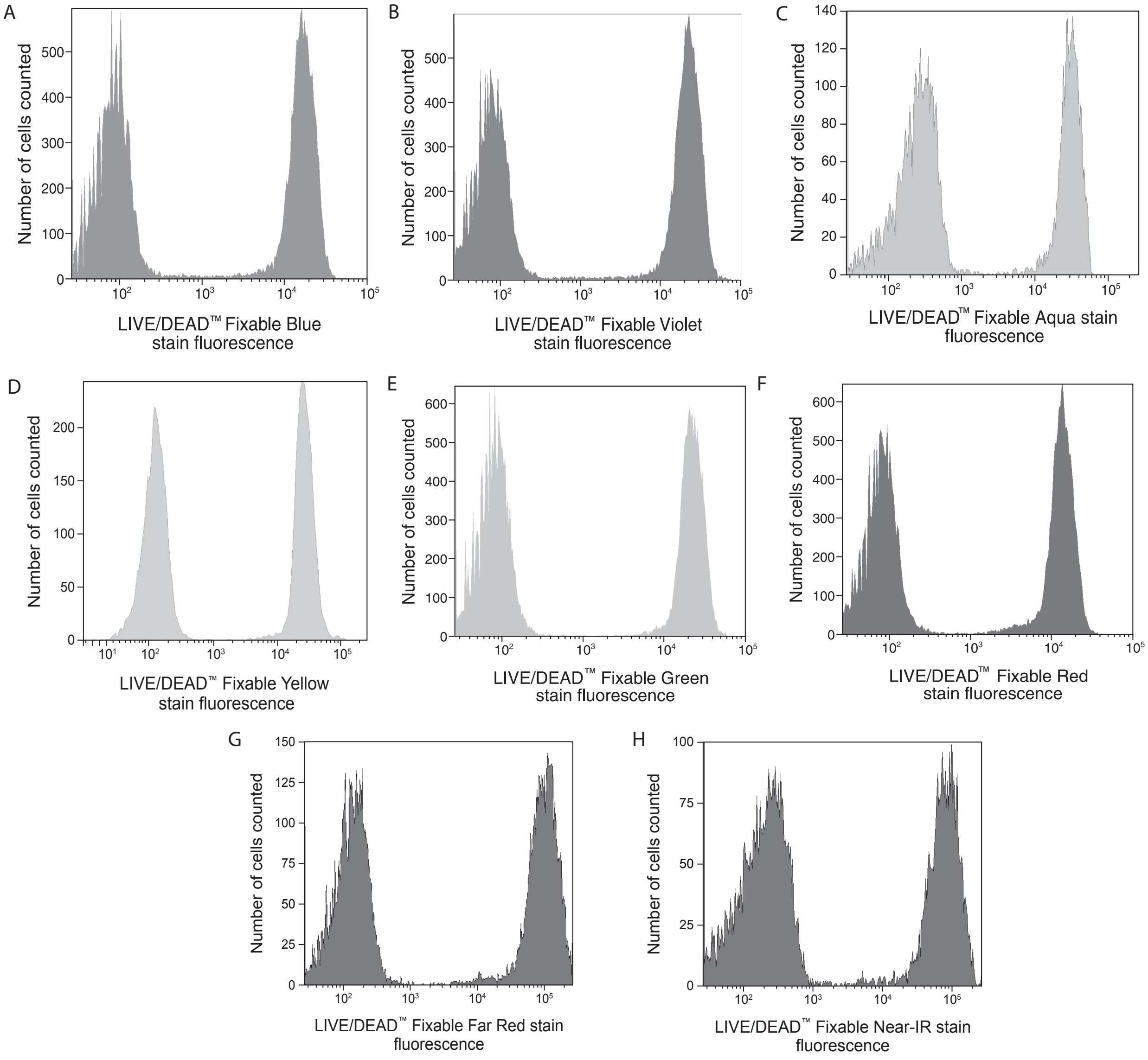

Figure 2. Live and dead cells distinguished by flow cytometry. Each of the LIVE/DEAD™ Fixable Dead Cell Stain Kits was used to differentially stain a mixture of live (left peak) and heat-treated Jurkat cells (right peak) according to the protocol provided in this document. **(A)** LIVE/DEAD™ Fixable Blue Stain Kit with UV excitation and ~440 nm emission; **(B)** LIVE/DEAD™ Fixable Violet Stain Kit with violet excitation and ~440 nm emission; **(C)** LIVE/DEAD™ Fixable Aqua Dead Cell Stain Kit using 405 nm excitation and ~525 nm emission; **(D)** LIVE/DEAD™ Fixable Yellow Stain Kit with 405 nm excitation and ~575 nm emission; **(E)** LIVE/DEAD™ Fixable Green Stain Kit with 488 nm excitation and ~525 nm emission; **(F)** LIVE/DEAD™ Fixable Red Stain Kit with 488 nm excitation and ~585 nm emission; **(G)** LIVE/DEAD™ Fixable Far Red Stain Kit with 633 nm excitation and ~660 nm emission; and **(H)** LIVE/DEAD™ Fixable Near-IR Stain Kit with 633 nm excitation and ~780 nm emission. Following the staining reaction, the cells were fixed in 3.7% formaldehyde and analyzed by flow cytometry. The live cell population is easily distinguished from the killed population, and nearly identical results were obtained using unfixed cells (data not shown).

Before You Begin

Allow the reagents to warm to room temperature before opening the vials.

Materials required but not provided

- Phosphate buffered saline (PBS)
- PBS with 1% bovine serum albumin (BSA)
- Formaldehyde
- AbC™ Total Antibody Compensation Bead Kit (if calculating compensation in multicolor immunophenotyping experiments using mouse, rat, rabbit, or hamster antibodies)
- ArC™ Amine Reactive Compensation Bead Kit (if calculating compensation in multicolor experiments using a LIVE/DEAD™ Fixable Dead Cell Stain)

Caution DMSO is hazardous; avoid contact with skin and eyes and do not swallow. Handle reagents containing DMSO using equipment and practices appropriate for the hazards posed by such materials.

Experimental Protocols

The following protocol has been used successfully in our laboratories to differentially stain live and dead cells and then to fix the cells in formaldehyde for subsequent analysis by flow cytometry. Excellent results have been obtained with a variety of cell types, including: Jurkat, MDCK, COLO205, CHO-K1, K-562, HeLa, P3X, 3T3, B35, BPAC, CHO-M1, A549, MMM, MRC5, U205, U266, 293 MSR, and RAW tissue-culture cell lines and human peripheral blood lymphocytes. If another staining reaction is to be performed on the same sample, determine the optimal staining sequence for the two procedures and whether or not the additional staining reaction will tolerate fixation by formaldehyde.

Dye preparation For convenience, the reactive dye in each individual kit is supplied in five separate vials, while the sampler kit contains one vial of each reactive dye. Each vial provides sufficient material for staining at least 40 cell samples. However, once reconstituted, the DMSO solution of reactive dye is somewhat unstable, especially if exposed to moisture. Unused portions may be used for up to 2 weeks if stored at ≤–20°C, protected from light and moisture.

1.1 Bring one vial of the fluorescent reactive dye (Component A) and the vial of anhydrous DMSO (Component B) to room temperature before removing the caps.

1.2 Add 50 µL of DMSO to the vial of reactive dye. Mix well and visually confirm that all of the dye has dissolved.

1.3 Use the solution of reactive dye as soon as possible (see below), ideally within a few hours of reconstitution.

Cell staining

Buffers appropriate for cell staining include phosphate-buffered saline (PBS), Hanks' Balanced Salt Solution (HBSS), and Dulbecco's PBS (D-PBS), without extraneous proteins such as bovine serum albumin or serum. When using an amino-reactive dye, for example the violet fluorescent reactive dye, Tris buffers and solutions containing sodium azide or extraneous protein should not be used for cell resuspension and washing.

2.1 Centrifuge a sample of cells in suspension containing at least 1×10^6 cells. Discard the supernatant.

2.2 Wash the cells once with 1 mL of PBS.

2.3 Resuspend the cells in 1 mL of PBS.

2.4 Count the cells and adjust the density with PBS to 1×10^6 cells in a 1 mL volume.

2.5 Add 1 µL of the reconstituted fluorescent reactive dye (from step 1.3) to 1 mL of the cell suspension and mix well.

2.6 Incubate at room temperature or on ice for 30 minutes, **protected from light**.

Note: If fixation is not required, then you can skip the steps 2.7–2.10 below. Instead, wash the cells twice with 1 mL of PBS with 1% bovine serum albumin, and resupend in 1 mL of PBS with 1% bovine serum albumin.

2.7 Wash the cells once with 1 mL of PBS, and resuspend the cells in 900 µL of PBS.

2.8 Add 100 µL of 37% formaldehyde.

2.9 Incubate at room temperature for 15 minutes.

2.10 Wash once with 1 mL of PBS with 1% bovine serum albumin, and resuspend the cells in 1 mL of PBS with 1% bovine serum albumin.

2.11 Analyze the fixed cell suspension by flow cytometry using the appropriate excitation and detection channel (see Table 3, below).

Note: The appropriate excitation and detection channels may vary depending on instrument used.

Table 3. Appropriate excitation and detection channels to use with the amine-reactive LIVE/DEAD™ fixable dead cell stains

Reactive dye	Excitation	Detection*	Recommended filters
Blue fluorescent reactive dye	UV	~450 nm	450/50 nm or similar
Violet fluorescent reactive dye	405 nm	~450 nm	450/50 nm or similar
Aqua fluorescent reactive dye	405 nm	~525 nm	525/50 nm or similar
Yellow fluorescent reactive dye	405 nm	~575 nm	575/26 nm, 585/42 nm, or similar
Green fluorescent reactive dye	488 nm	~530 nm	530/30 nm or similar
Red fluorescent reactive dye	488 nm	~585 nm	585/42 nm, 610/20 nm, or similar
Far red fluorescent reactive dye	633/635 nm	~660 nm	
Near-IR fluorescent reactive dye	633/635 nm	~780 nm	

*For the approximate fluorescence excitation and emission maxima of the amine-reactive LIVE/DEAD™ dyes, see Table 2 (page 2) and Figure 2 (page 3).

Intracellular staining

This procedure is completely compatible with common fixation and permeabilization methods for performing intracellular staining for flow cytometry. The procedure for staining with a LIVE/DEAD™ Fixable Dead Cell Stain combined with immunophenotyping is as follows:

3.1 Centrifuge a sample of cells in suspension containing at least 1×10^6 cells. Discard the supernatant.

3.2 Wash the cells once with 1 mL of PBS.

3.3 Resuspend the cells in 1 mL of PBS.

3.4 Count the cells and adjust the density with PBS to 1×10^6 cells in a 1 mL volume.

3.5 Add 1 µL of the reconstituted fluorescent reactive dye (from step 1.3) to 1 mL of the cell suspension and mix well.

3.6 Incubate at room temperature or on ice for 30 minutes, **protected from light**.

3.7 Wash the cells with 1 mL of PBS and resuspend in 100 µl of PBS.

3.8 Stain as usual for surface markers, and incubate for desired time for antibody staining.

3.9 Wash the cells with PBS and resuspend in 900 µl of PBS

3.10 Add 100 µL of 37% formaldehyde.

3.11 Incubate at room temperature for 15 minutes.

3.12 Wash once with 1 mL of PBS with 1% bovine serum albumin, and resuspend the cells in 100 µL of PBS with 1% bovine serum albumin.

3.11 Add permeabilization reagent and stain as usual for intracellular markers. Incubate for the desired time for antibody staining.

3.12 Wash once with 1 mL of PBS with 1% bovine serum albumin, and resuspend the cells in 1 mL of PBS with 1% bovine serum albumin.

3.13 Analyze the fixed cell suspension by flow cytometry using the appropriate excitation and detection channel (from step 2.11)

Compensation Using ArC™ Amine Reactive Beads

The ArC™ Amine Reactive Compensation Bead Kit is designed to facilitate compensation when using any of the LIVE/DEAD™ fixable dead cell stains, providing a consistent, accurate and simple-to-use technique for the setting of flow cytometry compensation. The ArC™ Amine Reactive Compensation Bead Kit includes two types of specially modified polystyrene microspheres to allow easy compensation of the LIVE/DEAD™ fixable stains: the ArC™ reactive beads (Component A), which bind any of the amine-reactive dyes, and the ArC™ negative beads (Component B), which have no reactivity. After incubation with any amine-reactive dye, the two kit components will provide distinct positive and negative populations of beads that can be used to set compensation. We recommend the following protocol for using ArC™ Amine Reactive Compensation Bead Kit for compensation:

4.1. Gently vortex ArC™ Amine Reactive Compensation Bead Kit components for 30 seconds to completely resuspend before use.

4.2. Add 1 drop of ArC™ reactive beads (Component A) to a labeled sample tube.

4.3. Allow ArC™ reactive beads to sit in the tube for 5 minutes to warm to room temperature.

4.4. Prepare fluorescent amine-reactive dye according to instructions included in the LIVE/DEAD™ Fixable Dead Cell Kit. For optimal performance of the ArC™ reactive beads, use freshly prepared amine-reactive dye. Do not use previously frozen dye solution.

4.5. Add the amount of LIVE/DEAD™ fixable dead cell stain listed in Table 4, below, to the bead suspension and mix well. Make sure to deposit the amine-reactive dye directly to the bead suspension.

Table 4. Amount of amine-reactive LIVE/DEAD™ fixable dead cell stain for use with ArC™ reactive beads

Amine-reactive dye for use with ArC™ Reactive Beads	Amount
LIVE/DEAD™ Fixable Blue stain	3 µL
LIVE/DEAD™ Fixable Violet stain	1 µL
LIVE/DEAD™ Fixable Aqua stain	3 µL
LIVE/DEAD™ Fixable Yellow stain	3 µL
LIVE/DEAD™ Fixable Green stain	3 µL
LIVE/DEAD™ Fixable Red stain	1 µL
LIVE/DEAD™ Fixable Far Red stain	3 µL
LIVE/DEAD™ Fixable Near-IR stain	1 µL

4.6. Incubate for 30 minutes at room temperature, **protected from light**.

4.7. Add 3 mL of PBS or other buffer to sample tube. Centrifuge for 5 minutes at 300 × *g*.

4.8. Carefully remove all the supernatant from tube.

Note: If using the red fluorescent reactive dye, repeat step 4.7.

4.9. Resuspend bead pellet by adding 0.5 mL of buffer to sample tube

4.10. Add one drop of ArC™ negative beads (Component B) to sample tube. Mix thoroughly.

4.11. Vortex tubes before analyzing using flow cytometry.

4.12. Perform manual or automatic compensation according to the preferred procedure for the flow cytometer in use. Gate on the bead singlet population based on FSC and SSC characteristics.

Combining ArC™ and AbC™ Kits

The AbC™ Total Antibody Compensation Bead Kit (Cat. nos. A10497, A105213) provides a consistent, accurate, and simple-to-use technique for the setting of flow cytometry compensation when using fluorochrome-conjugated mouse antibodies. The kit contains two types of specially modified polystyrene microspheres: the AbC™ capture beads (Component A) that bind all isotypes of mouse, rat, rabbit, and hamster immunoglobulin, and the negative beads (Component B) that have no antibody binding capacity. After incubating with a fluorochrome-conjugated antibody, the two components provide distinct positive and negative populations of beads that you can use to set compensation. You can use the AbC™ Total Antibody Compensation Bead Kit and the ArC™ Amine Reactive Compensation Bead Kit together to calculate compensation in multicolor immunophenotyping experiments that incorporate a LIVE/DEAD™ fixable dye by following the protocol outlined below:

5.1. Gently vortex the ArC™ Amine Reactive Compensation Bead Kit and the AbC™ Total Antibody Compensation Bead Kit components for 30 seconds to completely resuspend before use.

5.2. Label a sample tube for the amine-reactive dye you are using and add 1 drop of ArC™ reactive beads (Component A in the ArC™ Amine Reactive Compensation Bead Kit) to the labeled sample tube. Allow ArC™ reactive beads to sit in the tube for 5 minutes to warm to room temperature.

5.3. Prepare fluorescent reactive dye according to kit instructions included in the LIVE/DEAD™ Fixable Dead Cell Stain Kit. For optimal performance of ArC™ reactive beads, always use freshly prepared amine-reactive dye. Do **not** use previously frozen dye solution.

5.4. Add the amount of LIVE/DEAD™ fixable dead cell stain listed in Table 4 (page 7) to the bead suspension and mix well. Make sure to deposit the amine-reactive dye directly to the bead suspension.

5.5. Label another sample tube for each fluorochrome-conjugated antibody you are using, and add 1 drop of AbC™ capture beads (Component A in the AbC™ Total Antibody Compensation Bead Kit) to each labeled tube.

5.6. Add a pre-titrated amount of antibody conjugate to the appropriate tube and mix well. Make sure to deposit the antibody directly to the bead suspension.

5.7. Incubate for 30 minutes at room temperature, **protected from light**.

5.8. Add 3 mL of PBS or other buffer to each sample tube. Centrifuge at 300 × *g* for 5 minutes to collect beads.

5.9. Carefully remove all supernatant from each tube.

Note: If using the red fluorescent reactive dye, repeat step 5.8 for that tube.

5.10. Resuspend bead pellet by adding 0.5 mL of staining buffer to each sample tube.

5.11. Add one drop of negative beads (Component B in the AbC™ Total Antibody Compensation Bead Kit) to sample tube(s) containing the AbC™ capture beads.

5.12. Add one drop of ArC™ negative beads (Component B in the ArC™ Amine Reactive Compensation Bead Kit) to sample tube(s) containing the ArC™ reactive beads.

5.13. Vortex tubes before analyzing using flow cytometry.

5.14. Perform manual or automatic compensation according to the preferred procedure for the flow cytometer in use. Gate on the bead singlet population based on FSC and SSC characteristics.

References

1. J Immunol Methods 313, 199 (2006). **2.** Blood 111, 1344 (2008). **3.** J Immunol 180, 774 (2008).

Product List Current prices may be obtained from our website or from our Customer Service Department.

Cat. no.	Product Name	Unit Size
L34961	LIVE/DEAD™ Fixable Blue Dead Cell Stain Kit *for UV excitation* *80 assays*	1 kit
L23105	LIVE/DEAD™ Fixable Blue Dead Cell Stain Kit *for UV excitation* *200 assays*	1 kit
L34962	LIVE/DEAD™ Fixable Blue Dead Cell Stain Kit *for UV excitation* *400 assays*	1 kit
L34963	LIVE/DEAD™ Fixable Violet Dead Cell Stain Kit *for 405 nm excitation* *80 assays*	1 kit
L34955	LIVE/DEAD™ Fixable Violet Dead Cell Stain Kit *for 405 nm excitation* *200 assays*	1 kit
L34964	LIVE/DEAD™ Fixable Violet Dead Cell Stain Kit *for 405 nm excitation* *400 assays*	1 kit
L34965	LIVE/DEAD™ Fixable Aqua Dead Cell Stain Kit *for 405 nm excitation* *80 assays*	1 kit
L34957	LIVE/DEAD™ Fixable Aqua Dead Cell Stain Kit *for 405 nm excitation* *200 assays*	1 kit
L34966	LIVE/DEAD™ Fixable Aqua Dead Cell Stain Kit *for 405 nm excitation* *400 assays*	1 kit
L34967	LIVE/DEAD™ Fixable Yellow Dead Cell Stain Kit *for 405 nm excitation* *80 assays*	1 kit
L34959	LIVE/DEAD™ Fixable Yellow Dead Cell Stain Kit *for 405 nm excitation* *200 assays*	1 kit
L34968	LIVE/DEAD™ Fixable Yellow Dead Cell Stain Kit *for 405 nm excitation* *400 assays*	1 kit
L34969	LIVE/DEAD™ Fixable Green Dead Cell Stain Kit *for 488 nm excitation* *80 assays*	1 kit
L23101	LIVE/DEAD™ Fixable Green Dead Cell Stain Kit *for 488 nm excitation* *200 assays*	1 kit
L34970	LIVE/DEAD™ Fixable Green Dead Cell Stain Kit *for 488 nm excitation* *400 assays*	1 kit
L34791	LIVE/DEAD™ Fixable Red Dead Cell Stain Kit *for 488 nm excitation* *80 assays*	1 kit
L23102	LIVE/DEAD™ Fixable Red Dead Cell Stain Kit *for 488 nm excitation* *200 assays*	1 kit
L34972	LIVE/DEAD™ Fixable Red Dead Cell Stain Kit *for 488 nm excitation* *400 assays*	1 kit
L34973	LIVE/DEAD™ Fixable Far Red Dead Cell Stain Kit *for 633/635 nm excitation* *80 assays*	1 kit
L10120	LIVE/DEAD™ Fixable Far Red Dead Cell Stain Kit *for 633/635 nm excitation* *200 assays*	1 kit
L34974	LIVE/DEAD™ Fixable Far Red Dead Cell Stain Kit *for 633/635 nm excitation* *400 assays*	1 kit
L34975	LIVE/DEAD™ Fixable Near-IR Dead Cell Stain Kit *for 633/635 nm excitation* *80 assays*	1 kit
L10119	LIVE/DEAD™ Fixable Near-IR Dead Cell Stain Kit *for 633/635 nm excitation* *200 assays*	1 kit
L34976	LIVE/DEAD™ Fixable Near-IR Dead Cell Stain Kit *for 633/635 nm excitation* *400 assays*	1 kit
L34960	LIVE/DEAD™ Fixable Dead Cell Stain Sampler Kit *for flow cytometry* *320 assays*	1 kit
Related Products		
A10497	AbC™ Total Antibody Compensation Bead Kit *for flow cytometry* *100 tests*	1 kit
A10513	AbC™ Total Antibody Compensation Bead Kit *for flow cytometry* *25 tests*	1 kit
A10344	AbC™ Anti-Mouse Bead Kit *for mouse antibody capture* *for flow cytometry* *100 tests*	1 kit
A10346	ArC™ Amine Reactive Compensation Bead Kit *for use with amine reactive dyes * *for flow cytometry compensation* *100 tests*	1 kit
L7013	LIVE/DEAD™ Reduced Biohazard Cell Viability Kit #1 *green and red fluorescence* *100 assays*	1 kit
GAS-003	Fixation and Permeabilization, 1 × 5 mL *for 50 tests*	50 tests
GAS-004	Fixation and Permeabilization, 4 × 5 mL *for 200 tests*	200 tests
GAS001S-100	Fixation Medium - Bulk, (MEDIUM A),1 × 100 mL Fixation Medium	1000 tests
GAS002S-100	Permeabilization Medium - Bulk, (MEDIUM B),1 × 100 mL Permeabilization Medium	1000 tests
FB001	IC Fixation Buffer	100 mL
PB001	IC Permeabilization Buffer	2 × 125 mL

Purchaser notification

These high-quality reagents and materials must be used by, or directly under the supervision of, a technically qualified individual experienced in handling potentially hazardous chemicals. Read the Safety Data Sheet provided for each product; other regulatory considerations may apply.

Obtaining support
For the latest services and support information for all locations, go to **thermofisher.com/support**.

At the website, you can:
- Access worldwide telephone and fax numbers to contact Technical Support and Sales facilities
- Search through frequently asked questions (FAQs)
- Submit a question directly to Technical Support (**thermofisher.com/support**)
- Search for user documents, SDSs, vector maps and sequences, application notes, formulations, handbooks, certificates of analysis, citations, and other product support documents
- Obtain information about customer training
- Download software updates and patches

SDS
Safety Data Sheets (SDSs) are available at **thermofisher.com/support**.

Certificate of Analysis
The Certificate of Analysis provides detailed quality control and product qualification information for each product. Certificates of Analysis are available on our website. Go to **thermofisher.com/support** and search for the Certificate of Analysis by product lot number, which is printed on the product packaging (tube, pouch, or box).

Limited Product Warranty
Life Technologies Corporation and/or its affiliate(s) warrant their products as set forth in the Life Technologies' General Terms and Conditions of Sale found on Life Technologies' website at **www.lifetechnologies.com/termsandconditions**. If you have any questions, please contact Life Technologies at **www.lifetechnologies.com/support**.

For Research Use Only. Not for use in diagnostic procedures.

Disclaimer
TO THE EXTENT ALLOWED BY LAW, LIFE TECHNOLOGIES AND/OR ITS AFFILIATE(S) WILL NOT BE LIABLE FOR SPECIAL, INCIDENTAL, INDIRECT, PUNITIVE, MULTIPLE OR CONSEQUENTIAL DAMAGES IN CONNECTION WITH OR ARISING FROM THIS DOCUMENT, INCLUDING YOUR USE OF IT.

Important Licensing Information
These products may be covered by one or more Limited Use Label Licenses. By use of these products, you accept the terms and conditions of all applicable Limited Use Label Licenses.

Corporate entity
Life Technologies I Carlsbad, CA 92008 USA I Toll free in USA 1.800.955.6288

All trademarks are the property of Thermo Fisher Scientific and its subsidiaries, unless otherwise specified.

©2016 Thermo Fisher Scientific Inc. All rights reserved.

ThermoFisher SCIENTIFIC

TruStain FcX™ (anti-mouse CD16/32) Antibody

**Catalog# / Size**	101319 / 50 µg<br>101320 / 500 µg
**Clone**	93
**Regulatory Status**	RUO
**Other Names**	Fcγ R III/II, Ly-17
**Isotype**	Rat IgG2a, λ
**Description**	CD16 is the low affinity IgG Fc receptor III (FcR III) and CD32 is FcR II. CD16/CD32 are expressed on B cells, monocytes/macrophages, NK cells, granulocytes, mast cells, and dendritic cells. The Fc receptors bind antibody-antigen immune complexes and mediate adaptive immune responses. TruStain FcX™ is specific to the common epitope of CD16/CD32. It is useful for blocking non-specific binding of immunoglobulin to the Fc receptors.

Product Details

**Verified Reactivity**	Mouse
**Antibody Type**	Monoclonal
**Host Species**	Rat
**Formulation**	Phosphate-buffered solution, pH 7.2, containing 0.09% sodium azide.
**Preparation**	The antibody was purified by affinity chromatography.
**Concentration**	0.5 mg/ml
**Storage & Handling**	The CD16/32 antibody solution should be stored undiluted between 2°C and 8°C.
**Application**	FC - Quality tested
**Recommended Usage**	For blocking of Fc receptors in flow cytometric analysis, pre-incubate the cells with TruStain FcX™ at 1.0 µg per 10^6 cells in 100 µl volume for 5-10 minutes on ice prior to immunostaining. It is not necessary to wash cells between these blocking and immunostaining steps.

Application References

(PubMed link indicates BioLegend citation)

1. Oliver AM, *et al.* 1999. *Hybridoma* 18:113.
2. Brummel R and Lenert P. 2005. *J. Immunol.* 174:2429. PubMed
3. Terrazas LI, *et al.* 2005. *Intl. J. Parasitology.* 35:1349.
4. Clements JL, *et al.* 2006. *J. Immunol.* 177:905. PubMed
5. Flores M, *et al.* 2008. *FASEB J.* 22:3661. PubMed
6. Ge XN, *et al.* 2010. *J. Immunol.* 185:1205. PubMed
7. Maseda D, *et al.* 2012. *J. Immunol.* 188:1036. PubMed
8. Lewis ND, *et al.* 2013. *J. Immunol.* 190:3533. PubMed
9. Bonne-Annee S, *et al.* 2013. *Infect Immun.* 81:3346. PubMed
10. Parlane NA, *et al.* 2013. *Vet Immunol Immunopathol.* 30:122. PubMed
11. Jarajapu YP, *et al.* 2014. *PLoS One.* 9:93965. PubMed
12. Matthews JA, *et al.* 2014. *PLoS One.* 9:97707. PubMed

See More

Product Citations

1. Divakaruni AS *et al.* 2018. Cell metabolism. 28(3):490-503 . PubMed
2. Contijoch EJ *et al.* 2019. eLife. 8 pii: e40553. PubMed
3. Laura C Burzynski *et al.* 2019. Immunity. 50(4):1033-1042 . PubMed
4. Axelrod HD, *et al.* 2019. Mol Cancer Res. 17:356. PubMed
5. Haupt F, *et al.* 2019. Sci Rep. 9:9798. PubMed
6. Tran NT, *et al.* 2019. Cell Rep. 28:3510. PubMed
7. Garber C, *et al.* 2019. Nat Neurosci. 1.802777778. PubMed
8. Chan LC, *et al.* 2019. J Clin Invest. 129:3324. PubMed
9. Wang X, *et al.* 2019. Cell Res. 29:787. PubMed
10. Kimura S, *et al.* 2020. Nat Commun. 0.620833333. PubMed
11. Yu X, *et al.* 2020. Nat Commun. 11:1110. PubMed
12. Zhuo Y, *et al.* 2022. J Immunother Cancer. 10:. PubMed

RRID AB_1574973 (BioLegend Cat. No. 101319)
AB_1574975 (BioLegend Cat. No. 101320)

Antigen Details

**Structure**	Ig superfamily, 40-60 kD
**Distribution**	B cells, monocyte/macrophages, NK cells, neutrophils, mast cells, dendritic cells
**Function**	Low affinity receptors for IgG
**Ligand/Receptor**	IgG
**Cell Type**	B cells, Dendritic cells, Macrophages, Mast cells, Monocytes, Neutrophils, NK cells
**Biology Area**	Immunology
**Molecular Family**	CD Molecules, Fc Receptors
**Gene ID**	14130<br>14131

Related Protocols

- Cell Surface Flow Cytometry Staining Protocol

Other Formats

Biotin anti-mouse CD16/32, FITC anti-mouse CD16/32, PE anti-mouse CD16/32, Purified anti-mouse CD16/32, Ultra-LEAF™ Purified anti-mouse CD16/32, Alexa Fluor® 647 anti-mouse CD16/32, PE/Cyanine7 anti-mouse CD16/32, TruStain FcX™ (anti-mouse CD16/32), PerCP/Cyanine5.5 anti-mouse CD16/32, APC anti-mouse CD16/32, APC/Cyanine7 anti-mouse CD16/32, Brilliant Violet 421™ anti-mouse CD16/32, Brilliant Violet 510™ anti-mouse CD16/32, Purified anti-mouse CD16/32 (Maxpar® Ready), Brilliant Violet 711™ anti-mouse CD16/32, TotalSeq™-A0109 anti-mouse CD16/32, TotalSeq™-B0109 anti-mouse CD16/32, TotalSeq™-C0109 anti-mouse CD16/32

For Research Use Only. Not for diagnostic or therapeutic use.

This product is supplied subject to the terms and conditions, including the limited license, located at www.biolegend.com/terms) ("Terms") and may be used only as provided in the Terms. Without limiting the foregoing, BioLegend products may not be used for any Commercial Purpose as defined in the Terms, resold in any form, used in manufacturing, or reverse engineered, sequenced, or otherwise studied or used to learn its design or composition without express written approval of BioLegend. Regardless of the information given in this document, user is solely responsible for determining any license requirements necessary for user’s intended use and assumes all risk and liability arising from use of the product. BioLegend is not responsible for patent infringement or any other risks or liabilities whatsoever resulting from the use of its products.

BioLegend, the BioLegend logo, and all other trademarks are property of BioLegend, Inc. or their respective owners, and all rights are reserved.

8999 BioLegend Way, San Diego, CA 92121 www.biolegend.com
Toll-Free Phone: 1-877-Bio-Legend (246-5343) Phone: (858) 768-5800 Fax: (877) 455-9587

gibco

Certificate of Analysis

QC Code: GIBCO

Collagenase Type IV
lyophilized

Source:
Clostridium histolyticum

Grade: GIBCO

Lot Number: 2820870
Item Number: 17104
Expiration Date: 2025-08
Storage Temp: 2 to 8C
Originated From: 43J23727

For research use only. Not for use in diagnostic procedures.

TEST	TEST ID	SPECIFICATION	RESULT	UNITS
**Activity (Not less than)**	ACTIV0020	>=160.00	315.00	U/mg
**Appearance**	SOLAPP0016	Acceptable	Acceptable	
**Clostripain Activity**	ACTIV0028	Check and Record	1.12	U/mgdw

Read SDS.

Vendor supplied information

J. Caldwell

Quality Systems Department   Date: 24-Apr-2024

Life Technologies Corporation
3175 Staley Rd., Grand Island, New York 14072, United States

gibco® by life technologies™

Collagenase

Description

Life Technologies' group of collagenase products are purified from *Clostridium histolyticum*. They are intended for cell and tissue disaggregation. Collagenase is a protease with specificity for the bond between a neutral amino acid (X) and glycine in the sequence Pro-X-Gly-Pro. This sequence is found in high frequency in collagen. Collagenase is unique among proteases in its ability to degrade the triplehelical native collagen fibrils commonly found in connective tissue. The collagenase most commonly used for tissue dissociation is a crude preparation containing clostripiopeptidase A and a number of other proteases, polysaccharidases, and lipases. This crude preparation is ideally suited for tissue dissociation because it contains the enzyme required to attack native collagen and reticular fibers, in addition to the enzymes which hydrolyze the other proteins, polysaccharides, and lipids in the extracellular matrix of connective and epithelial tissues. Crude collagenase does exhibit lot-to-lot variability and may produce occasional toxicity. The activity of these crude collagenase preparations has been correlated with their effectiveness at dissociating specific tissue types leading to the classification of crude collagenase preparations by type. These selected types have been found to give better performance in preparation of cells from the various tissues (Table 1).

Product	Catalog No.	Amount	Storage
Collagenase:			
Type I	17100-017	1 g	2°C to 8°C
Type II	17101-015	1 g	2°C to 8°C
Type IV	17104-019	1 g	2°C to 8°C
Collagenase, lyophilized	17018-029	500 mg	2°C to 8°C

Product Use

For Research Use Only. Not for use in diagnostic procedures

Safety Information

Read the Safety Data Sheets (SDSs) and follow the handling instructions. Wear appropriate protective eyewear, clothing, and gloves. Avoid inhalation and skin contact.

Unit Definition

One protease unit liberates 1 µmol of L-leucine equivalents from collagen in 5 hours at 37°C, pH 7.5.

Table 1: Product Selection

Collagenase	Tissue / Cell type
Type I	Epithelial, Adrenal, Lung, Fat
Type II	Heart, Thyroid, Salivary, Liver, Bone, Cartilage
Type IV	Islet (insulin receptor sites)

Use

Reconstitute Collagenase

1. Add 1 mL Hank's Balanced Salt Solution (HBSS) with calcium and magnesium directly to 1 g vial of Collagenase. Vortex gently to ensure complete dissolution.
2. Transfer to a clean tube.
3. Determine volume of HBSS with calcium and magnesium required to bring collagenase solution to 100 U/µL (1000X stock solution). Rinse vial with this volume of HBSS with calcium and magnesium, and combine.
4. Filter sterilize 1000X stock solution with a low protein binding filtration unit. Use immediately or proceed to step 5.
5. Dispense into aliquots and store at –20°C to –5°C protected from light.
6. Thaw on ice prior to use. Avoid multiple freeze/thaw cycles. We recommend using collagenase at 50–200 U/mL concentration (or 0.1–0.5% W/V).

Dissociate Tissue

1. Mince tissue into 3-4 mm pieces with a sterile scalpel or scissors.
2. Wash the tissue pieces several times with HBSS containing calcium and magnesium.
3. Add sufficient HBSS with calcium and magnesium to submerge tissue. Add collagenase to 50–200 U/mL.
4. Incubate at 37°C for 4–18 hours. Increased efficiency is obtained using a rocker platform and supplementing the digest with 3 mM $CaCl_2$.
5. Disperse cells by passing through a sterile stainless steel or nylon mesh. Remaining tissue fragments may be disaggregated by addition to fresh collagenase solution and further incubation at 37°C.
6. Wash dispersed cells several times by centrifugation in HBSS w/o collagenase.
7. Resuspend cell pellet, after the final wash step, in culture medium. Determine viable cell density using a Countess® Automated Cell Counter (alternate automated or manual methods may be used).
8. Seed cells into culture vessels containing appropriate media.

Organ Perfusion

1. Add collagenase to prewarmed (37°C) HBSS with calcium and magnesium. Addition of 3 mM $CaCl_2$ increases the efficiency of dissociation.
2. Perfuse organ at preoptimized rate for the particular organ.
3. Dispersed cells and tissue fragments are separated from larger pieces by passing the perfusate through a sterile stainless steel or nylon mesh. Remaining tissue fragments may be disaggregated by addition to fresh collagenase solution and further incubation at 37°C.
4. Wash dispersed cells several times by centrifugation in HBSS w/o collagenase.

5. Resuspend cell pellet, after the final wash step, in culture medium. Determine viable cell density using a Countess® Automated Cell Counter (alternate automated or manual methods may be used).
6. Seed cells into culture vessels containing appropriate media.

Related Products

Product	Catalog No.
HBSS, calcium, magnesium, no phenol red	14025
Trypan Blue Stain	15250
Countess® Automated Cell Counter	C10227

Explanation of Symbols and Warnings

The symbols present on the product label are explained below:

Manufacturer	Catalog number	Batch code
Caution, consult accompanying documents	Consult instructions for use	Temperature Limitation

Limited Product Warranty

Life Technologies Corporation and/or its affiliate(s) warrant their products as set forth in the Life Technologies' General Terms and Conditions of Sale found on Life Technologies' website at **www.lifetechnologies.com/termsandconditions**. If you have any questions, please contact Life Technologies at **www.lifetechnologies.com/support**.

For additional technical information such as Safety Data Sheets (SDS), Certificates of Analysis, visit www.lifetechnologies.com/support
For further assistance, email **techsupport@lifetech.com**

© 2013 Life Technologies Corporation. All rights reserved. The trademarks mentioned herein are the property of Life Technologies Corporation and/or its affiliate(s) or their respective owners.
LIFE TECHNOLOGIES CORPORATION AND/OR ITS AFFILIATE(S) DISCLAIM ALL WARRANTIES WITH RESPECT TO THIS DOCUMENT, EXPRESSED OR IMPLIED, INCLUDING BUT NOT LIMITED TO THOSE OF MERCHANTABILITY, FITNESS FOR A PARTICULAR PURPOSE, OR NON-INFRINGEMENT. TO THE EXTENT ALLOWED BY LAW, IN NO EVENT SHALL LIFE TECHNOLOGIES AND/OR ITS AFFILIATE(S) BE LIABLE, WHETHER IN CONTRACT, TORT, WARRANTY, OR UNDER ANY STATUTE OR ON ANY OTHER BASIS FOR SPECIAL, INCIDENTAL, INDIRECT, PUNITIVE, MULTIPLE OR CONSEQUENTIAL DAMAGES IN CONNECTION WITH OR ARISING FROM THIS DOCUMENT, INCLUDING BUT NOT LIMITED TO THE USE THEREOF.

life technologies™

gibco

Certificate of Analysis

QC Code: GIBCO

ACK Lysing Buffer

**Lot Number:**	2766708
**Item Number:**	A10492
**Expiration Date:**	2024-12
**Storage Temp:**	15 to 30C
**Storage Instructions:**	Protect from light

For research use only. Not for use in diagnostic procedures.

TEST	TEST ID	SPECIFICATION	RESULT	UNITS
**Endotoxin Testing**	ENDO0007	0.00 - 0.50	<0.01	EU/mL
**Osmolality**	OSMO0002	280 - 320	299	mOsm/kg
**pH**	PH0003	7.0 - 7.6	7.5	
**Sterility Testing**	STERI0007	Negative	Negative	

Read SDS

Quality Systems Department   Date: 21-Dec-2023

References

- ENDO0007: Current United States Pharmacopeia, <85> Bacterial Endotoxins Test.
- OSMO0002: Thermo Fisher Scientific Specifications.
- PH0003: Thermo Fisher Scientific Specifications.
- STERI0007: Current Edition of USP, Thermo Fisher Scientific Modified.

Technical Data

FastGene™ RNA Basic / Premium kit の評価試験

評価製品　FastGene™ RNA Basic kit（DNase処理なし）
FastGene™ RNA Premium kit（DNase処理あり）

目的　FastGene™ RNA Basic kit・FastGene™ RNA Premium kitと他社RNA抽出キットを用いて抽出したRNAの収量・品質（RIN値）・純度を評価し、性能を比較する

背 景

RNAとは、リボ核酸のことであり、核酸塩基は、アデニン(A)、グアニン(G)、シトシン(C)、ウラシル(U)の４種で構成されています。RNAはDNAと比較すると構造的に不安定であるため、取扱には注意が必要であることが知られています。
一般的にRNAの抽出には、フェノール法やシリカメンブレン等のキットを使用する方法が知られています。
弊社では、オリジナルブランドFastGene™よりシリカメンブレンを使用したRNA精製キットの開発を進めてきました。その開発に当たり、「自分たちで納得できない製品は販売することはできない！」という想いの元、評価ポイント・実験スキーム・結果の解析にこだわって試験を行いました。
本技術資料では、これら評価試験の最終段階で、得られた結果を「収量・品質(RIN値)・純度」の点で評価した事例をご紹介します。

実験条件

サンプル　：20mg BL/6マウス肝臓組織（1キットにつきn=3）

［評価ポイント］

1. 収量：吸光度測定（Implen）　2. 品質(RIN)：Agilent社Bioanalyzer（RIN値）　3. 純度：リアルタイムPCR

実験手順とキットの特長

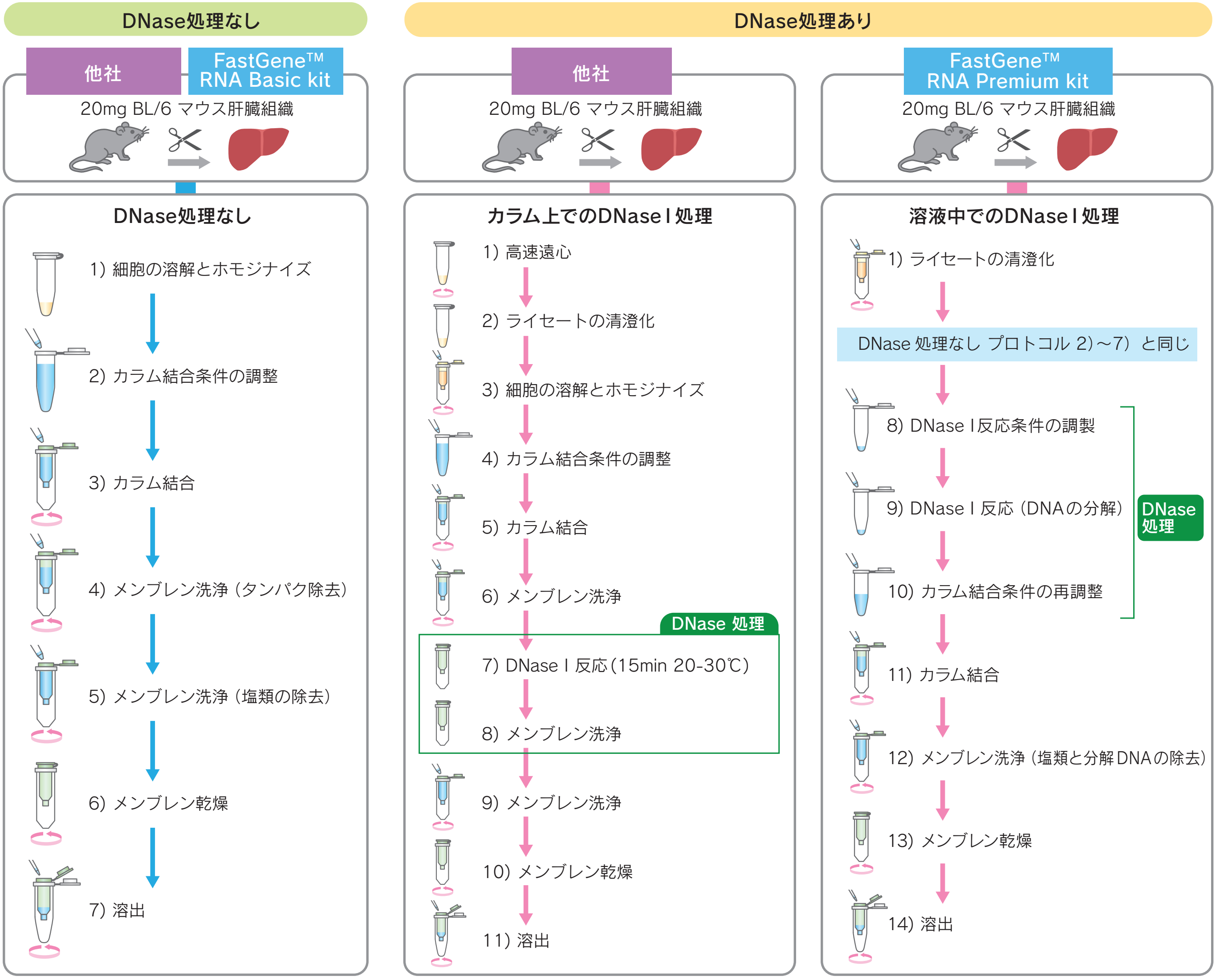

Copyright(C) NIPPON Genetics Co, Ltd All Rights Reserved.

結 果

1. 収量

FastGene™ Basic kit (DNase処理なし)

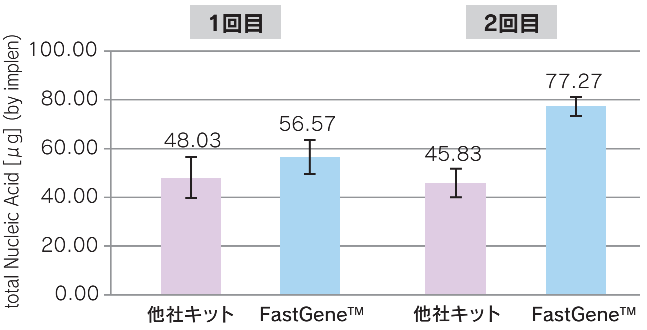

FastGene™ Premium kit (DNase処理あり)

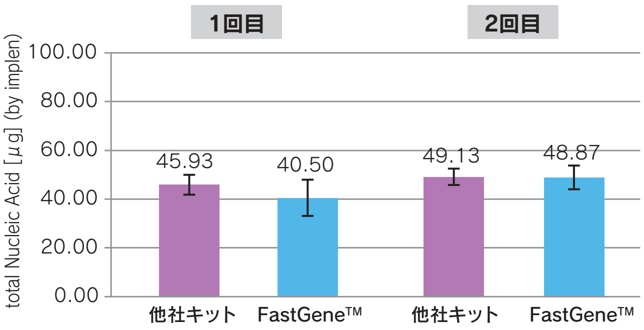

他 社 キ ッ ト ： 1回目も2回目も同じ傾向を示した。
FastGene™ ： 1回目と2回目は、若干結果が異なった。
これは、初発サンプルの違いによるものであると
考えられる。

他社キット & FastGene™：
1回目も2回目も同じ傾向を示した。

2. 品質

FastGene™ Basic kit (DNase処理なし)

再現性もあり、
なおかつ
RIN値はさらに
改善された

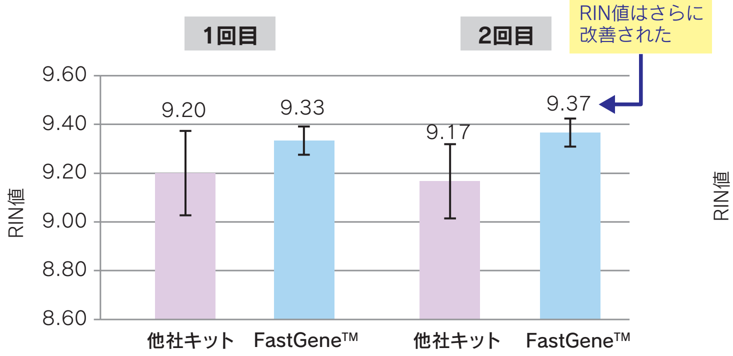

FastGene™ Premium kit (DNase処理あり)

再現性もあり、
なおかつ
RIN値はさらに
改善された

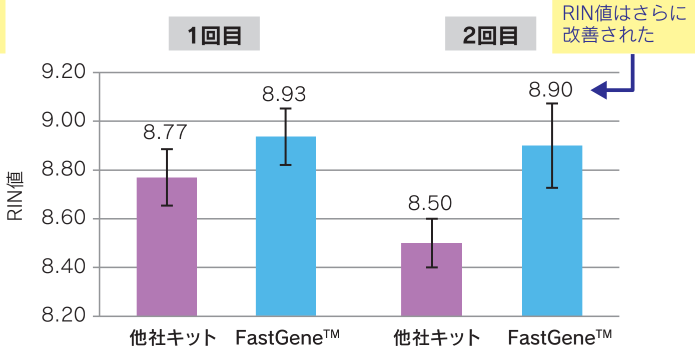

他社 & FastGene™：
1回目と2回目は同様の傾向を示した。

他社 & FastGene™：
1回目と2回目は同様の傾向を示した。DNase処理したサンプルは、DNase処理しないサンプルと比較して、RIN値が下がる傾向があったが、これは、DNase処理によるものであると考えられる。

3. 純度

リアルタイムPCRによる残留ゲノムDNA量の確認

$$残留ゲノムDNA[\%] = \frac{qPCRによる残留ゲノムDNA量[ng]}{qRT\text{-}PCRによるRNA収量[ng]}$$

FastGene™ Basic kit (DNase処理なし)

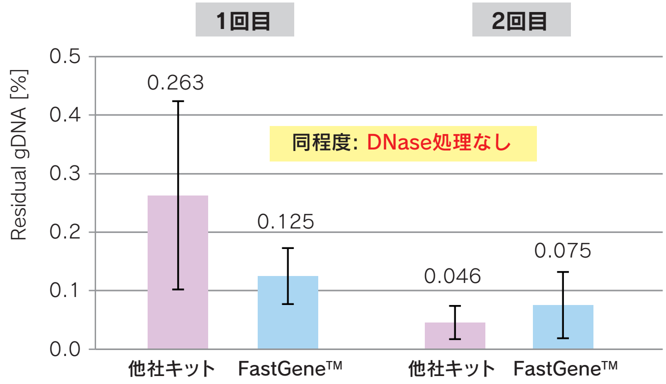

FastGene™ Premium kit (DNase処理あり)

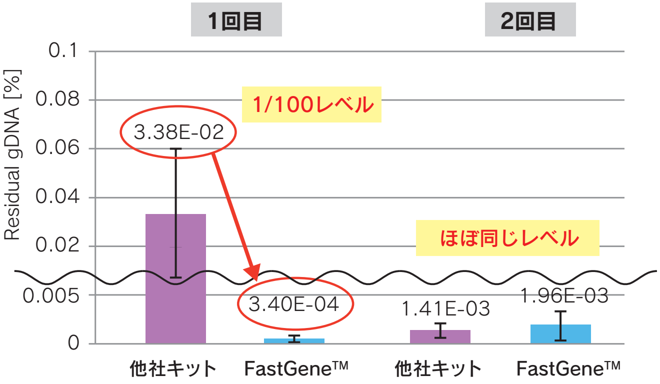

ゲノムDNAの残留率は、バッチ間で異なっていた。

1回目と2回目の結果は異なっていた。

Copyright(C) NIPPON Genetics Co, Ltd All Rights Reserved.

■ 残留ゲノムDNA量のコピー数

FastGene™ Premium kit (DNase処理あり)

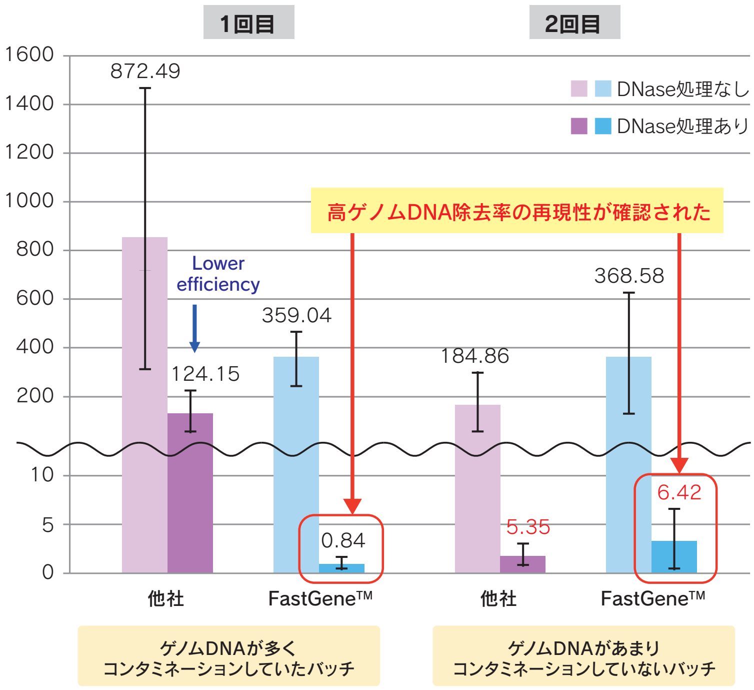

他 社 キ ッ ト ： ゲノムDNAの除去効率は、一定ではなかった。
FastGene™ ： ゲノムDNAの除去率は再現性が高く、高効率であった。

まとめ

FastGene™ RNA Basic / Premium kit は、収量・品質(RIN)・純度において、他社キットと同等、または同等以上の性能が得られました。

FastGene™ Basic kit (DNase処理なし)

- 収量 同等
- 品質 (RIN) 同等以上
- 純度 (gDNA残留率) 同等

FastGene™ Premium kit (DNase処理あり)

- 収量 同等
- 品質 (RIN) 同等以上
- 純度 (gDNA残留率) 同等以上

キットに付属のカラム

① FastGene™ RNA binding column（グリーン）

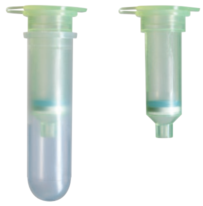

RNA結合/精製フィルター

② FastGene™ RNA filter column（イエロー）

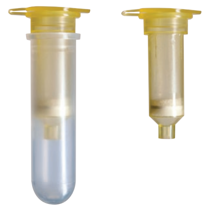

細胞残渣・高分子除去用フィルター

③ FastGene™ RNA minielute column（ホワイト）

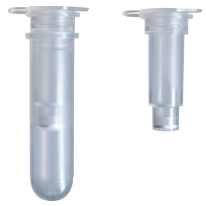

RNA濃縮用低溶出フィルター

※②と③はPremium kitのみに入っています。

Copyright(C) NIPPON Genetics Co, Ltd All Rights Reserved. 2017.FEB

日本ジェネティクス株式会社
http://www.n-genetics.com
TEL 03 (3813) 0961 FAX 03 (3813) 0962 info@genetics-n.co.jp
